# Unifying Time to Contact Estimation and Collision Avoidance across Species

**DOI:** 10.1371/journal.pcbi.1002625

**Published:** 2012-08-16

**Authors:** Matthias S. Keil, Joan López-Moliner

**Affiliations:** 1 University of Barcelona, Faculty for Psychology, Basic Psychology Department, Barcelona, Spain; 2 Research Institute for Brain, Cognition, and Behavior (IR3C), Campus Mundet, Barcelona, Spain; Université Paris Descartes, Centre National de la Recherche Scientifique, France

## Abstract

The 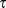-function and the 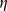-function are phenomenological models that are widely used in the context of timing interceptive actions and collision avoidance, respectively. Both models were previously considered to be unrelated to each other: 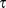 is a decreasing function that provides an estimation of time-to-contact (*ttc*) in the early phase of an object approach; in contrast, 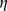 has a maximum before *ttc*. Furthermore, it is not clear how both functions could be implemented at the neuronal level in a biophysically plausible fashion. Here we propose a new framework – the *corrected modified Tau* function – capable of predicting both 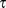-type (“
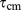
”) and 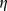-type (“
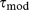
”) responses. The outstanding property of our new framework is its resilience to noise. We show that 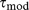 can be derived from a firing rate equation, and, as 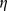, serves to describe the response curves of collision sensitive neurons. Furthermore, we show that 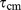 predicts the psychophysical performance of subjects determining *ttc*. Our new framework is thus validated successfully against published and novel experimental data. Within the framework, links between 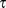-type and 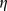-type neurons are established. Therefore, it could possibly serve as a model for explaining the co-occurrence of such neurons in the brain.

## Author Summary

In 1957, Sir Fred Hoyle published a science fiction novel in which he described humanity's encounter with an extraterrestrial life form. It came in the shape of a huge black cloud which approached the earth. Hoyle proposed a formula (“”) for computing the remaining time until contact (“*ttc*”) of the cloud with the earth. Nowadays in real science,  serves as a model for *ttc* -perception for animals and humans, although it is not entirely undisputed. For instance,  seems to be incompatible with a collision-sensitive neuron in locusts (the *Lobula Giant Movement Detector* or LGMD neuron). LGMD neurons are instead better described by the -function, which differs from . Here we propose a generic model (“”) that contains  and  as special cases. The validity of the  model was confirmed with a psychophysical experiment. Also, we fitted many published response curves of LGMD neurons with our new model and with the -function. Both models fit these response curves well, and we thus can conclude that  and  possibly result from a generic neuronal circuit template such as it is described by .

## Introduction

Monocular presentation of a looming object elicits escape or avoidance reactions in many species, including humans [1]–[4]. When a planar object travels perpendicular to a surface toward an observer (i.e. the object approaches the observer on a direct collision course), it projects a symmetrically expanding image on the retina. Notice that in the present paper we only focus on a subset of approaches where the approaching object eventually collides with the observer. We assume that collision happens at time 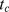 (*time to contact*, “*ttc*”). At time 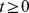 before 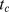, the image subtends an angle 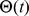, and its outer contours expand with angular velocity 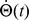. Both angular variables grow nearly exponentially with decreasing distance 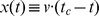 between object and eye (assuming a constant velocity 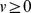). With knowledge of a predator's or object's typical size [5], it is therefore possible to trigger a behavioral response as soon as 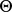 or 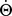, respectively, crosses a threshold [1], [6], [7].

The visual systems of various species are also known to “compute” functions of 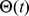 and 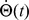 (see e.g. [8] for a recent review). The *Tau-function* (“
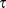
”) is defined by 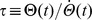. Under the assumption that the object is a rigid sphere that approaches with 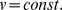, 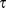 has several interesting properties [9], [10]: *First*, 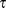 provides a running estimation of *ttc* during the approach. *Second*, the *ttc* estimation is largely independent of physical object size, provided that 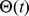 and 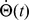 are noise-free. *Third*, 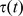 decreases approximately linearly with time with a constant slope of 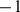, but eventually linearity is compromised, as 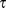 has a minimum shortly before *ttc*. It therefore would be comparatively easy to track the remaining time 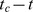 until impact, and to precisely time avoidance reactions, for example as soon as 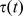 is below a certain threshold value.

These three properties, however, are valid only for “sufficiently small” angular sizes 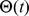. Any quantitative criterion for “sufficiently small” implicates an error threshold for the deviation of 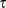 from linearity, that is 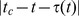. For example, according to *Text S6* a corresponding threshold for the visual angle can be defined as 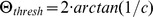 with some constant 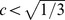. Notice that the 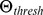-criterion is independent from stimulus parameters such as object diameter or approach velocity.

Because 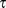 is well suited for the estimation of 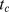, it could in principal serve as a universal mechanism for guiding motor actions during object approaches or during self-motion towards static objects. Indeed, several studies related 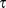 to behavioral responses in this context, thus asserting that many organisms, including humans, rely on 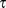 for their timing of motor actions (e.g. [10]–[12]). But a critical re-evaluation of the 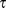-hypothesis arrived at the conclusion that 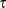 does not necessarily play a unique role for *ttc* estimation [13], [14]. For example, humans also rely on the rate of change of relative disparity, particularly in the late phase of an approach, for small object sizes [15]–[18], for low speeds [19], [20], or if knowledge of object size is available [7]. In addition, the task at hand (e.g. catching a ball or eluding a meteorite) seems to dictate the information that will eventually be used for action timing [14], [18], [21]. Further inconsistencies with respect to 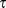 were reported with psychophysical results, where 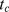 tended to be underestimated [16]. In addition, *ttc* -estimation reveals a certain dependence on object size [22], which is also not predicted by 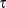 at “sufficiently small” angular sizes.

The *Tau-function* is often studied in the context of *ttc* -estimation. It appears, however, that in order to describe the responses of collision-sensitive neurons in certain species 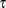 is inadequate. For example, the *Lobula Giant Movement Detector* (LGMD) neuron in locusts responds with increasing activity to a stimulation with a symmetrically expanding image, if the expansion pattern is consistent with an approaching object [23], [24]. The response curve of the LGMD neuron gradually increases to a maximum and then abruptly ceases (often to a nonzero baseline response). Because 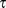 does not have a maximum, a different function has been proposed for modeling LGMD responses: The *Eta-function* (“
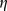
”). It is defined as 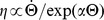, with a constant 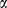 [25]. Theoretically, the time when the activity peak occurs depends linearly on the ratio 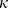 of object half-size 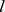 to object velocity 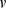. The peak will shift closer to 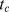 for smaller or faster objects, and always occurs at angular size 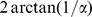, independent of 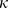 [26]. The LGMD activity peak could in principle signal a critical angular size for escaping. Indeed, a recent study with freely behaving locust suggests that the time of peak firing rate of the *Descending Contralateral Movement Detector* (DCMD) predicts that of jump [27] (each LGMD spike triggers a spike in the postsynaptic DCMD as well, because the LGMD is strongly coupled to the DCMD by a combined electrical and chemical synapse [28], [29]).

It has nevertheless been argued that – in some ecologically meaningful situations (small 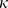) – there is no guarantee for the peak to occur *before* 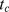 [2], [5]. This statement may be true to the extent that in freely behaving locusts, a reliable escape jump is triggered *before* collision only in the range of 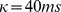 to 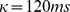 [30]. For 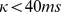, the jump would occur *after* projected collision, and this value thus may reflect the typical sizes and speeds of predators.

Apart from the locust, other species have collision-sensitive neurons with 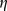-like properties, for instance fruitflies [31] and bullfrogs [32]. In pigeons, the response properties of one of three classes of neurons in the dorsal posterior zone of the *nucleus rotundus* also seems to be compatible with the 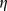-function [1]. (The two remaining classes seem to compute 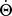 and 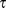, respectively). In the goldfish, responses of the M-cell to looming stimuli also appear to follow a version of the 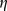-function, in which 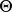 replaces 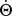, such that the new function does only depend on 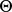 [33].

The *Tau-function* and the *Eta-function* are the two prevailing models for studying *ttc* -perception and (interceptive) action timing on the one hand, and escape behavior and collision avoidance on the other. In other words, we have two different models for two seemingly separated contexts. Each model brings about some hitherto unresolved issues, which are subsequently described.

From a computational point of view, 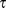 is numerically unstable: In the presence of noise, we have to reckon with the fact that 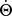 can get very small – or even reach zero – at certain instants during the initial phase of the approach (cf. [17]). As a consequence, fluctuations of 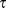 with large amplitudes may occur. If, however, noise levels are constant in time, and noise is not multiplicative, the signal to noise ratio continuously improves as 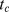 is approached. It is furthermore not entirely clear how 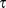 could be biophysically implemented in a neuron.

As for the 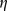-function, the LGMD neuron seems to bypass a direct multiplication or division by computing 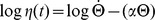 with subsequent exponentiation of the result [34]. From a mathematical viewpoint, however, taking the logarithm introduces an instability for 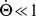, although neuronal circuits with divisive inhibition can be adjusted such that no stability problems occur [35]. Moreover, *Gabbiani et al.* [34] found that a third-order power law fitted the mean instantaneous firing rate of the LGMD better than an exponential or a linear function (see also reference [36]).

Our original motivation was to improve the stability of 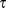 with a simple modification. This modification led us to the *modified Tau* function 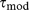. Similar to the 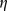-function, the 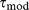-function also reveals a maximum before *ttc*. We were able to fit the response curves of 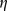-type neurons with 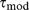 (*Text S4*). Our 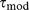-function represents the equilibrium solution of an equation for describing neuronal firing rate. Because of this, 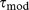 is based on a biophysically plausible mechanism.

But 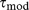 comes with a disadvantage: Unlike 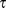, it no longer provides a running value of *ttc*. In order to recover the *ttc* prediction, we needed to add a correction term to 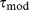. This so-defined *corrected modified Tau* function (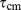) recovers the *ttc* prediction of the original 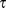-function, but suppresses noise better than 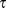. Most importantly, the *corrected m-Tau* function predicts the results of a psychophysical experiment, requiring subjects to estimate *ttc*.

Theoretically, we therefore can explain 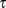-type and 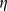-type responses within the 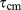 framework, which contains 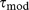 (but also 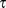
!) as a special case. Until now, 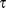 and 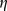 did not have any obvious relationship with each other (although we show in *Text S6* how 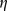 could formally be related to 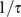). The 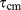-function could thus serve to explain why 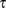-type and 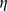-type neurons could be found alongside each other in the pigeon brain [1].

## Results

The *corrected modified Tau* function “
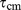
” (equation 5) contains the *modified Tau* function “
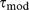
” (equation 1) as a special case. We nevertheless first introduce the 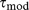 model, as this makes its relation to the original 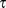-function much easier understood.

### The modified 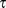 model (“
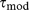
”)

Behavioral and neural responses to optical variables (e.g., 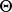, 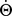, 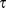, 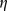) in the initial part of a trajectory are very noisy signals. Signal fluctuations may occur as a consequence of the discrete structure of the retinal photoreceptor array and its limited spatial resolution. The signal-to-noise ratio continuously improves as *ttc* is approached (*Text S3*).

**Figure 1:**
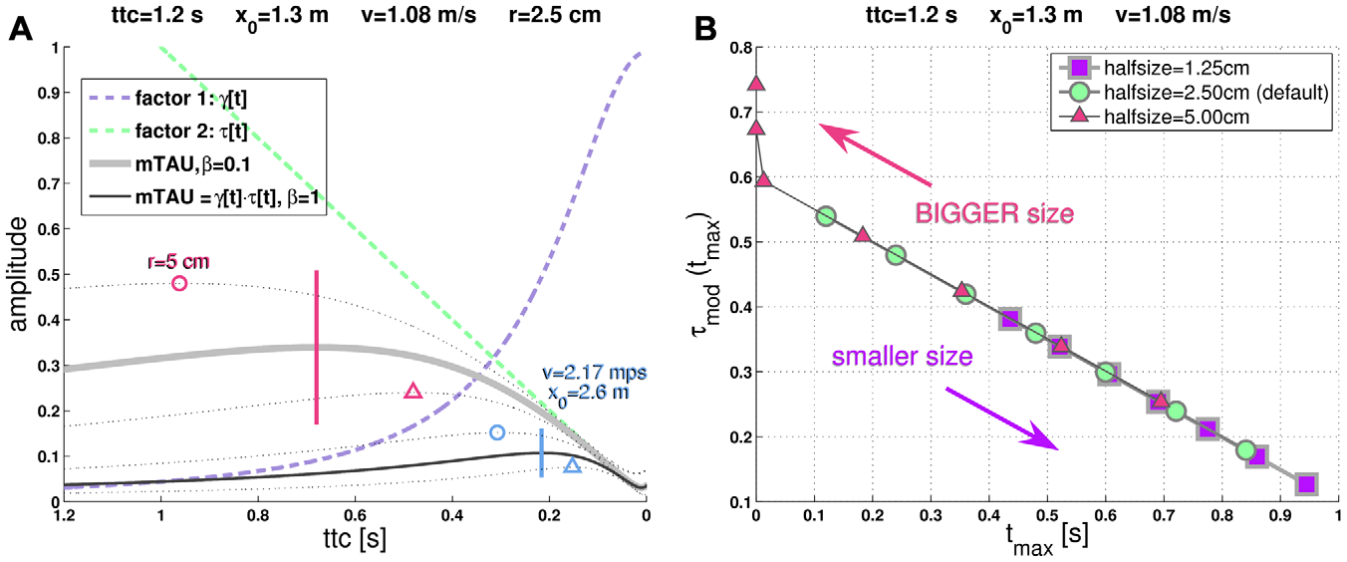
The modified Tau function (“m-Tau”). (**a**) The figure shows two m-Tau functions which are distinguished by 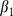 (with values 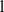 and 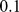, see legend). The horizontal bars denote their respective maxima for the default stimulus values (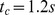, 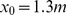, 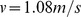, 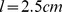). The maxima shift to the left (circles) upon doubling the object radius 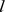 to 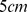 (“size effect”). They shift in the opposite direction (triangles) upon doubling both the approach velocity 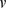 and the initial distance 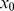 (“velocity effect”), such that 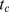 remains unchanged (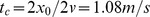). The thin dotted lines (not identified in the legend) show the m-Tau functions with correspondingly doubled values. For the m-Tau function with 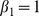, the two factors 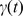 and 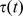 are furthermore plotted, see equation (1). The shift directions of the maxima are identical with the corresponding shifts observed with the 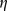-function, see *Text S1*. (**b**) Here it is shown how the maxima of seven m-Tau functions shift when the object diameter is halved or doubled with respect to its default value 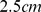. Each point indicates 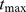 (time of maximum) along with its corresponding amplitude 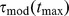. Circular symbols represent the default case with 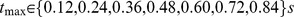. All maxima lie on a line. With a smaller object diameter all maxima shift to the right (towards 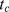), and an increase in object size causes a shift of all maxima to the left (away from 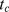). All shifts proceed along the same straight line. Notice that some artifacts occur for the two leftmost points, because all maxima were computed numerically. The velocity effect is illustrated in *Text S1*.

Our first step adds computational stability to the 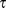 model. Let 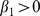 be a constant (in units of 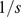). The *modified Tau* model is defined as:
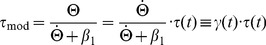
(1)Biophysically, 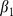 can be interpreted as leakage conductance (equation S2 in *Text S1*). According to equation (1), 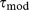 can formally be expressed in terms of 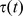 multiplied with a gain control factor 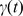, which depends only on angular velocity. Notice, however, that the multiplicative version “
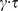
” would again compromise stability, because 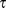 appears as one of the factors in the product. Figure 1 *a* juxtaposes 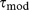 and the factors 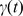 and 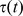, respectively.

Let the initial distance between the eye and a circular object (diameter 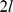) be denoted by 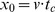. Then, choosing 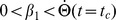 will create a maximum of 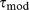 at time 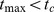 (i.e., a maximum *before* 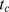):
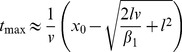
(2)(the previous equation is derived in the Methods Section). The time 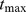 when 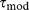 assumes its maximum can thus be controlled by specifying 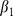, where bigger values will place the maximum closer to 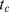. The maximum depends as follows on approach velocity and object diameter, respectively.

Assume fixed values for 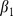 and 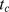. Then, 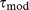 will have an activity maximum at 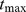 (default case). Now increase approach velocity and initial distance, such that 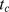 remains constant. As a consequence, the peak will shift closer to 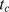 with respect to the default case (triangle symbols in Figure 1*a*; further figures in *Text S2*). This is the *velocity effect*.

Now increase the object diameter. The maximum of 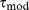 will then occur earlier compared to the default case (circle symbols in Figure 1). This is the *size effect*.

Assuming that the peak signals an imminent collision, this shifting behavior is consistent with larger objects being perceived to have an earlier *ttc* than smaller ones [22]. Note that the original 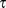-function (i.e. 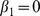 and noise-free angular variables) does not show a strong dependence on object size where 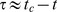 holds (but see *Text S6*).

**Figure 2:**
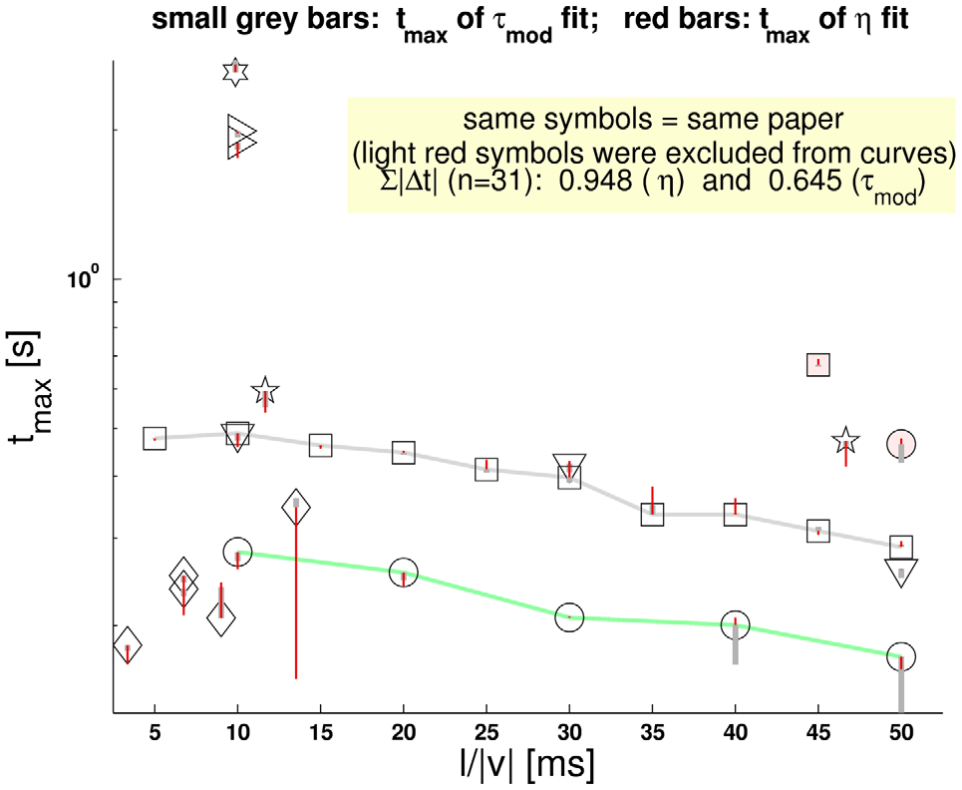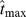 from experiments (symbols) compared to fitted 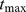 (bars). All symbols indicate the maxima 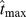 in the neuronal recording data as a function of 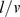 (with 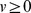). These data were manually resampled from previously published studies (see *Text S4* for further details). The line ends (lines start at the center of each symbol) denote where the fitted functions 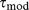 (thick gray bars) and 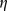 (thin and red bars) have their respective maxima. Thus, the longer a bar, the bigger the difference between the predicted maxima and that of the neuronal data. The respective sum of absolute differences is indicated in the inset. The mean (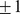 s.d., 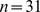) of absolute differences is 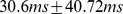 (median 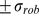: 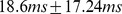) for the 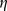-function, and 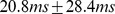 (median 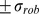: 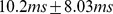) for 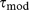. The two continuous lines connect the data for a series of 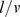 values from the same paper (light gray: reference [26]; green: reference [39]; first figure in *Text S4*: all references.)

The 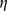-function is the prevailing model for describing responses from collision sensitive neurons to object approaches with constant velocity. Its characteristic feature is its maximum. Because 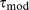 also has a maximum, we fit 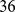 previously published neuronal response curves with the 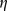-function and 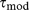 (*Text S4*). Figure 2 summarizes these fits by comparing the response maxima of the experimental curves (“
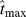
”) with the maxima predicted by the best fits achieved with the two functions (“
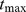
”). Predictions of 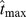 are slightly better with 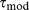-fits, both in terms of mean and median of absolute differences (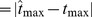). With respect to goodness of fit measures (root-mean-square-errors, 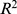, F-statistics), both functions perform again on par with each other. Therefore, both 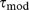 and the 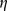-function describe neuronal responses of object approaches with constant velocity.

**Figure 3:**
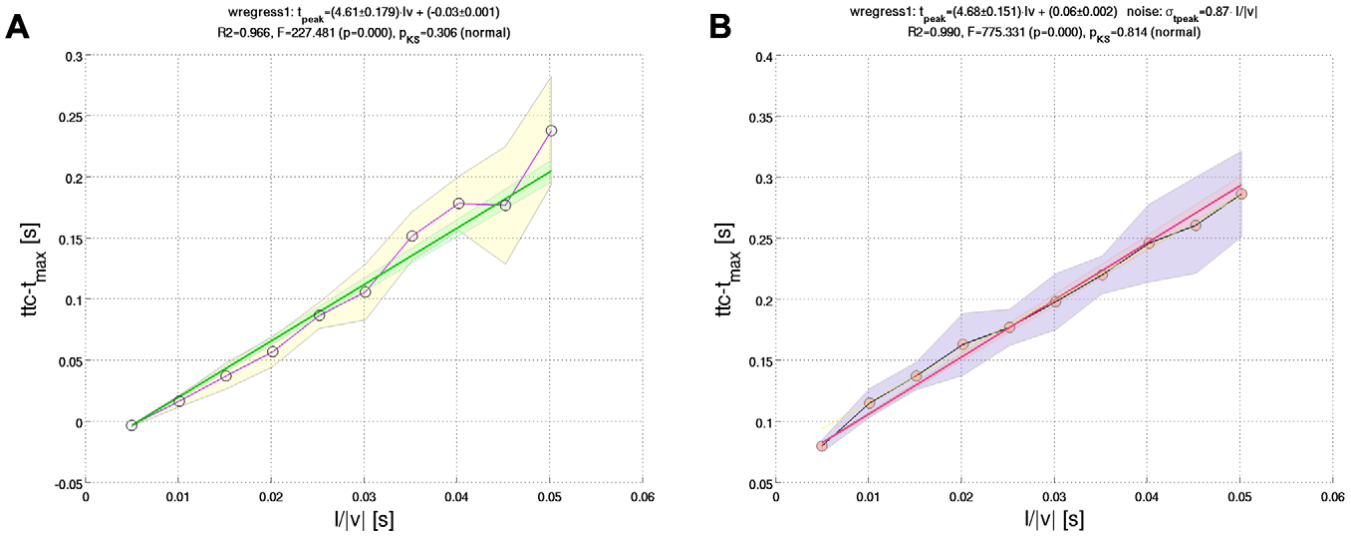
Masking of the m-Tau nonlinearity by noise. The experimental data from *Gabbiani et al.* [26] suggest a linear relationship between relative time of peak firing rate 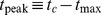 and the half-size to velocity ratio 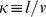. The big shaded areas indicate one standard deviation 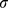 from the mean value of 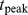. Notice the increase in 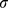 with increasing 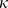. (**a**) Resampled Figure 4a from Reference [26] (p. 1128). The locusts were stimulated by approaching dark squares with different sizes and velocities, such that various values of 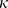 were covered. The circle symbol for each 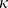 represents the mean 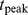 of neuronal response curves across 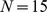 DCMD neurons. The result of a weighted least square regression fit reported by *Gabbiani et al.* had slope 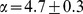 and intercept 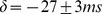. With the manually resampled data points shown here, we obtained 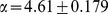 and 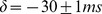, respectively. The light green shaded area indicates one standard deviation of slope. Additional statistical parameters of our weighted least square fit are shown above the figure. (**b**) An example of fitting a straight line to 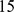 averaged random trials of the “noisified” equation (2) with 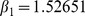. “Noisifying” means that Gaussian noise with standard deviation 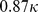 was added to 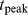 (according to equation 8, page 1129 in [26]). The noise blurs the nonlinear character of the m-Tau function and makes it *appear* linear. The light red shaded area indicates one standard deviation of slope. Further simulation results are presented in *Text S2*.

The experimental maxima at time 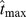 depend linearly on 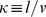 [26]. The 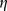-function predicts this linear relationship (equation S5 in *Text S2*), where slope is identified by 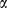, and intercept by a temporal delay 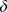 of corresponding line fits (Figure 3*a*).

**Figure 4:**
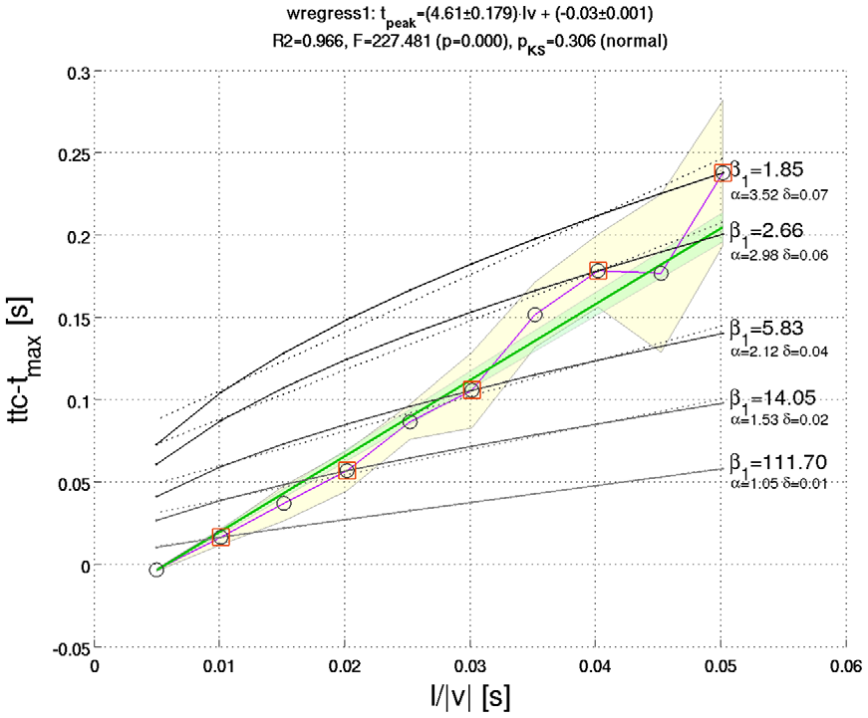
Illustration of nonlinear dependence of m-Tau maxima. The red square symbols denote data points 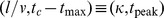, according to Figure 3*a* from reference [26]). In order to illustrate the nonlinear behavior of m-Tau, for each of these points an instance of m-Tau was created, such that the peaks of the 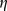-function and the m-Tau function coincide. The corresponding values of 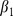 were computed with equation S7 in *Text S2*, and are indicated in the figure. Along with the 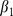, the values of 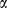 and 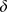 are shown in small font size. The latter two values were obtained by “brute-force” fitting a straight line to the nonlinear m-Tau curves. We observe that: *(i)* the curvature of m-Tau (equation S6 in *Text S2*) increases with decreasing values of 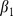. *(ii)* All “slopes” of the “brute-force” line-fit to m-Tau are smaller than suggested by the data from *Gabbiani et al.*, who reported 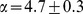 (our fit of their re-sampled data is indicated by the green line and yielded 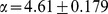; see figure headline).

The maximum of the 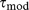-function 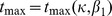, however, depends in a nonlinear way on 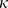 (equation 2 & equation S6 in *Text S2*; illustration: Figure 4). (Nonlinearity means that the slope depends on 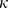, and linearity means that it does not). Linearity is approached with increasing values of 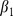, eventually reaching a slope of one for 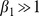 (equation S9 in *Text S2*). This is nevertheless inconsistent with experimental evidence, as the experimental values for 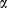 are underestimated (typically 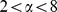).

We thus explored a different possibility: Can the nonlinear function 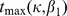 be hidden by noise? Figure 3*b* suggests that it nearly can, as seen when fitting a line to a version of 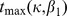 with additive Gaussian noise. Noise levels were set as reported in [26]. This hide-and-seek works quite well, and the fitting statistics (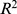, KS-test on residuals, F-statistics) are consistent with linearity in many random trials (detailed analysis: *Text S2*).

**Figure 5:**
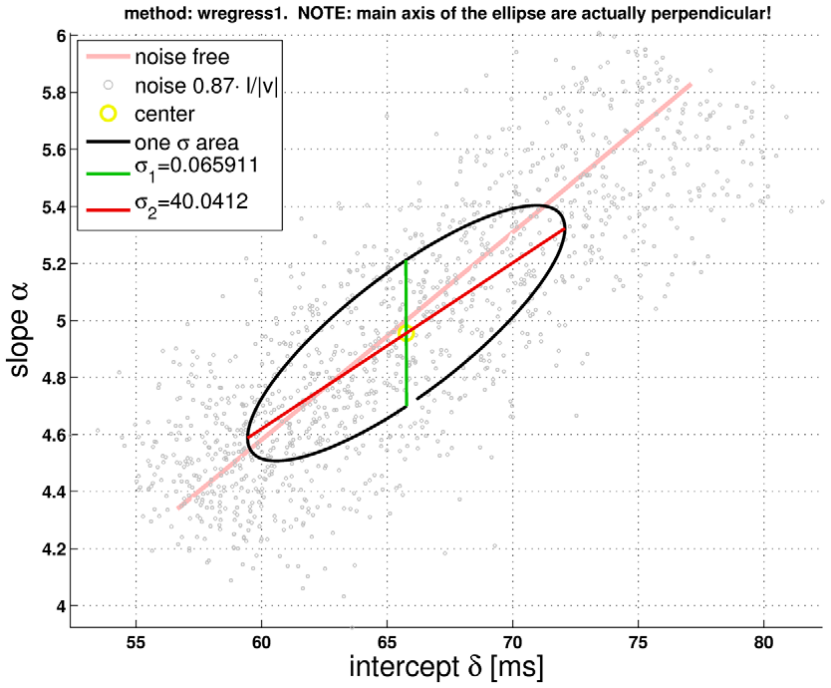
Simulation of **Figure 4b** from Reference [**26**] (p. 1128). For compiling this figure, a value of 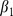 was first selected. Then, 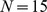 noisified curves 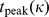 (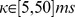) were generated and averaged, assuming a noise level of 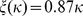 in equation S10 in *Text S2* [26]. A pair of intercept and slope values (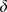 and 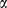, respectively) were obtained from a weighted linear regression fit to the average curve (weights 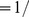
variance). Now, 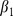 was parsed from 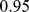 to 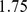 in steps of 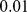 (totaling 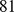 values). For each value of 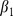, the weighted linear regression fit to the averaged 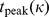-curves was repeated 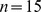 times. The small grey circles represent the mean value of these 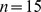 intercept-slope pairs. Statistical parameters for each fit were also recorded, and the corresponding figures are included in *Text S2*. The main axis of the ellipse are in the direction of the eigenvectors of the covariance matrix. The matrix was computed from all intercept-slope pairs (i.e. 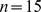 samples for each 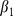). The lengths of the eigenvectors were scaled with the square root of their associated eigenvalues. The area enclosed by the ellipse thus corresponds to one standard deviation (legend: 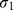 and 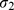). (Note that the ellipse shown in Figure 4b from *Gabbiani et al.* denotes instead a 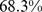 confidence region for intercept and slope). The noise-free correlation is indicated by the straight line. Notice that the abscissa values are defined up to an arbitrary additive constant.

Figure 4 suggests a correlation between intercept and slope of line fits for different values of 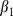. We thus fit lines to the noisified version of 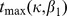 for various values of 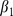. As before, noise levels were set as reported, and we again identified intercept and slope of the line fits to 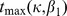 with 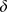 and 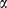, respectively. The result of this procedure is shown in Figure 5, and agrees well with Figure 4 in [26]. Thus, 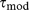 consistently predicts a good correlation between intercepts and slopes both in the presence and in the absence of noise.

### The corrected modified 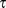 model (“
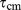
”)

Maximum detection of 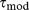 in the initial phase of an object approach (i.e., for small values of 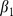) is problematic, due to the signal's poor signal-to-noise ratio and the rather “shallow” curvature around the maximum. The situation gets progressively better if we place the maximum closer to 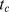, that is for bigger values of 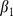: The signal-to-noise ratio is better, and curvature is higher. With 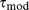, however, we fell short of explaining the results of our psychophysical experiment (which is below described further). This led us to modify 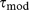 as follows.

Observe that 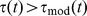 for all 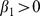, and thus
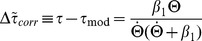
(3)is a positive correction factor to 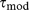, such that 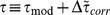. As with 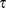, the correction factor 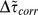 *per se* is again susceptible to fluctuations in the angular variable 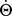, and we would have gained no improvement by simply adding it to 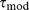.

Now, the crucial idea is to render 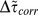 insensitive to such fluctuations. This is achieved with a first order low-pass filter (a short introduction is given in *Text S8*). Low-pass filtering of 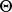 and 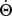 transforms 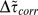 into a slowly varying signal, which is eventually added to 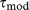:
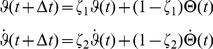
(4)

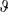
 and 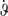 are low-pass filtered visual angle and angular velocity, respectively, and 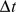 is the system's integration time constant. In order to avoid initial filter transients, the filter variables were initialized with 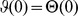 and 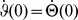, respectively. The 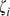 are filter memory coefficients with 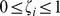 for 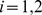. No filtering would take place for 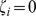 (no memory), and the filters would never change their initial state for 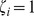 (infinite memory).

The *corrected, modified* 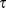 model (“*corrected m-Tau*”) is then defined as:
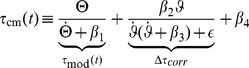
(5)where 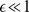 is a small constant, such that possible division-by-zero errors are avoided in the simulation. Nevertheless,in the presence of noise, division-by-zero errors do not typically represent a problem during an approach with 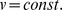, because 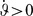 if the following two conditions hold: *(i)* appropriate initialization of 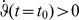, and *(ii)* “sufficiently strong” lowpass filtering. The offset 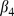 is included for the sake of completeness. It was only considered for simulating our psychophysical experiment (described below), where it turned out to be negligibly small. In general, therefore, it is safe to assume 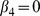.

**Figure 6:**
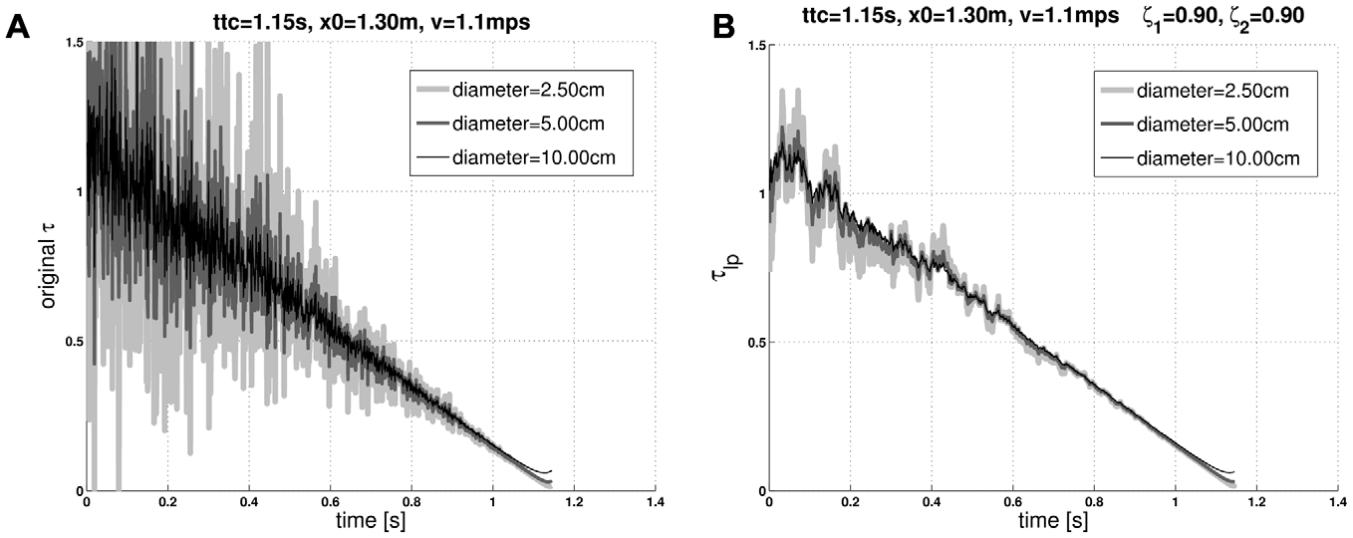
Limit functions of the *corrected m-Tau* function. The *corrected m-Tau* -function 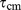 responds similar to 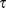, but with an improved noise suppression performance, as long as parameter values 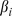 (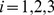 and 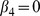) are suitably chosen. More precisely, 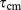 is constrained by the limit functions 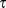 and 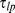. This means that *corrected m-Tau* can approach the former or the latter function for the corresponding (extreme) values of 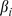, but typically 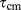 will perform somewhere between the two limit functions. For the simulations shown in this figure, uncorrelated normal-distributed noise was added to the angular variables 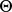 and 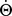. Each curve represents a typical random trial, where noise was identical for all curves. The different shades of gray indicate different object diameters, as indicated in the legends. (**a**) “Normal” 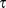 function, which is the limit function approached by 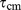 for 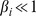. Noise suppression is poor. Notice that the displayed range has been truncated so as to match it to the range of the figure on the right-hand side. (**b**) The 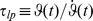 function is the limit function that is approached for 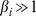. It has an excellent noise suppression performance, owing to lowpass filtering of angular variables (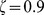, c.f. equation 4). Further details are presented in *Text S3*.

Similar to 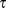, the new *corrected m-Tau*-model also computes an estimation of *ttc* for “sufficiently small” angular sizes. But the principal advantage of 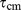 over 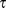 is that it is less sensitive to noise. The noise suppression of the *corrected m-Tau*-model is constrained by the noise suppression performance of two “limit functions”, which are approached dependent on the values of 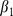, 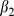, and 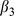 (Figure 6). For the derivation of these limit functions, assume (to simplify matters) that in equation (5) 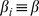 with 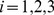 (and 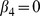). Then, as we will show subsequently, the constraining functions are the ordinary 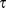 function for 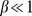, on the one hand (equation 6), and for 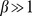 a version of 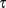 with lowpass-filtered angular variables, on the other (equation 8). Thus, 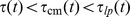, where 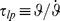, provided that we exclude the case 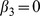, 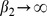, which would imply that 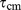 is unbounded.

#### Case I: 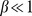


For very small 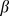 (more precisely 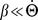), the first term of the equation (5) is approximately
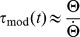
(6)which is just the ordinary 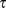 function. For the second term 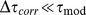, which implies that it can be neglected because its denominator is approximately equal to 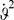. Furthermore, during an object approach with constant velocity, angular size 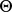 and angular velocity 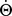 are increasing, and 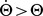, as 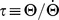 is monotonically decreasing (except at times very close to *ttc*, see *Text S6*). The last arguments hold also for 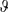 and 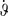, respectively, which are the lowpass-filtered optical variables, where 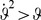. We eventually arrive at the approximation
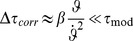
(7)Summarizing the above, if 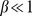, then the noise suppression performance of the *corrected m-Tau* -model is comparable with that of ordinary 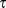 (Figure 6*a*).

#### Case II: 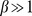


For 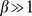 (more precisely 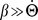), the situation is just the opposite of *Case I*. The first term of equation (5) can be neglected, because 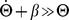. Given that 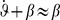 in the denominator of the 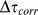 term, we obtain
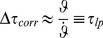
(8)This is the ordinary 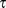-function but with lowpass filtered optical variables (“
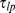
”, Figure 6*b*).

### Predicting psychophysical performance

**Figure 7:**
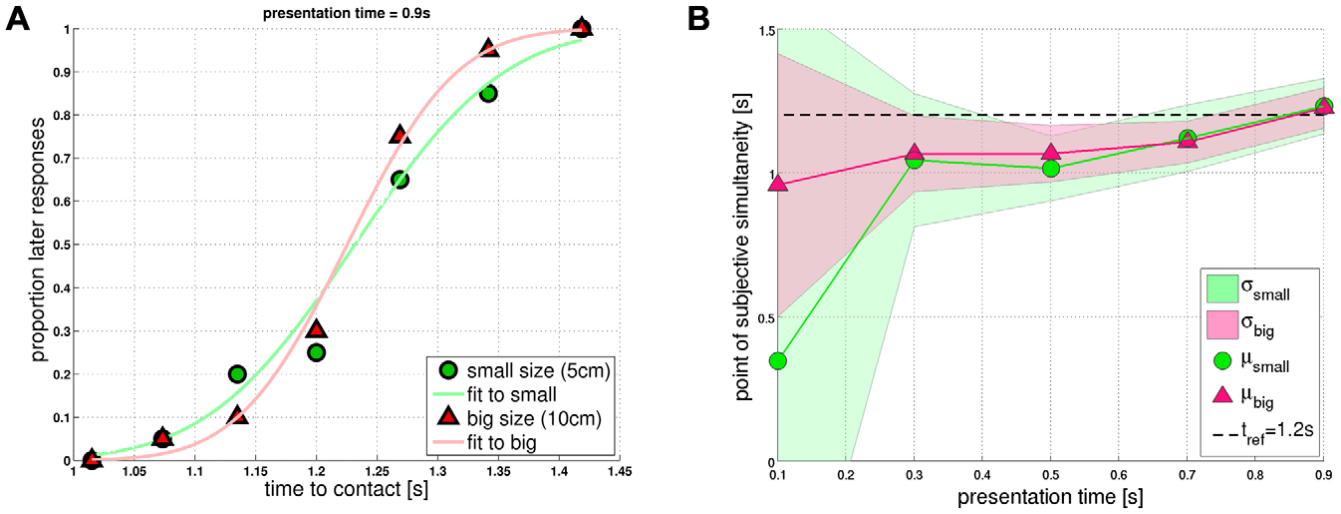
Psychometric functions. (**a**) Psychophysical data points 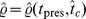 for “proportion of later responses” are shown for the presentation time 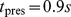 and object diameters *big* (triangle symbols) and *small* (circle symbols), respectively. Each sigmoid curve represents a fit of a Gaussian cumulative density function (“GCDF” with mean 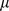 and standard deviation 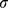) to the data points of the respective object diameter. The GCDF-fits approximate the underlying psychometric functions, with the mean 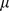 indicating the time point of subjective simultaneity. (**b**) The curves show how 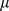 and 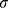 depend on presentation time and object diameter. Each point represents the result of a GCDF-fit to the psychophysical data. If subjects responded correctly, the point of subjective simultaneity would coincide with 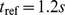 (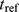 is indicated by the dashed horizontal line).

Details on our psychophysical experiment are spelled out in the Methods Section. In a nutshell, subjects viewed approaching balls on a monitor. The balls had two different sizes (*big* & *small*, corresponding to object diameters 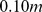 & 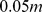, respectively), and disappeared after 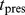 (presentation time) until 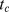. A beep sounded always at the same time, 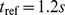, in order to indicate a reference time to the subjects. Approaches with different values of 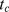 were presented, where 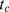 could occur before or after 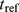. Subjects were asked to judge whether they were hit by the ball before or after 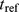. Responses were pooled, and the “proportion of later responses” for each presentation time (corresponding to “ball hit me after 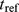
”) was computed as a function of *ttc*. Figure 7*a* shows the corresponding data points for 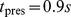, along with the best matching Gaussian cumulative density function (“GCDF”-fit) for each object diameter. The GCDF-fits represent an estimate of the underlying psychometric curves or psychometric functions, respectively. Figure 7*b* suggests that subjects did not respond to the average of the stimulus set, because the mean of the distribution (point of subjective simultaneity) shifted with presentation time. In addition, the variance of the distribution decreased with increasing presentation time. The *small* object diameter is furthermore associated with a higher variance than the *big* one.

**Figure 8:**
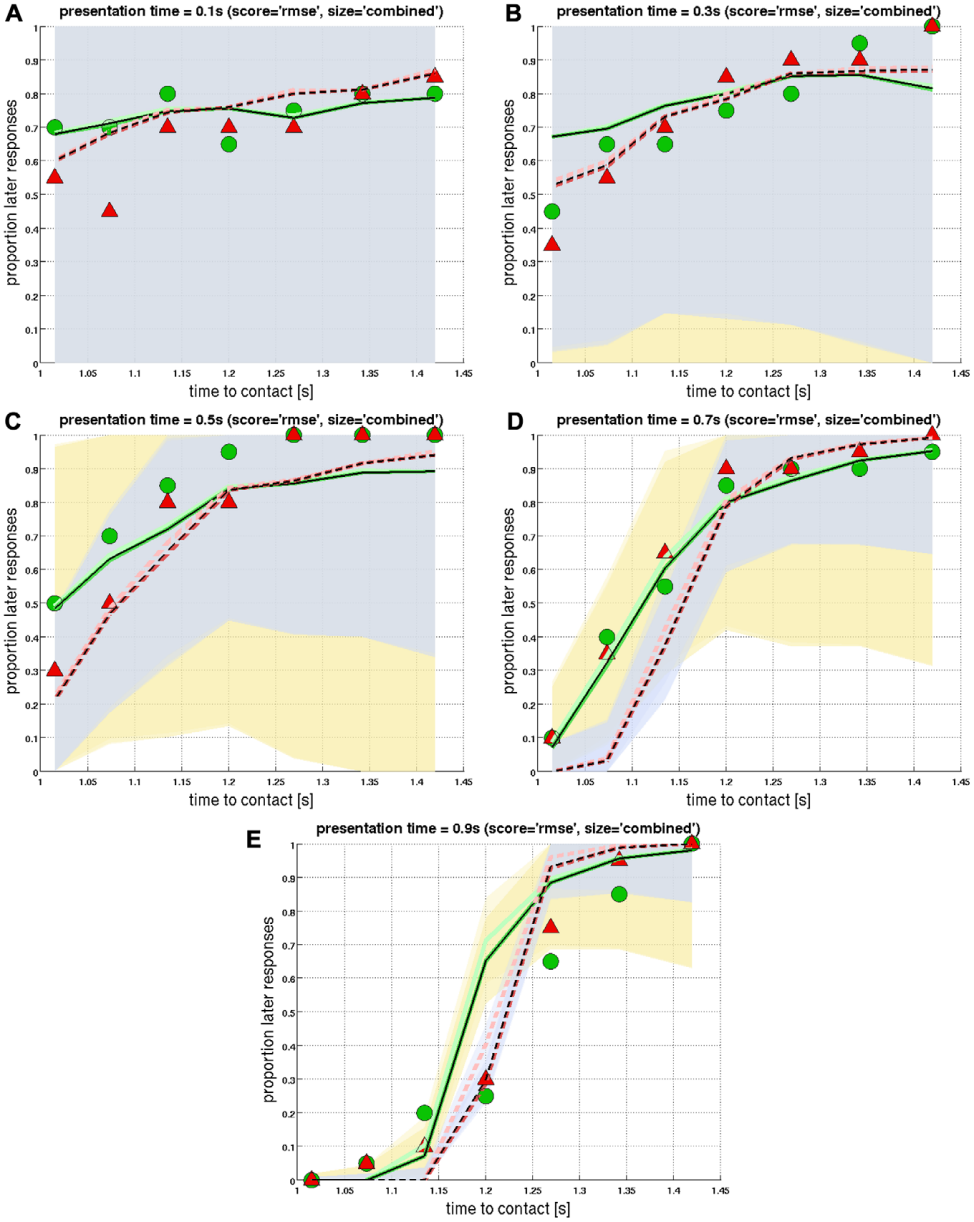
*Corrected m-Tau* predictions (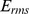 score; combined diameter). The proportion of later responses (i.e. subjects perceived *ttc* after 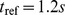) are shown as a function of *ttc* for different presentation times 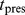: (**a**) 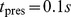, (**b**) 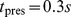, (**c**) 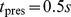, (**d**) 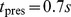, and (**e**) 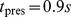. Psychophysical results 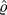 were pooled across subjects and are denoted by circles (*small* object diameter 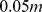) and triangles (*big* object diameter 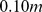), respectively. Predictions 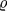 of the *corrected m-Tau* -model “
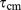
” are represented by curves. In this figure, the prediction performance of 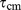 was measured according to the root mean square error (“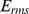-score”). *Corrected m-Tau* -predictions with the three best performing parameter sets are juxtaposed (i.e. first three rows in Table S3 in *Text S5* with smallest 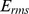-score). Thinner and darker lines represent a better prediction performance. Furthermore, continuous curves are the 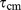-predictions for *small* (thus should match the circles), while dashed curves correspond to *big* (should match the triangles). Here, the same set of 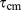-parameters was used for both object diameters (“*combined* diameter”). The light-shaded areas correspond to the variability of simulated responses (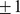 SD, see Methods Section): Yellowish shading for *small*, and bluish shading for *big*.

The full set of data points is shown in Figure 8, where each figure panel corresponds to a different presentation time (*small* object size: circles; *big*: triangles). The curves shown in Figure 8 do not represent GCDF-fits (as in Figure 7*a*), but rather display simulation results from the 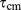-model. For short presentation times, subjects show near-random performance across *ttc* (Figure 8*a, b*), thereby revealing a bias towards later responses (i.e. “ball hit me after 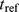
”). The GCDF-fits reveal a higher bias for the *small* object diameter (Figure 7*b*). The corresponding psychometric functions (not shown) and 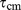-predictions for the shortest presentation time (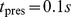; Figure 8*a*) are thus rather flat and noisy. This bias is progressively reduced with increasing 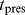, indicating improving performance: For 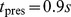, the point of subjective simultaneity approaches 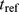 for both object diameters, and psychometric functions get closer to a step-wise increase at 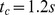 (Figure 7*a*).

We already mentioned that we simulated the psychometric functions with the *corrected m-Tau* -model (equation 5), at which we added noise to angular size and angular velocity (equation 9). By assuming a constant approach velocity, one could compute an estimation of *ttc* with equation (12). Note that this estimation should be constant throughout the approach in a noise-free situation and for “sufficiently small” angular sizes. As a consequence of having noise, however, the *ttc* estimation fluctuates. We therefore computed an average estimation with equation (14), by taking the mean value across a time interval (the interval contained the last 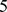 estimates). The average *ttc* estimation was evaluated at presentation time 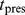, and compared with the reference 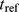. With a total number 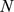 of such trials, we then counted 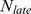 occurrences where the average estimate occurred after 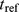. The simulated proportion of later responses is then obtained by dividing 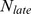 by 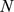 (equation 13).

In order to find the appropriate 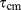-parameters for predicting psychophysical performance, the error between 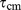-predictions and psychophysical data points was minimized. We refer to this procedure as optimization. Optimization was carried out separately for object diameters *big* and *small*. The first step of the optimization procedure consisted in parsing the parameter space, and recording the error associated with each set of 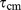-parameters. The error was determined with two measures (“score measures”): The root mean square error (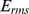), and an outlier-insensitive robust error (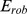). In the second step, the parameter sets were sorted in ascending order with respect to their associated score measure. Sorting took place separately for 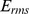 and 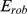, leading to corresponding tables where the best set of parameters was assigned rank one (1st table row), the second best rank two (2nd table row), and so on (Tables S1 & S2 in *Text S5*).

A third table of 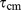-parameters was then computed which was optimal for both object diameters simultaneously (*combined*; Table S3 in *Text S5*). This could be done in a straightforward way, simply by averaging the score measures of *big* and *small* of corresponding parameter sets, and subsequently sorting the averaged errors (more details on finding parameters are given in *Text S5*).

For the computation of 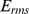 and 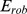, all psychophysical data points that represent the proportion of later responses entered equivalently, in the sense that no weighting coefficients were used to bias the optimization process toward longer presentation times (as GCDF-fits at longer presentation times have a smaller variance, see Figure 7*b*). Notice that parameter optimization for the *combined* diameter naturally implicates a trade off – the errors with respect to *big* and *small* will be bigger compared to individual parameter optimization.

**Figure 9:**
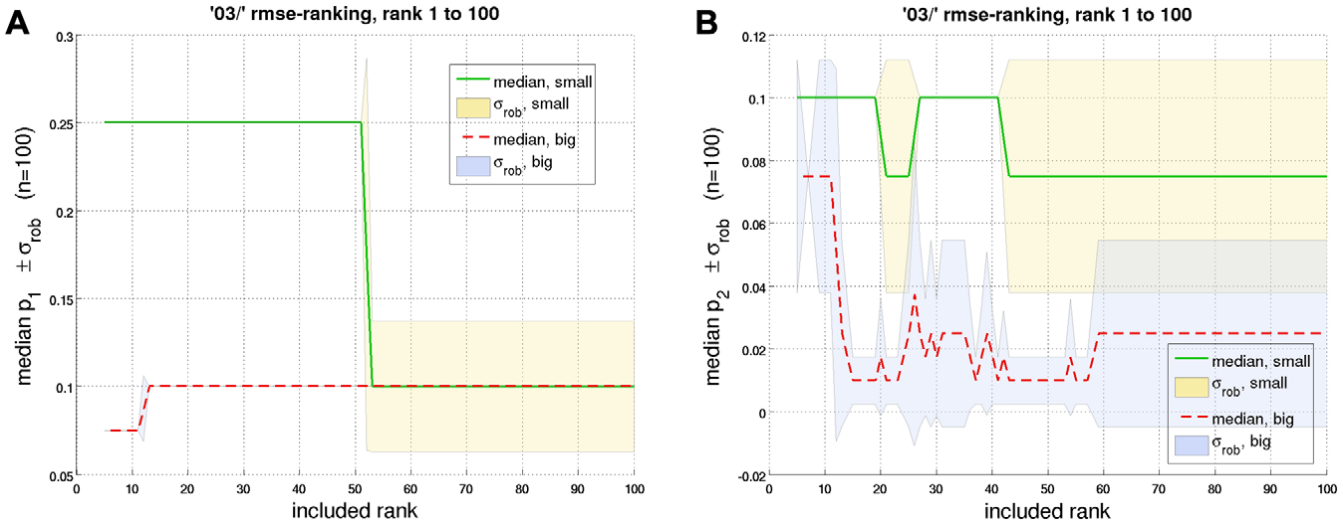
Median value of noise probabilities as a function of 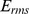-rank. In order to predict psychophysical performance with the *corrected m-Tau* -model, its parameters were optimized. Prediction performance was measured with a score measure, either the root mean square error (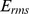, shown here), or an outlier-insensitive robust error (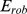; shown in *Text S5*). The 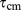-parameter set with which the best prediction was achieved was assigned rank one, the second best rank two, and so on. Thus, rank one corresponds to the parameter set with the smallest score measure. The figure shows the median value of the noise probability equation (9) of: (**a**) angular size 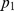, and (**b**) angular velocity 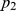, as a function of rank. Abscissa values of 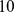, 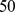, etc. signify that the median value across the first 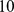, first 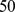, etc. values of 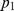 and 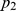, respectively, was computed, according to “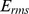-ranking”. Shaded areas indicate 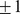 of the corresponding robust estimation of standard deviation 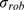. The continuous curves were computed with the 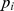-values optimized for the *small* object diameter (listed in Table S1 in *Text S5*), and broken curves denote corresponding values for the *big* diameter (Table S2 in *Text S5*). The curves shown here suggest that the *small* object diameter is associated with a higher noise level. This conclusion is valid for 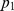 until rank 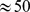 (curves become indistinguishable beyond that value), and for 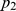 until rank ten: For ranks bigger than ten, 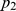 reveals a certain dependence on the score measure and the averaging procedure (not visible in this plot, but see corresponding figures in *Text S5*).

Figure 8 shows that the *corrected m-Tau* -model adjusts fairly well to the psychophysical data of both object diameters. Nevertheless, the 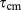-predictions for 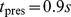 are somewhat worse with the *combined* parameter optimization (Figure 8*e*) when compared to a separate optimization for *big* and *small* (corresponding figures in *Text S7*). The most likely explanation for this discrepancy (individual versus combined parametrizations) is that each object size is associated with a different noise level (noise levels are represented by the 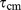-parameters 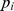 with 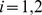; see equation 9). We investigated this hypothesis by comparing the corresponding values of 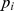 for *big* and *small*, as a function of their rank. Figure 9 shows that the 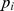 for *small* are consistently higher than for *big*. Therefore, the *corrected m-Tau* -model generally supports the notion that smaller object diameters imply higher noise levels in angular size and angular velocity, respectively.

We also studied two models with less degrees of freedom than *corrected m-Tau* : The first was 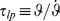, and the second was 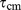 with 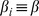 for 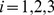 (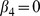). The best (i.e. smallest) score measures achieved with these reduced models were consistently higher than the best values achieved by the *corrected m-Tau* -model (*Text S5*), and their best-ranked parameter sets resulted in psychometric curve predictions that were also inferior by visual inspection (not shown).

## Discussion

With the *corrected m-Tau* -model equation (5), we proposed a general framework that comprises the 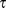-function and several properties of the 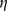-function. By means of adjusting only a single parameter (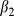), the *corrected m-Tau* -model can approximate 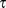 and 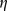, respectively. Moreover, the 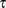-approximation is less sensitive to noise than the original 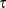-function, and accounts well for the performance of the psychophysical experiment that we carried out.

In the experiment, subjects had to decide whether a (displayed) ball reached them before or after a reference signal at time 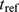. However, balls were only presented until 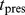, and disappeared afterwards. In other words, subjects had to estimate 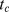 (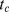 could occur before or after 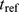). With respect to our experiment, the *corrected m-Tau* -model suggests the following conclusions:

- Subjects relied on a 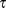-based mechanism for judging *ttc* (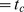). We use the term “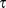-based” as a synonym for any timing-based mechanism. The full *corrected m-Tau* -model better predicted our psychophysical results than any of the two alternative models that we considered (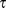 and 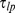).
- The decision about whether perceived 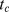 occurs before or after 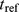 is based on information at (or immediately around) 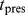, as the only information used was from 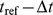 until 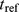 for predicting *ttc* (see equation 14).
- Subjects' performance improves with increasing 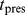, indicating that the signal-to-noise ratio (SNR) that is associated with the computation of (perceived) 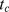 improves during an object approach. Such an improvement can be brought about by two mechanisms. *First*, the noise level is signal-independent and thus stays the same during an object approach. As angular size 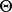 and angular velocity 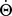 increase monotonically with time, the SNR would improve accordingly. *Second*, noise may increase with the signal [37], but is concurrently suppressed by low-pass filtering. Low-pass filtering may be adaptive, such that it adjusts to signal variability in each moment. We are not aware of any such signal-dependent noise suppression, and we therefore deem the first mechanism to be the more likely. Accordingly, we propose that approaching objects with smaller size lead to decreased SNRs in the signals that represent 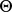 and 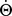, respectively.
- The perception of *ttc* in humans reveals a certain dependence on object size [22]. Thus, one might argue that 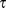-based mechanisms are not an adequate model for *ttc* perception, because they are largely object-size-independent in the early phase of an object approach when 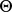 is still “sufficiently small”. However, this argument ignores noise. As long as the noise-induced fluctuations in 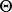 and 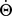 do not cancel (“correlated noise”), the SNR of 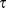 will depend on object size (Figure 6). Therefore, any decision based on computing 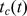 with a 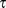-based mechanisms will be limited by the SNR at time 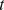 (*ttc* can be computed by adding 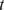 to 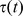, because in the early phase of an approach 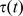 decreases linearly with time for 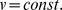, see equation 12). The SNR improves with increasing object size and with decreasing (initial) distance between object and observer. Thus, bigger objects will imply better accuracy in estimating 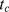. Similarly, smaller distances will imply better estimation accuracy. Both effects are observed in our psychophysical experiment, where a better “estimation accuracy” translates into psychometric curves that adjust better to a step-wise increase from zero to one at 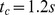 (because of 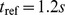; Figure 7*a*). Without noise, however, 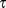-based mechanisms cannot predict such dependence on object size for small angular sizes.

The modified 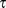-model (“
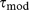
”) constitutes a special case of 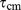. It is obtained from equation (5) for 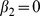 (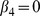 by default). Its distinguishing feature is a maximum before 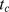, which can be shifted via 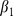 (equation 2). The 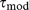-maximum decreases as it is positioned closer to 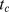, because this implies bigger values of 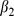. The time 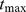 of the 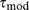-maximum depends furthermore on size and velocity (Figure 1). The curve shape of 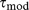 is reminiscent of the 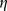-function, since both functions have a maximum. We thus decided to fit previously published response curves from collision sensitive neurons to both functions, and observed that both functions fit the neural curves well in terms of goodness-of-fit criteria (*Text S4*). We must not forget, however, two important differences between 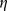 and 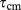.

*First*, since 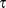 reveals a minimum shortly before 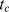 (*Text S6*) and 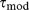 derives from 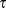, the 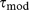-response is more precisely *biphasic*. The biphasic structure gets pronounced in some of the curve fits, especially when 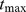 is close to 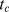 (see corresponding figures in *Text S4*). Then, the amplitude of the 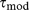-maximum is small, and consequently the fitting algorithm has to scale it to the maximum of the neuronal recording data. In this way, the minimum is also scaled.

*Second*, 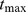 depends in a nonlinear way on the size-to-velocity ratio 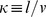 (see Figure 4 for an illustration). This is contradictory to several studies that found a linear dependence. A linear dependence is also predicted by the 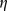-function (equation S5 in *Text S2*). The contradiction can be alleviated by adding noise to relative time of the 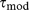-maximum (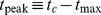; equation S10 in *Text S2*), with noise amplitudes as reported in [26]. As a consequence of noise, the nonlinear relationship can be literally hidden (Figure 3), such that statistical tests would affirm an underlying linear process (*Text S2*). Masking by noise is more effective for bigger values of 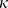, because the noise level is proportional to 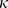.

The 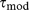-function in its original form cannot explain the neuronal response curves for an approach with 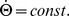 (“linear approach”) [25]: Rather than predicting a decreasing response with time, the 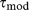-function would linearly increase. In contrast, the 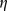-function makes correct predictions. Correct predictions with 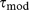 can nevertheless be made by including an additional inhibitory process in the firing rate equation of 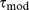 (equation S3 in *Text S1*, where a full proof of concept is described). Important, this extension of 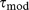 *(i)* is based on a power function with an exponent between 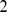 and 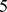, but not on an exponential function as with 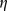, and in this regard it may hence be considered as being biophysically more plausible than 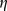 (see also reference [36]); *(ii)* does not interfere with the “normal” 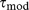 behavior (i.e. normal object approaches are not affected); and *(iii)* tolerates high noise levels (i.e., the mechanism is robust).

What about alternative models which also have a response peak? In *Text S6* we studied two such functions, namely “inverse 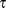
” (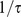), and angular acceleration (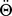). Both of them reveal a linear dependence of 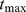 on 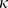 (equations S24 & S26, respectively, in Text S6). The maximum of 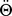 always precedes that of 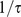. However, 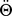 does not make correct predictions for the “linear approach”, as we would obtain 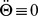 *ab initio* for 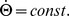 (although a dynamical version may predict the decreasing LGMD-activity on the basis of temporal filtering effects).

In contrast, 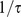 would make consistent predictions in that case. Without further modifications, though, neither 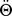 nor 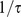 seems to be adequate for fitting the response curves of collision sensitive neurons, because there is no free model parameter to shift their respective maximum. Although the occurrence of their maxima could principally be controlled by a global shift of the time scale 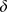, the corresponding values (obtained by fitting the neuronal response curves) would overestimate experimental values (*Text S6*). Similarly, when “fitting” the 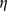-function to 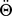 and 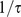 the so obtained values of 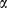 would underestimate experimental values: The 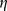-maximum would coincide with the maximum of 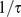 for 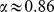, and with the maximum of 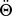 for 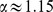.

In conclusion, 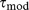 is no replacement for the 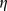-function, at least for describing neuronal responses of collision sensitive neurons in insects. However, in the *nucleus rotundus* of pigeons three classes of neurons were reported [1], [38]. They conform to 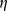-like, 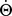-like, and 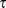-like responses. The fact that 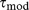 is just a special case of 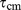 could possibly explain why neurons with 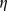-like and 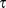-like properties can be found in a single brain. Within the 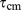-framework, the 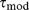 function corresponds to 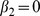, and 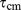 is obtained for choosing 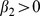. Thus, the adjustment of only a single weight (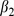) is necessary to go from one function to the other. The *corrected m-Tau* -framework could thus offer a parsimonious yet full-fledged explanation of the implementation of 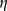-like and 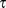-like neurons at the circuit level.

## Methods

### Psychophysical experiment

#### Subjects

Four subjects that were members of the Basic Psychology Department of the University of Barcelona participated in the experiment. All had normal or corrected-to-normal vision and were naive with respect to the aims of the experiment. Two of the subjects were well-trained psychophysical subjects in similar tasks. None of the subjects was stereo blind (StereoFly test, Stereo Optical Co.). They all signed an informed consent. The psychophysical experiment was approved by the Ethics Committee of the Faculty for Psychology of the University of Barcelona, in agreement with the ethical guidelines of the Declaration of Helsinki in 1954.

#### Stimuli and apparatus

Stimuli were displayed on a Phillips 22 inch monitor (Brilliance 202P4) at a refresh rate of 118 Hz and a screen resolution of 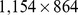 pixels. A 3Dlabs VP870 video card controlled the stereo shutter spectacles (CristalEyes). Simulated targets were uniform disks that moved on a collision trajectory along a line that passed the midpoint between the subjects' eyes. The screen was at one meter distance from subjects' eyes.

Seven time-to-contact values (experimentally fixed values 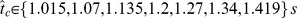) were combined with two different object sizes (diameter 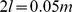 and 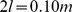), and five presentation times (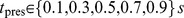), totaling 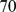 different combinations. In order to ensure that the subjects used the judged time to contact rather than some other correlated measure, we varied the initial simulated starting distances (from 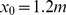 to 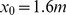), and set velocities 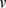 to 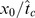.

#### Procedure

Each simulated object appeared at its initial distance 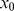 on the monitor. After one second, the object started approaching the observer at the designated constant velocity 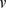, and was visible until 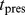 (presentation time). The reference time 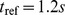 was indicated to subjects with an acoustic signal (beep) [16]. The reference time remained unchanged throughout the experiment. Subjects were instructed to press one of two buttons to indicate whether they thought being hit by the object before or after 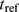. In each session, the complete set of 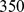 stimuli was shown to subjects in random order (five repetitions times the 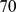 combinations). Each subject took part in five sessions. Feedback on incorrect responses was provided after each trial.

### Simulation of our psychophysical experiment

We simulated our psychophysical experiment with the *corrected m-Tau* -model (equation 3), where we plugged in noisified versions of the optical variables (i.e. 
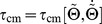
),
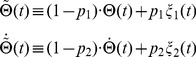
(9)with noise probabilities 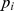 (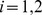), and with the dot denoting the time derivative. The 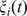 are random variables, which at each instant 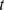 return a value that is drawn from a centered normal distribution. In the last equations, we used the explicit expression for angular size,
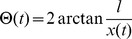
(10)and angular velocity (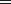
rate of expansion)
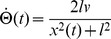
(11)with 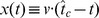 and 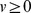. The values of 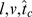 and 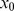 are the psychophysical stimulus parameters. Simulations were carried out with a temporal resolution of 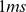.

The *corrected m-Tau* -model is constrained by two limit functions: Ordinary 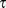 on the one hand (equation 6), and 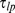 on the other (equation 8). Both limit functions decrease approximately as 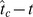 (illustration: Figure 6). Thus, a *ttc* estimation at time 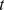 can be computed as
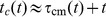
(12)(Nomenclature: 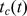 is the model prediction for *ttc* at time 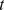, and 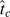 is the experimentally set parameter). In the psychophysical study, subjects were asked to estimate whether they were hit by the approaching object before or after 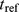. We accordingly define their *proportion of later responses* 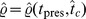 as the number of trials 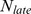 (where subjects responded with being struck after 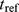) divided by the total number of trials 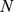:
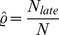
(13)

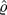
 is represented by circle and triangle symbols in Figure 7 and 8. The corresponding predictions from the model are denoted by 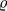. Specifically, 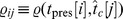 with 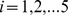 and 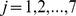, and analogous for 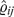. Computation of 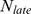 is required for 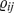, which we did with equation (12) as per
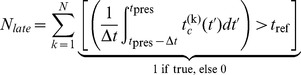
(14)Notice that, due to noise (equation 9), 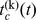 will be subjected to random jitter with each trial 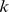. Therefore, in order to obtain a more robust estimate of *ttc* , we do not use only 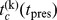: The integral in the last equation computes – in the discrete case – the mean value across the last 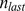 time steps until 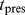 (typically 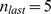, what amounts to a time interval for averaging of 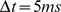, cf. first figure in *Text S7*). In order to illustrate the noise level at each 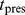, we also computed the standard deviation 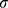 of the 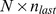 last values of 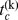. The shaded areas in the figures which visualize 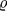 & 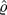 correspond to 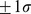. Predictions of the *corrected m-Tau* -model are shown as curves in Figure 8, as well as in *Text S7*.

### Parameters of the *corrected m-Tau* model

The *corrected m-Tau* -model has eight free parameters: 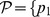, 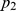, 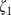, 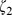, 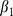, 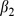, 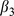, 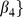. The parameter space was parsed with constant step widths. For each set of parameter values 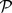, 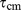-predictions for the proportion-of-later-response curves were computed according to the procedure described in the previous section. The corresponding “goodness of prediction” (or “prediction performance”) was evaluated with the root mean square error (*rmse*, 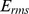), and the outlier insensitive, robust error (*robe*, 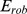), see equation S18 in *Text S5*. The “goodness of prediction” measures are referred to as *score*-measures (*rmse*-scores & *robe*-scores, respectively). Parameter values were sorted according to their scores. In this way we ended up with several score tables, which list the best set of parameters, according to object size: Table S1 in *Text S5* for *small* object diameter (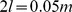), Table S2 in *Text S5* for *big* object diameter (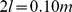), & Table S3 in *Text S5* for *combined* object diameter. The scores for the *combined* size were computed by averaging the scores of *big* & *small* for corresponding parameter values, and then sorting the averaged scores in ascending order. More details on parameter finding and analysis are given in *Text S5*.

### Derivation of Equation 2

Consider a rigid sphere (object radius or half-size 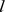) that approaches an observer on a direct collision course. If the approach proceeds at a constant velocity 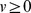, the object-observer distance at time 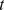 is 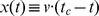. Thus, the initial distance is 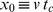.

Now, consider the gain control factor 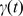 from equation (1)

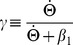
(15)where we plug in the explicit expression for angular velocity equation (11) and obtain
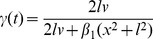
(16)Especially in the initial phase of the approach, when visual angle and angular velocity are sufficiently small, 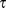 decreases approximately linearly with time (cf. *Text S6*),
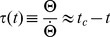
(17)Because of 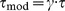, the m-Tau function becomes approximately
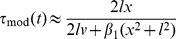
(18)A maximum of the m-Tau function implies that its first time derivative is zero. We define 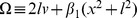. The first time derivative of the (approximate) m-Tau function
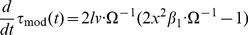
(19)disappears if 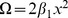, or
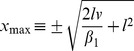
(20)The last equation is the distance 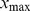 (positive sign) where the approximated m-Tau function attains its maximum during an object approach. Thus, the time 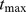 when the 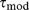-maximum occurs is
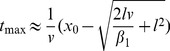
(21)


## Supporting Information

Text S1
**Properties and extension of modified Tau.** *Text S1* presents additional mathematical details of the 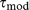-function. Specifically, it is shown how the 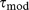-function could be extended to a model which predicts the so-called “linear approach” data. Corresponding simulation results from this model are also shown.(PDF)

Text S2
**Nonlinearity of the m-Tau function.** *Text S2* is dedicated to the nonlinear character of 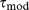 and how it could be successfully hidden behind noise. The section presents additional figures with random trials (analogous to Figure 3*b*), and corresponding scatter plots with goodness-of-fit measures as a function of 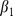.(PDF)

Text S3
**Noise suppression.** *Text S3* considers the numerical robustness of 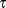, 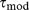, 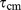 and 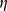, by adding correlated and uncorrelated noise to the angular variables. Similar to Figure 6, it is shown how noise affects these functions (e.g. bigger object diameters are associated with correspondingly less fluctuations), and thus the results presented in this section help to understand the simulation and the interpretation of our psychophysical experiment.(PDF)

Text S4
**Fitting m-Tau and** 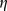 **-function to neuronal recordings.** *Text S4* juxtaposes the individual fitting results of 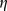 and 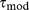 to a variety of previously published neural recording data, which served to compile Figure 2. Further summary results are presented along with fitting results of individual recording traces.(PDF)

Text S5
**Finding parameter values for** ***corrected m-Tau*** **.** *Text S5* describes the optimization procedure for the *corrected m-Tau* -model “
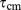
”, with which we obtained the parameter values for the simulation of our psychophysical experiment (e.g. Figure 8). The 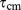-parameters were optimized in three different ways: For achieving a good prediction performance of the psychophysical data corresponding to *(i)* the *small* object diameter (Table S1 in *Text S5*), *(ii)* the *big* object diameter (Table S2 in *Text S5*), and *(iii)* both diameters at the same time (*combined*; Table S3 in *Text S5*). The best ten values are listed in their respective tables according to their psychophysical prediction performance (as quantified by score measures 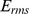 and 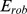, respectively): The best parameter set (smallest score measure) was assigned rank one, the second best rank two, etc. Several figures were compiled that show an additional analysis of the parameter ranking.(PDF)

Text S6
**Time to contact approximation of “Tau” and** 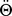 **.** *Text S6* presents a comprehensive analysis of two alternative functions which have a maximum before *ttc*, namely “inverse 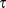
” (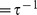) and “angular acceleration” (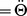). The two functions were also fitted to the neuronal recording data (they turn out to be inadequate), and compared to the maximum of the 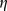-function. This section also provides insights into the biphasic nature of 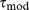, because as 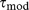 approaches *ttc*, it gets more similar to 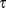, and thus reveals a minimum.(PDF)

Text S7
**Predictions of** ***corrected m-Tau*** **for the psychophysical experiment.** *Text S7* shows the full set of figures with simulation results of our psychophysical experiment. Whereas Figure 8 shows 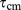-predictions that were obtained with the parameter set optimized for the “*combined*” object diameter according to 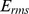-score, *Text S7* shows analogous figures for the remaining parameter optimizations (*big* and *small* object diameter, and 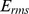 and 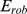-scores, respectively).(PDF)

Text S8
**First order temporal low-pass filter (** **Equation 4** **).** *Text S8* gives a short introduction to the temporal low-pass filter that forms a part of the 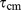-model (equation 4), and is also used for the extension of the 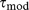-model described in *Text S1*).(PDF)
