# Supplementary material for: Unifying Time to Contact Estimation and Collision Avoidance across Species
Source: PLoS Comput Biol. 2012 Aug 16;8(8):e1002625. doi: 10.1371/journal.pcbi.1002625 (PMC3420976; doi:10.1371/journal.pcbi.1002625)
Supplement: Text S1 — Properties and extension of modified Tau. Text S1 presents additional mathematical details of the -function. Specifically, it is shown how the -function could be extended to a model which predicts the so-called “linear approach” data. Corresponding simulation results from this model are also shown. (PDF) [file pcbi.1002625.s001.pdf]

S1 Properties and Extension of Modified Tau

S1.1 Size and Velocity Effect

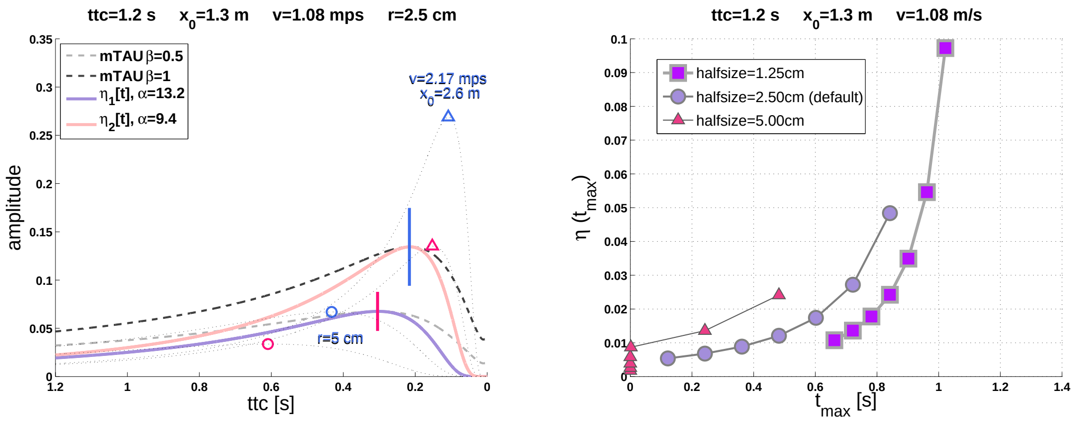

(a) Eta-function and m-Tau (b) Eta-function Maxima)

Figure S1: **The η-function. (a)** Same as Figure 1b, but here two η-functions with $\alpha = 13.2$ and $\alpha = 9.4$ are shown. For comparison, two $\tau_{\rm mod}$-functions with $\beta_1 = 0.5$ and $\beta_1 = 1$, respectively, are also plotted, whose maxima coincide with those of η (amplitudes of $\tau_{\rm mod}$ functions were correspondingly rescaled). Note that both η-functions have a more pronounced decrease after the maximum than the corresponding $\tau_{\rm mod}$-functions: Whereas the η-functions approach zero before t_c, the $\tau_{\rm mod}$-functions do not. The default maxima are marked by vertical bars, and correspond to stimulus parameters $t_c = 1.2\,s$, $x_0 = 1.3\,m$, $v = 1.08\,m/s$, and $l = 2.5\,cm$. Their shift directions (as a result of doubling either object size or velocity) are identical with the m-Tau function (Figure 1): Both maxima of the η-function shift to the left (circles) upon multiplying the object's default halfsize l by two ("size effect"). A shift in the opposite direction (triangles) occurs upon doubling approach velocity v and initial distance x_0 ("velocity effect", $t_c = 1.2s$). **(b)** Same as Figure 1b, but here the size effect is demonstrated for the η-function. Unlike m-Tau, the maxima of the η-function do not lie nor shift on a straight line. Circle symbols represent the default case, with η-function maxima allocated at times $t_{\max} \in \{0.12, 0.24, 0.36, 0.48, 0.60, 0.72, 0.84\}\,s$. The velocity effect is illustrated with Figure S2.

S1.2 Remarks on Equation 1

1. In the m-Tau function $\tau_{\rm mod}(t) \equiv \gamma(t) \cdot \tau(t)$, the factor $\gamma(t)$ provides gain control to $\tau(t)$:

$$\lim_{\dot\Theta \to 0} \gamma(t) = \lim_{\dot\Theta \to 0} \frac{\dot\Theta}{\dot\Theta + \beta_1} = 0 \qquad \text{(S1)}$$
$$\lim_{\dot\Theta \to \infty} \gamma(t) = 1$$

   if $|\beta_1| > 0$ and constant, and thus $\gamma(t)$ is constrained to the interval from zero to one, with asymptotic interval boundaries.

2. The m-Tau function can be interpreted as steady-state solution of the differential equation

$$\frac{d\tau_{\rm mod}(t)}{dt} = -\beta_1 \tau_{\rm mod}(t) - \dot\Theta(t)\tau_{\rm mod}(t) + \Theta(t) \qquad \text{(S2)}$$

   The last equation describes a neuron which encodes $\tau_{\rm mod}$ in its mean firing rate [1]. The decay rate (leakage conductance) is set by β_1, with resting level at zero. The neuron receives silent or

shunting inhibition (i.e. reversal potential equal to the neuron's resting potential) with strength $\dot{\Theta}$. Excitatory input is provided by Θ.

3. In summary, the m-Tau function comprises three desirable properties with one equation: *(i)* it remains finite ("computationally stable") for $\dot{\Theta} = 0$, *(ii)* it can be formally expressed as providing a gain control for the τ-function, and *(iii)* it can be readily cast into a differential equation for neuronal firing rate.

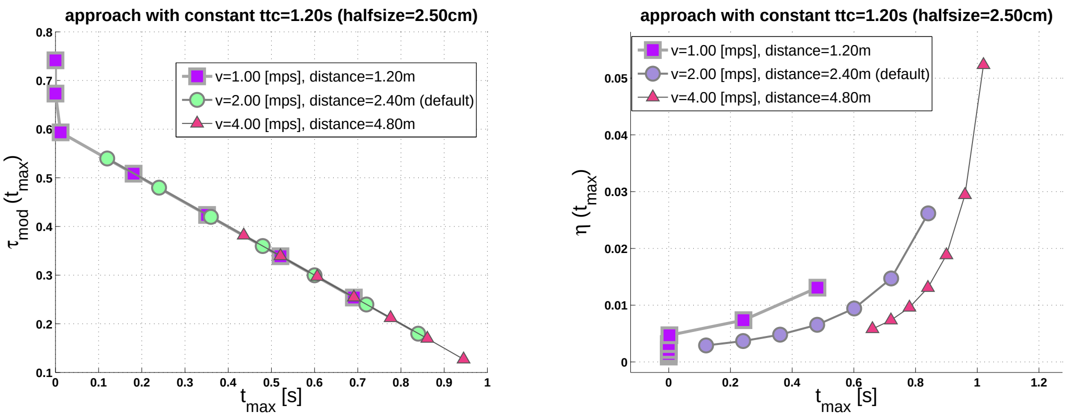

(a) m-Tau (b) η-function

Figure S2: **Velocity effect.** The figure illustrates how the maxima of m-Tau function and η-function behave upon changing the velocity of an approaching object. Notice that, in order to maintain $t_c = 1.2\,s$, the initial object-observer distance had to be modified accordingly (see legend). The rest of the parameters are identical with Figures 1 and S1, respectively, and are indicated at the top of each figure panel. The default values for speed and initial distance were $v = 2.0\,m/s$ and $x_0 = 2.4\,m$, respectively. Maxima corresponding to the default values are indicated by circle symbols. **(a)** Changes in speed translate to shifting the default data points to the left ($v = 1.0\,m/s$) and to the right ($v = 4.0\,m/s$). Similar to the size effect (Figure 1*b*), default and shifted data points lie on a straight line (except for some numerical inaccuracies associated with the two leftmost points). **(b)** Compared to the m-Tau function, variation in speed leads to separates curves for the maxima of the η-function. All curves are furthermore nonlinear, with their amplitudes $\eta(t_{\max})$ increasing when maxima move closer to *ttc*.

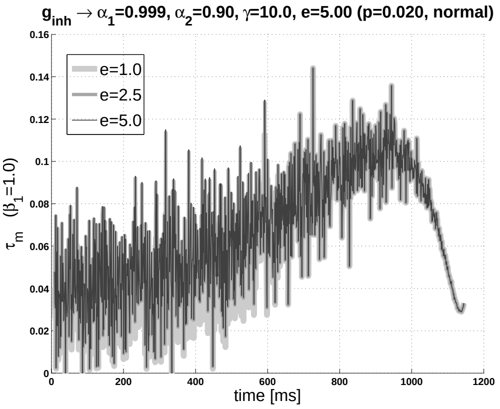

(a) physical (various e)

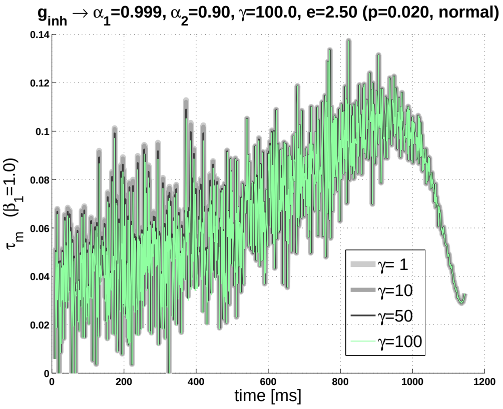

(b) physical (various γ)

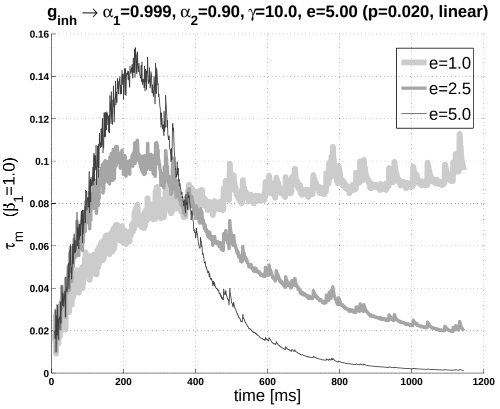

(c) linear (various e)

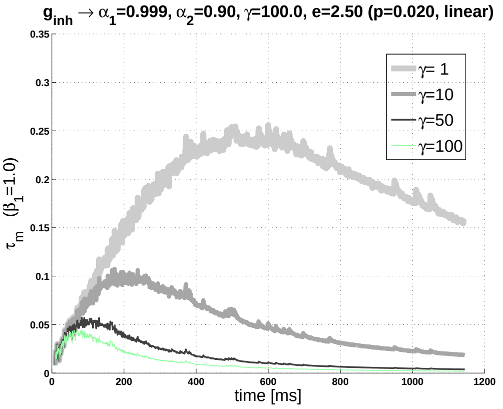

(d) linear (various γ)

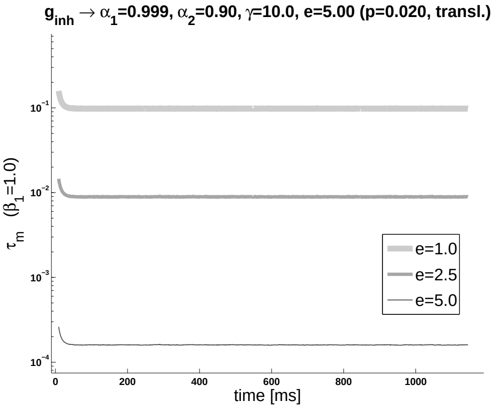

(e) translation (various e)

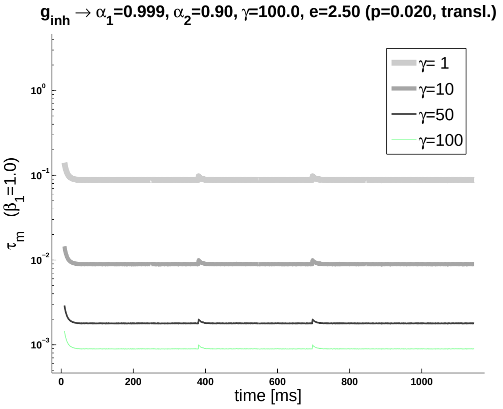

(f) translation (various γ)

Figure S3: **Simulation results I: Modified Tau with additional inhibition.** Simulation of equation (S3) for different types of object approaches, and for different values of e (left figure panels) and γ (right panels) of equation (S4). Default parameters were $\alpha_1 = 0.999$ (memory coefficient for filtering $\dot{\Theta}$), $\alpha_2 = 0.9$ (memory coefficient for low-pass filtering of x), $\gamma = 10$ (constant gain factor), and $e = 2.5$ (power law exponent). Noise was added to angular variables according to equation (9), with $p_1 = p_2 = 0.020$. **(a, b)** "Normal" object approach (approaching speed $1.13m/s$, object half-size $l = 0.025m$, distance $x_0 = 1.3m$). Inihibition stays silent ($g_{\mathrm{inh}}(t) = 0 \forall t$) because $\ddot{\Theta}(t)$ exceeds the threshold value 5×10^{-5} most of the time. **(c, d)** A linear approach (i.e. $\dot{\Theta} = \mathrm{const.}$) triggers inhibition proportional to Θ^e (equation S4), and suppresses τ_{mod}-responses for $e > 1$ after an initial transient. This behavior is consistent with corresponding experimental observations [2]. **(e, f)** Perhaps an ecologically more relevant situation is the suppression of responses to translating objects, or ego-motion as consequence of translation movement (both of which $\dot{\Theta} \approx 0$). Suppression of such responses occurs again after some initial transient.

S1.3 At a glance: The η-Function and the m-Tau Function

	eta-function $\eta(t)$	m-Tau-function $\tau_{\mathrm{mod}}(t)$
**definition**	$A \cdot \dot{\Theta} / \exp(\alpha\,\Theta)$	$\Theta/(\dot{\Theta} + \beta_1)$
**peak location $\mathbf{t}_{\max}$ = ...**	$t_c - \alpha \cdot \kappa$	$t_c - \sqrt{\kappa(\frac{2}{\beta_1} + \kappa)}$
**place peak at $\mathbf{t}_{\max} \rightsquigarrow$**	$\alpha = (t_c - t_{\max})/\kappa$	$\beta_1 = 2\left[(t_c - t_{\max})^2/\kappa - \kappa\right]^{-1}$
**shift of maximum**	$\alpha^{(1)} > \alpha^{(2)} \rightsquigarrow t^{(1)}_{\max}$ before $t^{(2)}_{\max}$	${\beta_1}^{(1)} > {\beta_1}^{(2)} \rightsquigarrow t^{(1)}_{\max}$ after $t^{(2)}_{\max}$
**inhibitory input**	$\exp(\alpha \cdot \Theta)$	angular velocity $\dot{\Theta}$
**firing rate equation**	not straightforward (cf. [3])	$\frac{d\tau_{\mathrm{mod}}(t)}{dt} = -\tau_{\mathrm{mod}}(\beta_1 + \dot{\Theta}) + \Theta$
**stability issues**	no	none for $\beta_1 > 0$
**direct relation to t_c**	no	$\tau_{\mathrm{mod}}(t) = \gamma(t) \cdot \tau(t)$
**lower parameter limit**	$\lim_{\alpha\to 0} \eta(t) = \dot{\Theta}$	$\lim_{\beta_1\to 0} \tau_{\mathrm{mod}}(t) = \tau(t)$
**upper parameter limit**	$\lim_{\alpha\to\infty} \eta(t) = 0$	$\lim_{\beta_1\to\infty} \tau_{\mathrm{mod}}(t) = 0$

Additional information: $\kappa \equiv \mathbf{l/v}$ is the ratio of object radius ("half-size") to object velocity; $\gamma(\mathbf{t}) \equiv \dot{\boldsymbol{\Theta}}/(\dot{\boldsymbol{\Theta}} + \beta_1)$ is a gain control factor; and $\tau \equiv \boldsymbol{\Theta}/\dot{\boldsymbol{\Theta}}$ is the τ-function. "**Place peak at $\mathbf{t}_{\max}$**" means that η and τ_{mod} adopt their respective maxima at $t_{\max}$ if α and β_1 are calculated with the formulas as shown in the table.

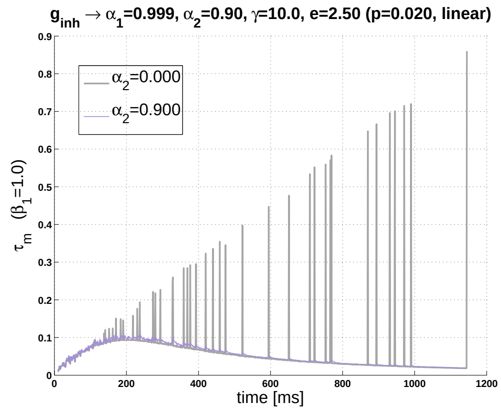

(a)

Figure S4: **Simulation results II: Modified Tau with additional inhibition.** Inhibition $g_{\mathrm{inh}} = g_{\mathrm{inh}}(x,t)$ is assumed to be a low-pass filtered version of x (equation S4). The degree of low-pass filtering is specified by the memory coefficient α_2. Without noise, we could in principle directly use x as inhibitory conductance (i.e. $\alpha_2 = 0$). In the presence of sufficiently high noise levels, though, x would get zero at random times. This could lead to random drop-outs of inhibition in $\tau_{\mathrm{mod}}(t)$, what is indicated by the "spikes" in the figure (legend: curve for $\alpha_2 = 0$). Low-pass filtering of x with $\alpha_2 > 0$ converts g_{inh} into a sluggish process, which bridges the gaps where x is zero (curve for $\alpha_2 = 0.9$).

S1.4 Shut Down of m-Tau Responses for $\dot{\Theta} = const.$

This section is thought as a proof of two concepts: *First*, the m-Tau function can be easily extended to accept further excitatory or inhibitory inputs. Important, these inputs can be incorporated in a biophys-

ically plausible way [1]. *Second*, m-Tau as it stands ("vanilla" τ_{mod}) cannot reproduce the experimental data with constant angular velocity from reference [2]. Situations with $\dot{\Theta} = const.$ may occur if self-motion creates a translatory flow field across the retina, or if any object crosses a visual scene rather than approaching the observer on a collision course. In order to shut down m-Tau responses to such linear object "approaches", we will define a corresponding inhibitory process. We start by adding an inhibitory conductance g_{inh} to the differential equation (S2):

$$\frac{d\tau_{\mathrm{mod}}}{dt} = -\beta_1 \tau_{\mathrm{mod}} - \dot{\Theta}\tau_{\mathrm{mod}} + g_{\mathrm{inh}}[V_{\mathrm{inh}} - \tau_{\mathrm{mod}}] + \Theta \tag{S3}$$

Without loss of generality, we assume $V_{\mathrm{inh}} = 0$ for the inhibitory reversal potential. For the sake of clarity, we omitted biophysical constants for transforming the terms to units of voltage (the state variable τ_{mod} represents voltage). Our goal is to inhibit m-Tau responses for translation movement or ego-motion. To a first approximation, both of the latter movement patterns will have $\dot{\Theta} = const.$, and thus $\ddot{\Theta} = 0$. The idea is to engage inhibition in the latter case, while it should stay silent during any "normal" object approach. To this end we define a *gating process* $\mathcal{G} = \mathcal{G}(\ddot{\Theta}) \in [0, 1]$, with $\lim \mathcal{G}_{|\ddot{\Theta}| \to 0} = 1$, and 0 otherwise. An explicit implementation of $\mathcal{G}$ could be defined via a Heaviside or sigmoid function, respectively. For the simulations shown in figure S3, $\mathcal{G} = 1$ if $|\dot{\vartheta}(t + \Delta t) - \dot{\vartheta}(t)| < 5 \cdot 10^{-5}$, (low-pass filtering analogous to equation 4). Strong low-pass filtering of angular velocity (here with filter memory coefficient $\alpha_1 = 0.999$) increases the resilience of the gating process even in the presence of high noise levels.
Inhibition is furthermore assumed to be a nonlinear function of $x = x(t)$,

$$x = \gamma \Theta^e \cdot \mathcal{G}(\ddot{\Theta}) \tag{S4}$$

with exponent $e = 2.5$ (further values: Figure S3*a*,*c*,*e*) and (here constant!) gain $\gamma = 10$ (further values: Figure S3*b*,*d*,*f*). Finally, the inhibitory conductance $g_{\mathrm{inh}} = g_{\mathrm{inh}}(x, t)$ of equation (S3) is just a low-pass filtered version of x, where we used a memory coefficient $\alpha_2 = 0.9$. Without noise, one could relinquish filtering (i.e. $\alpha_2 = 0$), and directly use x. However, in the presence of noise, inhibition would then randomly switch-off. These drop outs would cause corresponding "spikes" for the linear approach (Figure S4). Note that, unlike the η-function, we did not use an exponential function in equation (S4). A "moderate" power law with $e = 2.5$ is sufficient to get the job done (see also reference [4]).
In figures S3 and S4 noise was added to optical variables, according to equation (9). This means that optical variables Θ and $\dot{\Theta}$ were replaced by $\tilde{\Theta}$ and $\dot{\tilde{\Theta}}$, respectively, in all equations within this section.

References

1. Koch C (1999) Biophysics of computation: information processing in single neurons. New York: Oxford University Press.

2. Hatsopoulos N, Gabbiani F, Laurent G (1995) Elementary computation of object approach by a wide-field visual neuron. Science 270: 1000-1003.

3. Gabbiani F, Krapp H, Koch C, Laurent G (2002) Multiplicative computation in a visual neuron sensitive to looming. Nature 420: 320-324.

4. Keil M (2011) Emergence of multiplication in a biophysical model of a wide-field visual neuron for computing object approaches: Dynamics, peaks, & fits. Neural Information Processing Systems (NIPS) foundation. URL http://books.nips.cc. http://arxiv.org/abs/1110.0433.
