# Supplementary material for: Unifying Time to Contact Estimation and Collision Avoidance across Species
Source: PLoS Comput Biol. 2012 Aug 16;8(8):e1002625. doi: 10.1371/journal.pcbi.1002625 (PMC3420976; doi:10.1371/journal.pcbi.1002625)
Supplement: Text S2 — Nonlinearity of the m-Tau function. Text S2 is dedicated to the nonlinear character of and how it could be successfully hidden behind noise. The section presents additional figures with random trials (analogous to Figure 3b ), and corresponding scatter plots with goodness-of-fit measures as a function of . (PDF) [file pcbi.1002625.s002.pdf]

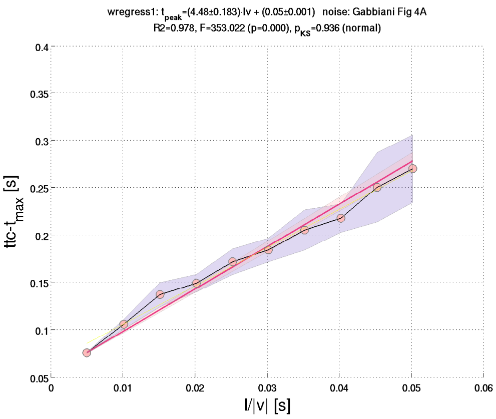

(a)

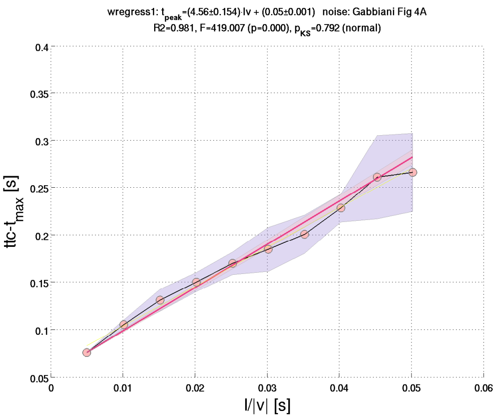

(b)

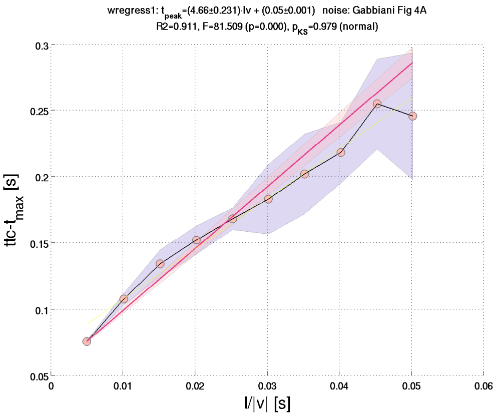

(c)

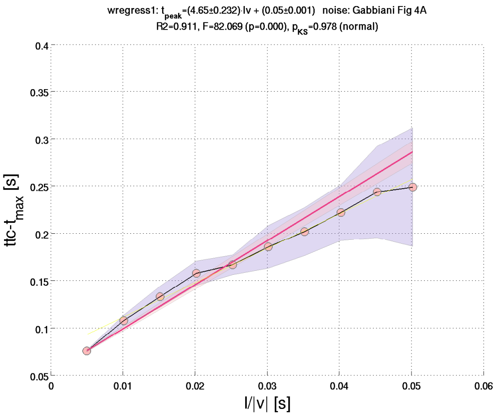

(d)

Figure S5: **Masking of nonlinearity by noise I.** Each panel shows an example of fitting a straight line (with weighted least square regression) to 15 averaged random trials of equation (S10). In this figure $\beta_1 = 1.73464$, and noise levels were set to match those in Figure 3*a* (shaded area). The purpose of this simulation is illustrate how noise can hide the nonlinearity of the m-Tau function. The statistical parameters associated with the weighted linear regression fit are indicated in the headline of each panel.

S2 Nonlinearity of the m-Tau Function

In this section we address whether τ_{mod} can reproduce some of the reported properties of extracellular recordings from locusts' DCMD. This section will focus on the experimental data from *Gabbiani et al.* [1]. We manually resampled their data points (plus standard deviations) from their Figure 4a on page 1128 with an *ad hoc* programmed graphical interface (resampled data are shown in Figure 3*a* & 4, respectively, and a screenshot of the graphical interface is shown in Figure S16*a*). Their data suggest a linear relationship between relative time of peak firing rate and the half-size to velocity ratio $\kappa \equiv l/v$:

$$t_{\mathrm{peak}}^{(\eta)} = \alpha\kappa + \delta \qquad \text{(S5)}$$

where *relative* peak time will be denoted by $t_{\mathrm{peak}} \equiv t_c - t_{\mathrm{max}}$. Notice that *(i)* t_{max} is the absolute time of the maximum, *(ii)* t_{peak} is always positive or zero. *Gabbiani et al.* [1] found slope $\alpha = 4.7 \pm 0.3$ and

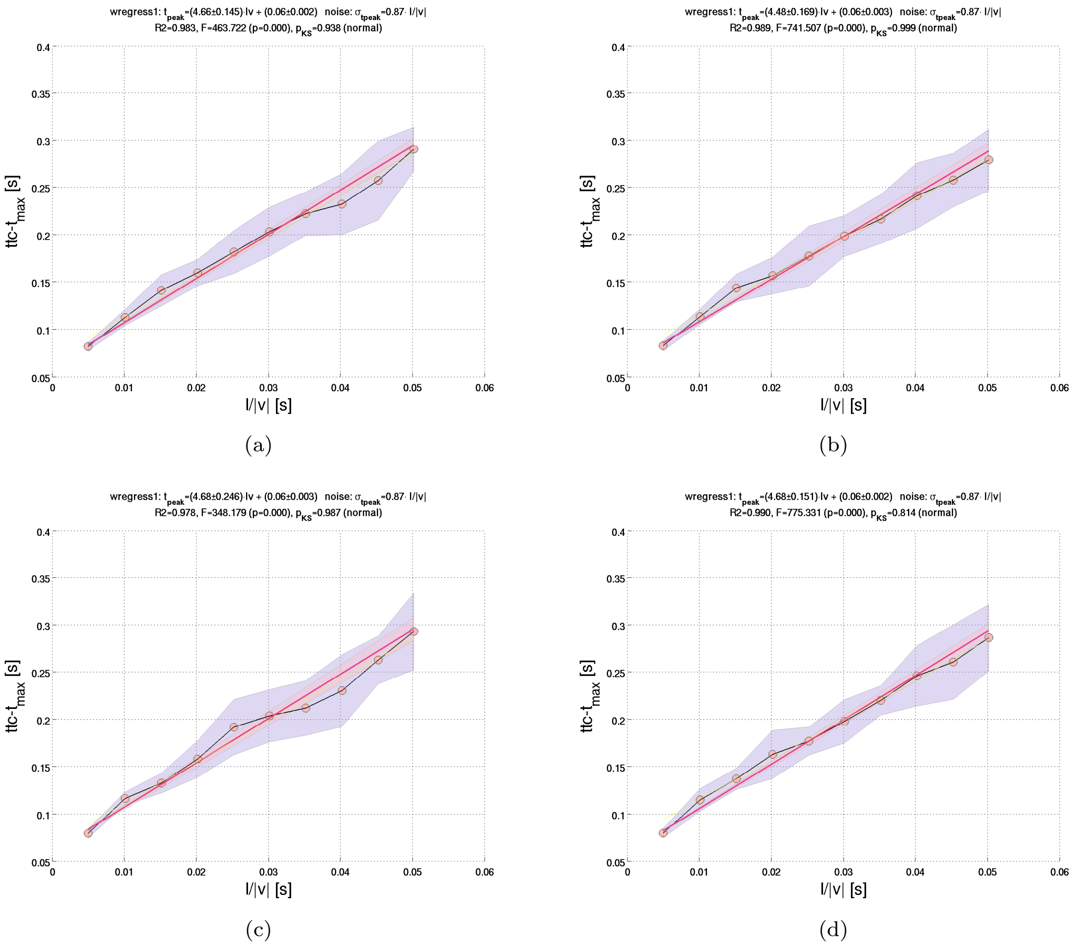

Figure S6: **Masking of nonlinearity by noise II.** Same as the last figure, but here $\beta_1 = 1.52651$, and the noise level in equation (S10) was set to $\xi(\kappa) = 0.87\kappa$. The latter equation was proposed by *Gabbiani et al.* (equation 8, page 1129 in reference [1]). Figure panel d is identical with Figure 3b.

intercept $\delta = -27 \pm 3\,ms$ (± 1 SD, $N = 15$ neurons). We used weighted linear regression for fitting their data and obtained $\alpha = 4.61 \pm 0.179$ and $\delta = -30 \pm 1\,ms$, respectively (figure 3a).
Figure 4 illustrates how the *relative* peak time t_{peak} of the τ_{mod}-maxima depends on κ:

$$t_{\text{peak}} = \sqrt{\kappa \left(\frac{2}{\beta_1} + \kappa \right)} \tag{S6}$$

In Figure 4, the values of β_1 were chosen according to

$$\beta_1 = 2 \left[t_{\text{peak}}^2 / \kappa - \kappa \right]^{-1} \tag{S7}$$

with values for $(\kappa, t_{\text{peak}})$ taken at selected data points marked by red square symbols. Within the displayed domain of κ and range t_{peak}, the curve $t_{\text{peak}}(\kappa)$ appears almost linear for $\beta_1 = 111.70$, but is visibly bended for $\beta_1 = 1.85$. The slope of $t_{\text{peak}}(\kappa)$ can be obtained by linearization of equation (S6) around some constant κ_0 (Taylor series up to the first order):

$$t_{\text{peak}}(\kappa) \approx t_{\text{peak}}(\kappa_0) + (\kappa - \kappa_0) \cdot \left. \frac{\partial t_{\text{peak}}}{\partial \kappa} \right|_{\kappa = \kappa_0} \tag{S8}$$

The last equation represents a straight line (tangent at κ_0) with slope

$$\frac{\partial t_{\text{peak}}}{\partial \kappa} = \frac{\kappa + 1/\beta_1}{\sqrt{\kappa \left(\frac{2}{\beta_1} + \kappa \right)}} \tag{S9}$$

which needs to be evaluated at $\kappa \equiv \kappa_0$. The slope approaches 1 for large β_1, because the terms with $1/\beta_1 \approx 0$. Because then the slope does not dependent on κ anymore, t_{peak} equals a straight line in the

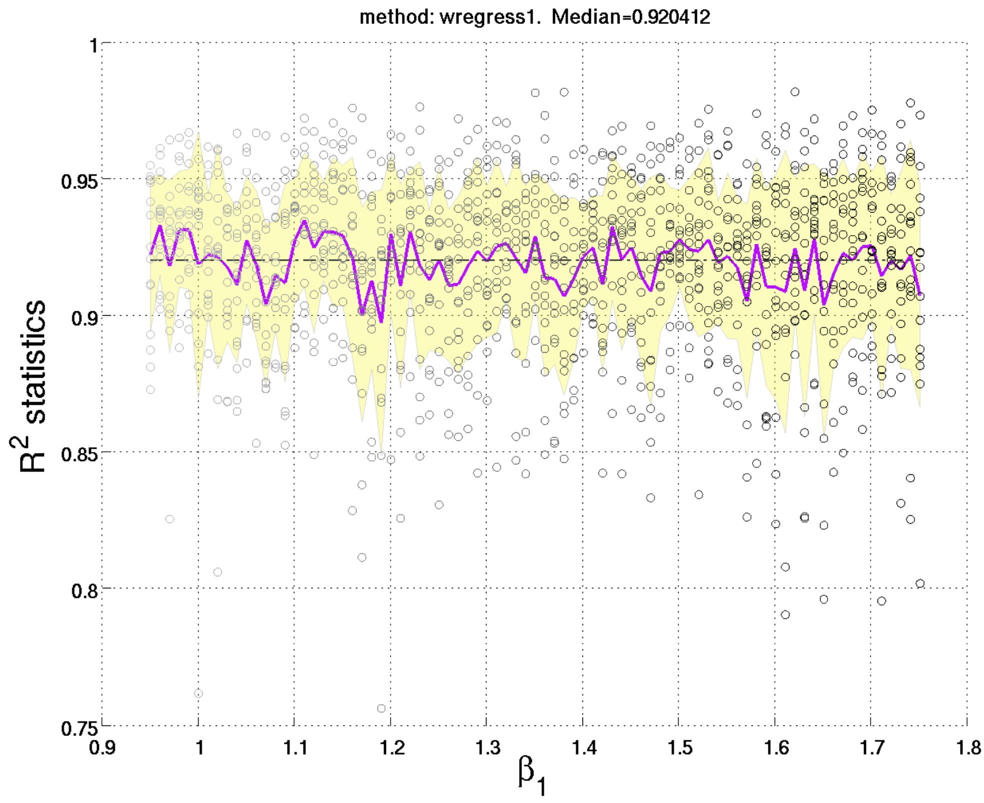

Figure S7: **Statistics of Line Fit I.** The coefficient of determination ($\mathbf{R}^2$) is the proportion of variance of the data which can be explained by the (here linear) model. The continuous line is the mean of the $n = 15$ data points at each of 81 values of β_1. The shaded area indicates one standard deviation. The broken line is the median value (0.92) across all data points.

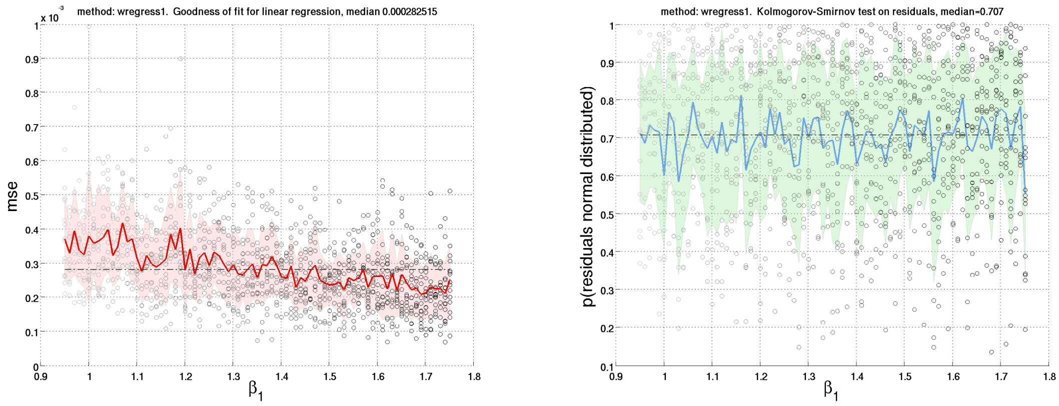

(a) Mean Square Error

(b) Kolmogorov-Smirnov Test

Figure S8: **Statistics of Line Fit II.** See the legend of Figure 5 for computation details. Continuous lines indicate the mean of the $n = 15$ data points at each value of β_1. Shaded areas indicate one standard deviation, and the broken line is the median across all data points. **(a)** Mean square error (MSE) of linear regression improves with increasing β_1. This is what would be expected from Figure 4, because linearity is better approximated with higher values of β_1. The median value across all 81×15 data points is $2.83 \cdot 10^{-4}$. **(b)** Kolmogorov-Smirnov test on residuals gives the probability that the residuals were drawn from a standard Gaussian probability distribution (median across all 1215 values: 0.71). Note that we assumed additive Gaussian noise which linearly increased with the halfsize to velocity ratio κ.

limit $\beta_1 \to \infty$.

If lines are fitted "brute-force" to the nonlinear curves $t_{\text{peak}}(\kappa)$ for finite values of β_1, then both their slopes α and their intercepts δ will increase with decreasing values of β_1 (see corresponding values in

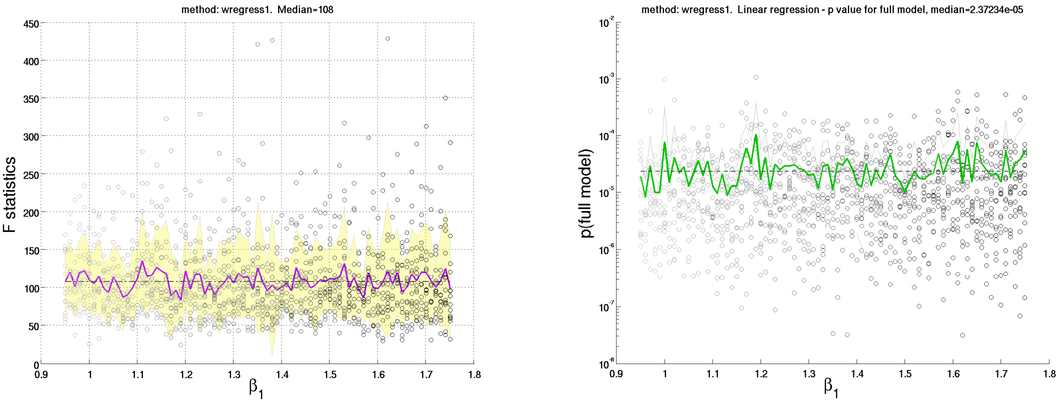

(a) F Statistics

(b) p(full model)

Figure S9: **Statistics of Line Fit III.** Details are described in the legend of Figure 5. Continuous lines = mean of data points ($n = 15$) at each β_1 (81 values). Shaded areas = one standard deviation. Broken line = median across all data points. **(a)** Ratio of the model mean square to error mean square ("F-statistics", median=108). **(b)** Probability of obtaining observed F-values if a reduced model is used instead of the full linear model (median p value $2.37 \cdot 10^{-5}$). Note the logarithmic scaling of the ordinate.

Figure 4 written in small font size under the values of β_1). This hints at a positive correlation between α and δ, that was also reported by *Gabbiani et al.* [1].

The nonlinear dependence of t_{peak} on κ is nevertheless not in agreement with *Gabbiani et al.*'s findings [1], because they reported a linear relationship (equation S5). We observed, however, that sufficiently high noise levels can contribute to hiding the nonlinearity. Our point is illustrated with figures S5 and S6, where each figure panel represents a simulation in which Gaussian noise ξ with zero mean and with standard deviation $\tilde{\sigma}$ has been added to equation (S6). Accordingly, the simulation protocol is defined by *(i)* substitution of equation (S6) by

$$t_{\mathrm{peak}} = \sqrt{\kappa\left(\frac{2}{\beta_1} + \kappa\right)} + \tilde{\sigma}\xi \tag{S10}$$

(ii) averaging 15 results of the "noisified" version of t_{peak}, and *(iii)* fitting a line to the averaged $(\kappa, t_{\mathrm{peak}})$ data. For the simulation we need to specify β_1 and the noise level $\tilde{\sigma} = \tilde{\sigma}(\kappa)$ in the last equation. For the noise level, two settings were considered.

First, $\beta_1 = 1.73464 \pm 0.001516$ (median $\pm\sigma_{rob}$) for the simulations shown in Figure S5, and we measured the standard deviation $\tilde{\sigma}$ at each κ directly from Figure 3*a* (shaded area). *Second*, $\beta_1 = 1.52651 \pm 0.002068$ in Figure S6, and noise levels were assumed to be linearly increasing with κ, according to $\tilde{\sigma} = 0.87\kappa$ (equation 8 on page 1129 in reference [1]).

The specific values of β_1 for the two noise levels were determined numerically with a "stochastic optimization algorithm", which works as follows. First, a candidate value of β_1 is chosen, and $N = 15$ noisified instances of equation (S10) were averaged. In this way we obtained a mean t_{peak} as a function of κ, and associated standard deviations. The inverse of the squared standard deviations were used as weights for a weighted linear regression fit. From the fit a slope value was obtained, which was accepted if $R^2 > 0.9$, $F > 50$, and KS-test $p > 0.5$ were simultaneously fulfilled (acceptance with $p = 0.25$ for the first setting, and $p = 0.67$, respectively, for the second setting). Otherwise the slope value was ignored. All accepted slope values were subsequently averaged (typically 5000), and compared with the target slope $\alpha = 4.7$ from *Gabbiani et al.* [1]. The value of β_1 was increased or decreased as a result of the comparison, and the algorithm continued to modify β_1 until a convergence criterion was met. The algorithm was run several times, and the median of the resulting values of β_1 was taken.

In all simulations shown in Figures S5 and S6, the (simulated) data points are consistent with linearity. The statistical parameters of each weighted regression result (as indicated at the top of each figure panel) are consistent with a linear model, too. Thus, our nonlinear model for t_{peak} (equation S6) can appear as being linear in the presence of suitable noise levels.

Gabbiani et al. noted a positive correlation between α and δ (correlation coefficient 0.76, cf. Figure 4b, page 1128 in reference [1]). Figure 4 suggests such a correlation for lines fitted to equation (S6). We studied this issue in more detail by the procedure described in the legend of Figure 5. For each of 81 values of $\beta_1 \in [0.9, 1.8]$, $n = 15$ data points were generated. Each data point in turn represents inter-

cept δ and slope α of a line fit to $N = 15$ averaged random trials of the "noisified" equation (S6) (i.e., equation S10). Figure 5 shows a correlation between intercept and slope. The addition of noise decreases correlation somewhat, because the long main axis of the ellipse in Figure 5 has a smaller slope than the straight line that characterizes the noise-free situation.

The statistical parameters (Figures S7 to S9) for the 15×81 linear regressions provide further support to that the nonlinearity is successfully hidden by the noise. Specifically, the mean square error (MSE, Figure S8a) decreases with increasing β_1. This behavior is in line with equations (S8) and (S9), respectively, where we noted that $t_{\text{peak}}(\kappa)$ eventually approaches a straight line for increasing values of β_1.

If we ignored the standard deviations associated with the data points $t_{\text{peak}}(\kappa)$, we could use ordinary linear regression or a robust regression method to fit the data. In that case, we would obtain somewhat higher slope values for all fits. For example, ordinary linear regression of the data from *Gabbiani et al.* [1] would yield $\alpha = 5.19$ and $\delta = -40\,ms$. With $\beta_1 = 0.819273$, the corresponding linear regression fits of equation (S10) would again result in consistent predictions for the two considered noise settings. Thus, our argument that the m-Tau nonlinearity could be hidden by noise does not depend critically on the choice of the fitting method.

References

1. Gabbiani F, Krapp H, Laurent G (1999) Computation of object approach by a wide-field, motion-sensitive neuron. J Neurosci 19: 1122-1141.
