# Supplementary material for: Unifying Time to Contact Estimation and Collision Avoidance across Species
Source: PLoS Comput Biol. 2012 Aug 16;8(8):e1002625. doi: 10.1371/journal.pcbi.1002625 (PMC3420976; doi:10.1371/journal.pcbi.1002625)
Supplement: Text S3 — Noise suppression. Text S3 considers the numerical robustness of , , and , by adding correlated and uncorrelated noise to the angular variables. Similar to Figure 6, it is shown how noise affects these functions (e.g. bigger object diameters are associated with correspondingly less fluctuations), and thus the results presented in this section help to understand the simulation and the interpretation of our psychophysical experiment. (PDF) [file pcbi.1002625.s003.pdf]

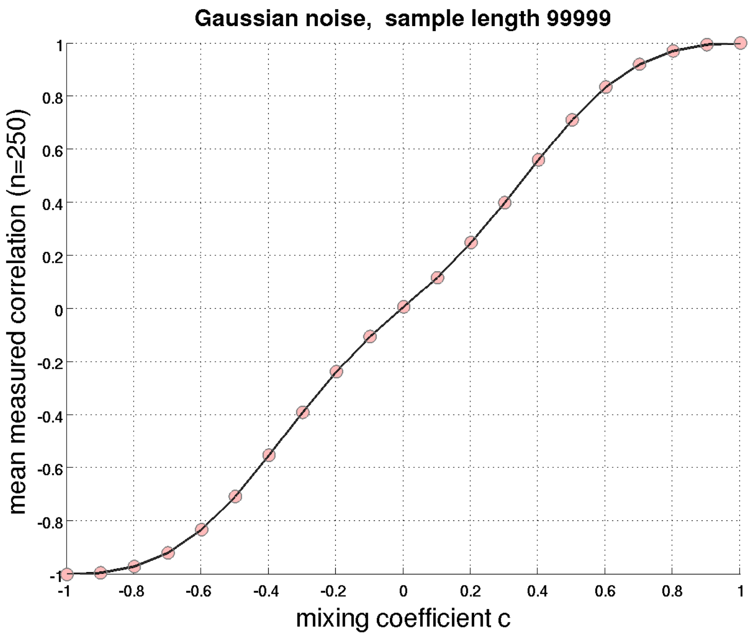

Figure S10: **Mixing coefficient c versus measured correlation.** The figure shows the relationship of the mixing coefficient c in equation (S12) with measured correlation. For this figure, the sample size of ξ_i ($i = 0, 1, 2$) was 99999. Correlation was measured 250 times with different samples, and the mean value is shown. Standard deviations were in the order of $10^{-15}$and thus are not visible. The mixing coefficient c underestimates the measured correlations somewhat for positive values (e.g. $c = 0.6$ yields a measured correlation of about 0.8). Overestimation occurs for $c < 0$.

S3 Noise Suppression

In this section the robustness against noise of the various models will be studied. Such a numerical analysis may seem strange at first sight, because these models do not represent full image processing approaches for *ttc*-estimation (e.g. [1, 2]). We have instead two different objectives in mind: *First*, we want to know how our proposed models τ_{mod} and τ_{cm}, respectively, compare to τ and η. We think that the new model(s) should have at least a comparable noise suppression performance as the previous one(s). *Second*, we need to now how noise affects these functions during an object approach, and how the signal-to-noise ratio depends on certain stimulus parameters. We will require these results for interpreting the simulation-results of our psychophysical experiment, as noise plays an important role in these simulations. We commence with adding Gaussian distributed noise ξ_i (both $i = 1, 2$ mean zero and standard deviation one) with *probability* $p = 0.0075$ to angular variables:

$$\tilde{\Theta} \equiv (1-p)\cdot\Theta + p\xi_1$$
$$\dot{\tilde{\Theta}} \equiv (1-p)\cdot\dot{\Theta} + p\xi_2 \qquad \text{(S11)}$$

In order to examine the impact of correlations between ξ_1 and ξ_2 on model performance, let ξ_0 be a third standard normal distributed random variable (mean zero and standard deviation one), which is uncorrelated with ξ_1. We then define ξ_2 as

$$\xi_2 = c\xi_1 + (1-|c|)\cdot\xi_0 \qquad \text{(S12)}$$

such that the two random variables ξ_1 and ξ_2 have the approximate correlation c, with $-1 \leq c(\xi_1, \xi_2) \leq 1$ (Figure S10).

Figures S12 and S14, respectively, display the relative error of several functions. Let $f(t) \geq 0$ be any function of optical variables (representing, for example τ or τ_{mod}), and $\tilde{f}_i(t)$ the same function with noise at random trial i (equation S11). Then we define the relative error $e_i\left[f(t)\right]$ as

$$e_i\left[f(t)\right] = \frac{|f(t) - \tilde{f}_i(t)|}{f(t) + \epsilon} \tag{S13}$$

with a small constant $\epsilon = 10^{-3}$ to prevent numerical issues. In all figures of this section, the median value $\mathrm{median}_{i\in\{1\ldots999\}}\, e_i\left[f(t)\right]$ across 999 random trials is depicted. In Figures S13 and S15, the median value is integrated, in order to achieve a more compact graphical representation:

$$E(f) = \frac{1}{t_c}\int_0^{t_c} \underset{i\in\{1\ldots999\}}{\mathrm{median}}\; e_i\left[f(t)\right] dt \tag{S14}$$

S3.1 Modified Tau (m-Tau)

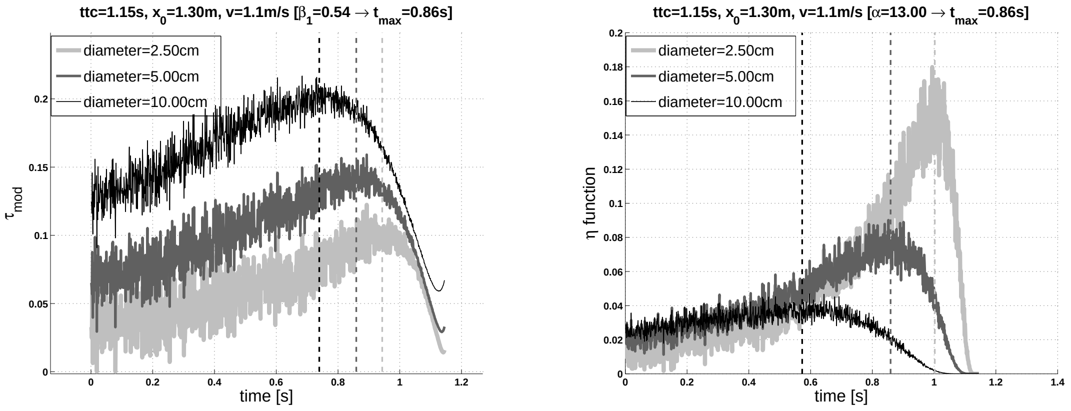

(a) τ_{mod} (b) η-function

Figure S11: **m-Tau and η-function with uncorrelated noise ($c = 0$).** Each curve shows one typical random trial, and the noise was identical for all six curves. The time when both functions had their respective maximum was chosen identically as well ($t_{\max} = 0.75 t_c = 0.86\,s$ at default object diameter $5\,cm$). All theoretical maxima are indicated by broken vertical lines, which have the same luminance as the corresponding curves. **(a)** Because the values of angular variables increase nearly exponentially, whereas their associated noise level stays fixed (i.e., signal-independent, cf. equation S11), less noise fluctuations are seen with $\tau_{\mathrm{mod}}(t)$ as ttc is approached. **(b)** Noise fluctuations also decrease with time for the η-function. Notice that signal amplitudes of the η-function increase with decreasing diameters, whereas signal amplitude is proportional to diameter for τ_{mod}.

The η-function and τ_{mod} are juxtaposed in Figure S11, where uncorrelated noise has been added to both functions. Three curves corresponding to three diameters[1] are shown. Amplitudes of τ_{mod} increase with increasing diameter. The opposite is true for the η-function. For both functions, fluctuation magnitudes (approximate areas swept by the jitter) decrease with increasing diameter, and fluctuations decrease when approaching *ttc*.

Figure S12 suggests that in terms of (median) relative error e, τ_{mod} "wins", and $e\left[\tau_{\mathrm{mod}}(t)\right] \leq e\left[\tau(t)\right]$. This simulation thus supports our claim that the modification of the original τ function leads to an improved noise suppression performance (especially in the initial phase of the approach) – compare also Figure S11*a* with Figure 6*a*. The relative error of the η-function, $e\left[\eta(t)\right]$, tends to increase as *ttc* is approached, and is higher than $e\left[\tau_{\mathrm{mod}}(t)\right]$, particularly for bigger object diameters. For the biggest considered diameter (Figure S12*c*), it even exceeds $e\left[\tau(t)\right]$. The "peculiar" behavior of $e\left[\eta(t)\right]$ is due to the strong decrease of η for $t > t_{\max}$. It reaches small values very quickly, even before *ttc* is actually reached. On the other hand, both τ and τ_{mod} have a minimum short before t_c (section S6.2), and increase afterwards until t_c. Thus, normalization by the noise-free function (denominator of equation S13) causes amplification of $e\left[\eta(t)\right]$ as t_c is approached, while it causes suppression for $e\left[\tau(t)\right]$ and $e\left[\tau_{\mathrm{mod}}(t)\right]$, respectively.

[1] These results generalize readily to the l/v-ratio, assuming a constant speed.

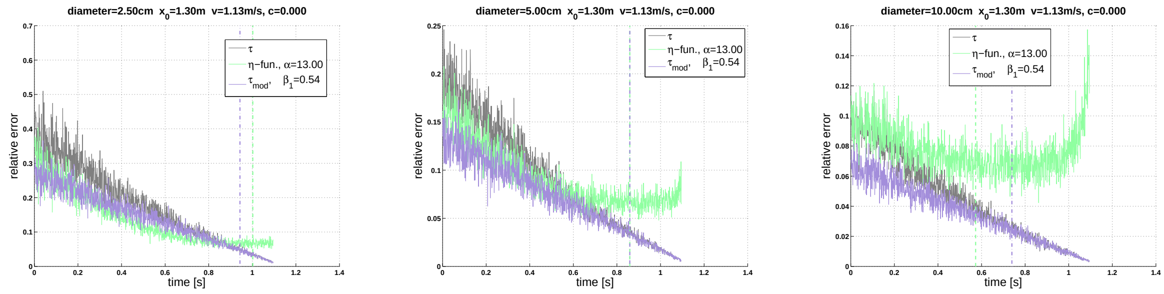

(a) diameter=25 *cm* (b) 50 *cm* (c) 100 *cm*

Figure S12: **Relative error for three object sizes (τ_{mod} and η-function).** This figure shows the median relative error (equation S13, 999 random trials) for τ_{mod} and the η-function, respectively (uncorrelated noise $c = 0$, identical noise was added to all curves). As in the previous figure, $t_{\max} = 0.75 t_c = 0.86\, s$ at object diameter 5 *cm*. For comparison, the ordinary τ function is also shown. The first observation is that the relative error of τ and τ_{mod}, respectively, decreases as *ttc* is approached, at which τ_{mod} has the edge over τ. Furthermore, relative errors of τ_{mod} and τ decrease with diameter. The η-function, however, reveals a more complicated behavior, because it decreases faster proximately before *ttc* than τ or τ_{mod} (both of which increase again upon approaching t_c, see section S6.2). In the initial phase of the approach, the relative error of η decreases with increasing diameter as well. However, close to t_c, the error does not decrease further for the smallest diameter, and even grows again for diameters 50 *cm* and 100 *cm*. The increase in relative error is due to the fact that η approaches zero very quickly after the maximum (faster for bigger diameters). As a consequence, the denominator of equation (S13) gets very small, what blows up the relative error.

Figure S13 illustrates how the integrated relative error E depends on object diameter and noise correlation. Both $E(\tau_{\text{cm}})$ and $E(\eta)$ decrease with increasing diameter. The smallest values for E are measured if identical noise is added to both angular variables Θ and $\dot{\Theta}$ (correlated noise, $c = 1$). The biggest values of E are measured for $c = -1$, and intermediate values of E are obtained for $c = 0$. Thus, the "less" the correlation, the "bigger" the errors.
We also observed a dependence on the time $t_{\max}$ when both functions attain their maximum amplitude (α and β_1 were chosen such that η and τ_{mod}, respectively, had identical $t_{\max}$). Changing $t_{\max}$ of τ_{mod} translates into a moderate change in $E(\tau_{\text{mod}})$, and the linear dependence of $E(\tau_{\text{mod}})$ on object diameter remains unaffected. For the η-function, the corresponding change in $t_{\max}$ is accompanied by a comparatively dramatic change in the nonlinear dependence of $E(\eta)$ on object diameter. Again, this behavior is a result of that the relative error of η is amplified after its maximum, where η quickly approaches zero.

S3.2 Corrected Modified Tau

In the main text it has been shown that the *corrected m-Tau*-model (τ_{cm}, equation 5) is constrained by two "limit functions", such that $\tau(t) < \tau_{\text{cm}}(t) < \tau_{lp}(t)$. These results also apply to the noisified version of the *corrected m-Tau*-model:

$$\tau_{\text{cm}}(t) \equiv \underbrace{\frac{\tilde{\Theta}}{\dot{\tilde{\Theta}} + \beta}}_{\tau_{\text{mod}}(t)} + \underbrace{\frac{\beta \vartheta}{\dot{\vartheta}(\dot{\vartheta} + \beta) + \epsilon}}_{\Delta \tau_{corr}} \tag{S15}$$

Thus, for the noisified angular size $\tilde{\Theta}$ and angular velocity $\dot{\tilde{\Theta}}$, we have $\tau = \tilde{\Theta}/\dot{\tilde{\Theta}}$. Likewise, $\tau_{lp} = \vartheta/\dot{\vartheta}$, but with $\tilde{\Theta}$ and $\dot{\tilde{\Theta}}$ replacing Θ and $\dot{\Theta}$, respectively, in equation (4).
Figure S14 shows the relative error e for three object diameters (footnote 1). It suggests that $e[\tau(t)] \geq e[\tau_{\text{cm}}(t)] \geq e[\tau_{lp}(t)]$. Important, $e[\tau_{\text{cm}}(t)]$ clings to $e[\tau_{lp}(t)]$, albeit all $\beta_i = 1$ (i.e. far away from the limit case $\beta \gg 1$). In other words, no particularly big values of β_i are necessary for achieving a high noise suppression.
Figure S15 shows $E(\tau_{\text{cm}})$ along with the integrated relative error of the limit functions. Less noise correlations increase the E of all three functions, with E showing an approximately linear decrease with object diameter. Increasing the diameter causes an increasing separation $|E(\tau_{\text{cm}}) - E(\tau_{lp})|$. The separation is most prominent for $c = 1$, where $E(\tau_{\text{cm}})$ matches $E(\tau_{lp})$ at the smallest diameter, but $E(\tau_{\text{cm}}) \approx E(\tau)$ at the biggest diameter. For the sake of clarity, the lines in Figure S15 connect the (gray) symbols of each respective function with $c = 0$.

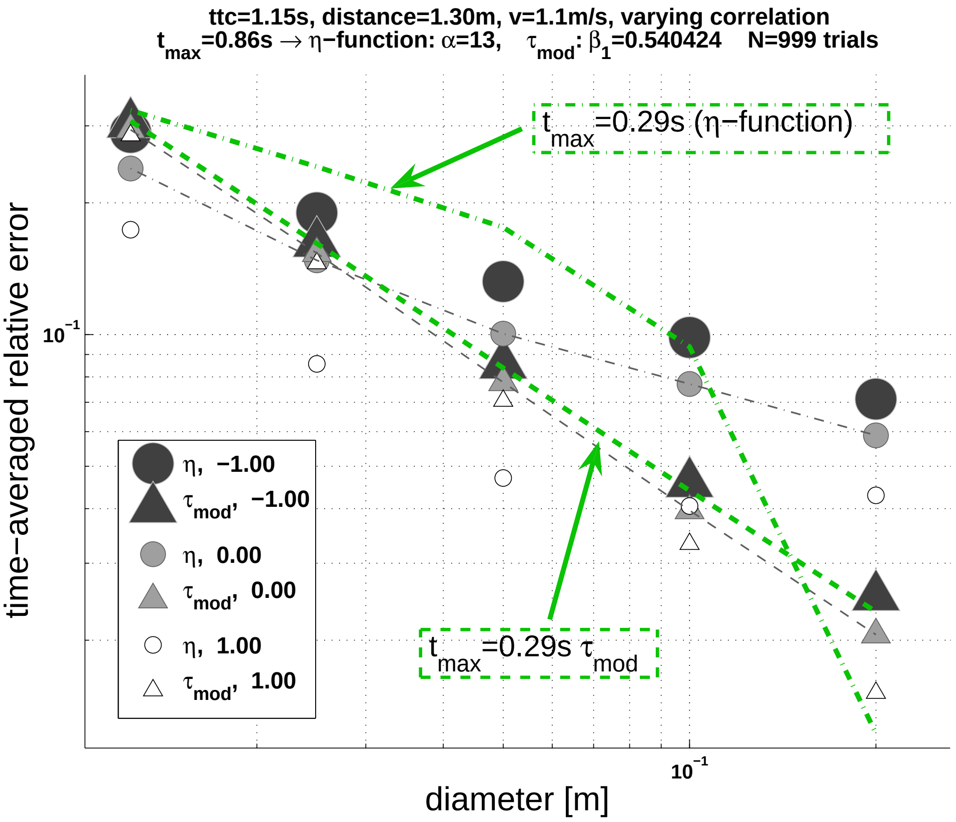

Figure S13: **Noise suppression and noise correlations (τ_{mod} versus η-function).** The integrated error E (equation S14) of η and τ_{mod} is shown for different diameters of approaching objects, and for different noise correlation coefficients c (equation S12). As in the two previous figures, $t_{\max} = 0.75 t_c = 0.86\,s$ at object diameter $5\,cm$. In addition, the bold green curves identified by arrows and corresponding text boxes show $E(\tau_{\text{cm}})$ and $E(\eta)$, respectively, for $t_{\max} = 0.25 t_c = 0.29\,s$ at object diameter $5\,cm$ for uncorrelated noise. The relative error of τ_{mod} increases moderately for $t_{\max} = 0.29\,s$ ($\beta_1 = 0.06$) with respect to $t_{\max} = 0.86\,s$ ($\beta_1 = 0.54$), and curves remain at an approximately linear decrease with increasing object diameter. The relative error of the η-function increases with diameter for $t_{\max} = 0.86$ ($\alpha = 13$), but decreases if its maximum is located earlier at $0.29\,s$ ($\alpha = 39$). Adding identical noise (i.e. $\xi_1 = \xi_2$) to the angular variables ($c = 1$, open symbols) give the smallest errors, because fluctuations in the angular variables are in the same direction and can cancel each other: In the early phase of the approach, $E(\tau_{\text{cm}}) > E(\eta)$, but in the late phase the opposite holds true. Negative noise correlations ($c = -1$, black symbols) lead to the highest values of E for both functions, because fluctuations of optical variables go in opposite directions and add to each other. A somewhat better noise suppression performance (that is, smaller values of $E(\tau_{\text{cm}})$ and $E(\eta)$ at all diameters) is observed for uncorrelated noise ($c = 0$, grey symbols).

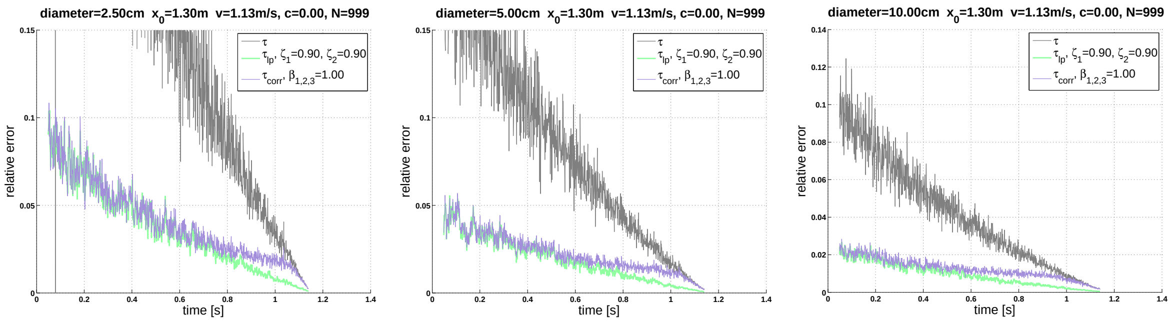

(a) diameter=25 cm (b) 50 cm (c) 100 cm

Figure S14: **Relative error for three object sizes (*corrected m-Tau*).** This figure shows the median relative error (equation S13, 999 random trials) for τ_{cm} and its two limit functions τ and τ_{lp}, respectively (uncorrelated noise). For the comparatively small values of $\beta_i = 1$ chosen here, the noise suppression performance of τ_{cm} is more similar to that of τ_{lp}, rather than τ. This difference, however, gets smaller as ttc is approached, because the values of angular variables increase nearly exponentially while the noise level stays constant. Noise suppression performance also depends on object size: Doubling the diameter divides the relative error by two.

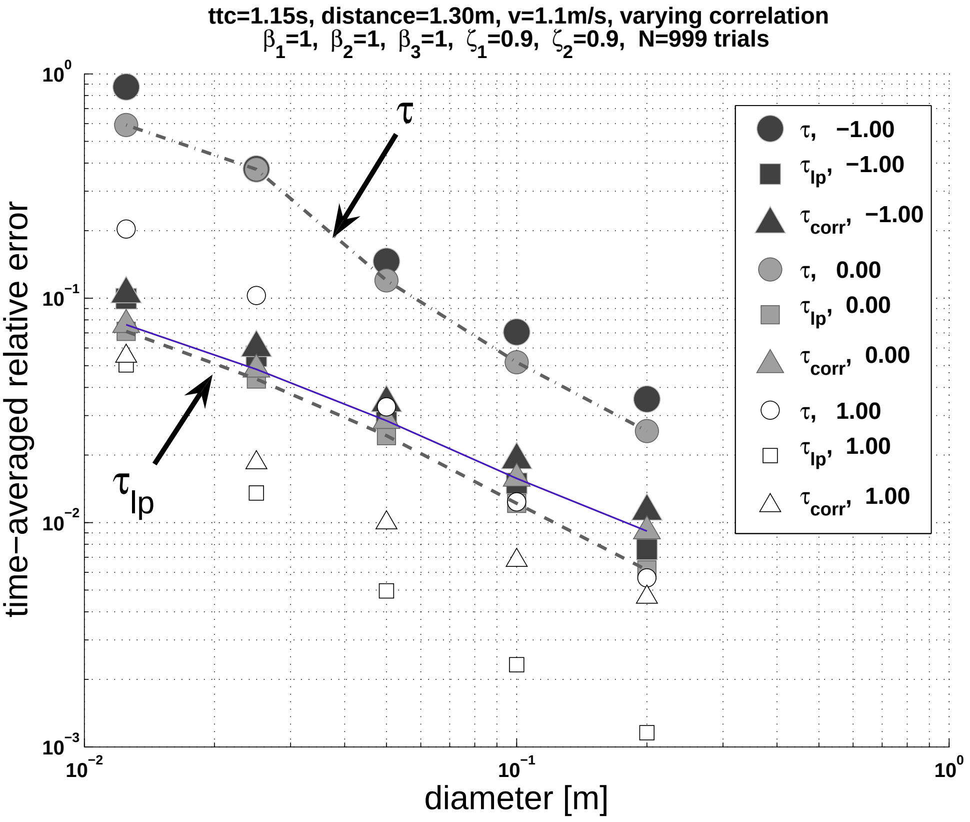

Figure S15: **Noise suppression and noise correlations (*corrected m-Tau*).** The figure shows the integrated error (equation S14) for different diameters of approaching objects, and also for different noise correlation coefficients c (equation S12): $E(\tau_{\rm cm})$ (with all $\beta_i = 1$) is juxtaposed with the integrated error of its two limit functions $E(\tau)$ and $E(\tau_{lp})$. The broken lines that are identified by arrows represent $E(\tau)$ and $E(\tau_{lp})$, respectively, for $c = 0$, while the continuous line denotes $E(\tau_{\rm cm})$ for $c = 0$. If identical noise is added to optical variables ($c = 1$, open symbols), then the smallest errors E are obtained, because fluctuations will cancel each other, as they occur in the same direction. Negative noise correlations ($c = -1$, black symbols) lead to the worst noise suppression performance (= highest values of E). Uncorrelated noise ($c = 0$, grey symbols) causes a somewhat better suppression. Notice that for the quite "moderate" values $\beta_i = 1$ chosen here, $\tau_{\rm cm}$ is only slightly worse than τ_{lp}, which copes best with noise. The latter is true except for $c = 1$: For correlated noise, $E(\tau_{\rm cm})$ is close to $E(\tau_{lp})$ for small object diameters, but approaches $E(\tau)$ with increasing diameter.

References

1. Horn B, Yajun F, Masaki I (2009) Hierarchical framework for direct gradient-based time-to-contact estimation. In: IEEE Intelligent Vehicles Symposium. IEEE, pp. 1394-1400. doi: 10.1109/IVS.2009.5164489.

2. Alenya G, Negre A, Crowley J (2009) A comparison of three methods for measure of time to contact. In: IEEE/RSJ International Conference on Intelligent Robots and Systems, 2009 (IROS 2009). IEEE/RJS, pp. 4565-4570. doi:10.1109/IROS.2009.5354024.
