# Supplementary material for: Unifying Time to Contact Estimation and Collision Avoidance across Species
Source: PLoS Comput Biol. 2012 Aug 16;8(8):e1002625. doi: 10.1371/journal.pcbi.1002625 (PMC3420976; doi:10.1371/journal.pcbi.1002625)
Supplement: Text S4 — Fitting m-Tau and -function to neuronal recordings. Text S4 juxtaposes the individual fitting results of and to a variety of previously published neural recording data, which served to compile Figure 2. Further summary results are presented along with fitting results of individual recording traces. (PDF) [file pcbi.1002625.s004.pdf]

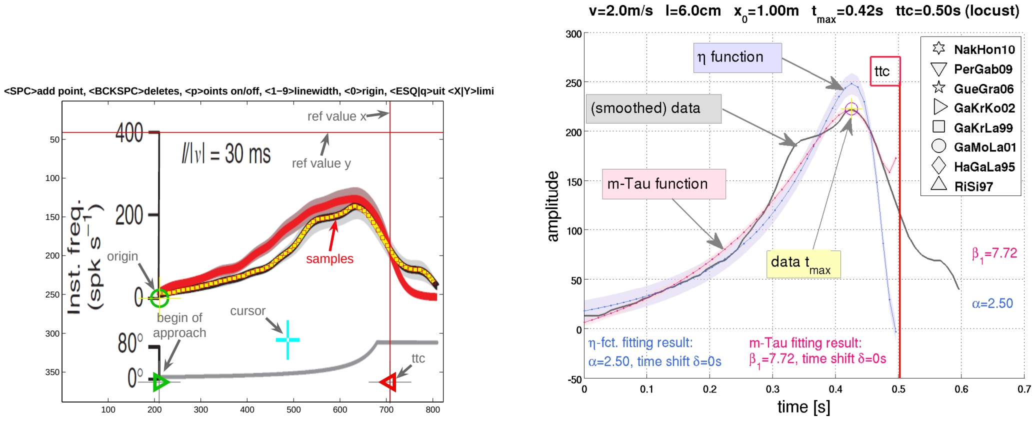

(a) curve sampling app

(b) symbols & curves

Figure S16: **Fitting in a nutshell.** The data shown in this figure come from Figure 4a in reference [1] (complete fitting results are shown in Figure S24*b*). **(a)** We developed an application for manually sampling neuronal response curves from screenshots or scans of published figures. The screenshot shown here illustrates a typical session, where the yellow curve and the small red boxes represent the manually sampled data. Sampling means to place the cursor upon a point on the curve, and to click it in order to record its position. **(b)** Illustration of the fitting procedure for τ_{mod} and η-function, respectively. Data from the same paper are represented by the same graphical symbol in all following figures, and can be further distinguished by their associated halfsize to velocity ratio l/v. Only in a few occasions we have ambiguities. Legend labels denote the references: *NakHon10*= [2], *PerGab09*= [1], *GueGra06*= [3], *GaKrKo02*= [4], *GaKrLa99*= [5], *GaMoLa01*= [6], *HaGaLa95*= [7], and *RiSi97*= [8].

S4 Fitting m-Tau and η-Function to Neuronal Recordings

The prevailing model for describing neuronal recordings of collision sensitive neurons is the η-function [7]. Its distinguishing feature is an activity peak at time t_{max} (Figure S1), which is related to an angular size threshold [5]. The m-Tau function τ_{mod} reveals also an activity peak (Figure 1). In this section, we fit the η-function and τ_{mod} to neuronal recordings from collision sensitive neurons. The neuronal data originate from the figures of eight different publications (Figure S16*a*), totaling 36 data sets, which cover l/v values from $5\,ms$ to $50\,ms$. The fitting model for the η-function was

$$A\dot{\Theta}(t+\delta)e^{-\alpha\Theta(t+\delta)} + o \tag{S16}$$

with A = amplitude, δ = temporal delay, o = offset. The parameter values to be determined by the fitting procedure were either for $\{A, \delta, o, \alpha\}$ or for $\{A, o, \alpha\}$ (with $\delta = 0$). For fitting τ_{mod}, we used the model

$$A\frac{\Theta(t+\delta)}{\dot{\Theta}(t+\delta)+\beta_1} + o \tag{S17}$$

The set of fitting parameters comprises either $\{A, \delta, o, \beta_1\}$ or $\{A, o, \beta_1\}$ (with $\delta = 0$).
For each curve, we tried two fitting algorithms: *(i)* Trust-Region (**TR**, [9, 10]), and *(ii)* Levenberg-Marquardt (**LM**, [11]). As mentioned, each fitting algorithm was executed with either three or four fitting parameters. The best result of the latter four combinations in terms of goodness of fit measures was selected, and is indicated in the legend of each figure. For example, *TR:3* means that three parameter values were determined by the Trust-Region algorithm (e.g. $\{A, o, \beta_1\}$ to fit equation S17 to neuronal

data).

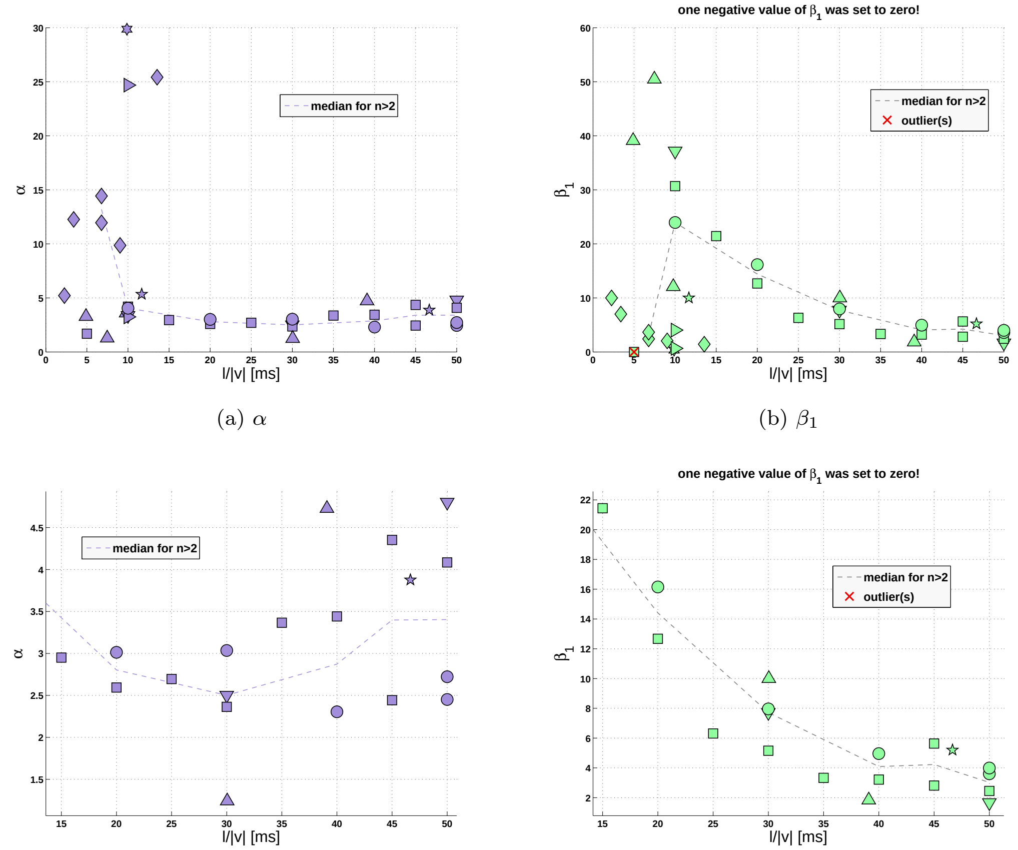

(a) α (b) β_1

(c) α (magnified) (d) β_1 (magnified)

Figure S17: **Fit results I.** (Original neuronal data are identified by their symbols – see Figure S16*b*). **(a)** α versus l/v (η-function fit equation S16). **(b)** β_1 versus l/v (τ_{mod} fit equation S17). **(c)** Same as *a*, but in a magnified representation. **(d)** Same as *b*, but in a magnified representation. The broken lines denote the median value if more than one value of β_1 (or α) was available at some l/v.

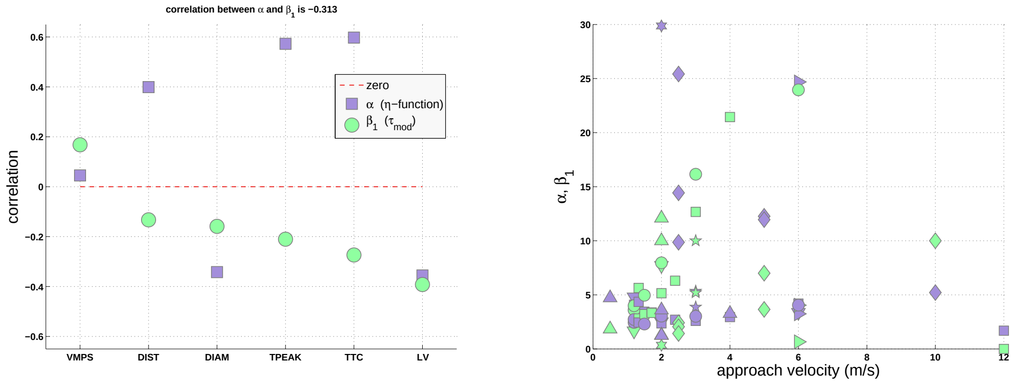

(a) correlation (b) velocity

Figure S18: **Fit results II: correlation & velocity.** **(a)** Correlation of α (square symbols) and β_1 (circle symbols), respectively, with various stimulus parameters (abscissa labels $VMSP = v$, $DIST = x_0$, $DIAM = 2l$, $TPEAK = t_{\max}$, $TTC = t_c$, and $LV = l/v$). The correlation between α and β_1 is -0.313. **(b)** α (blue symbols) and β_1 (green symbols), respectively, as a function of approach velocity v. The mapping from symbols to papers is explained by Figure S16*b*.

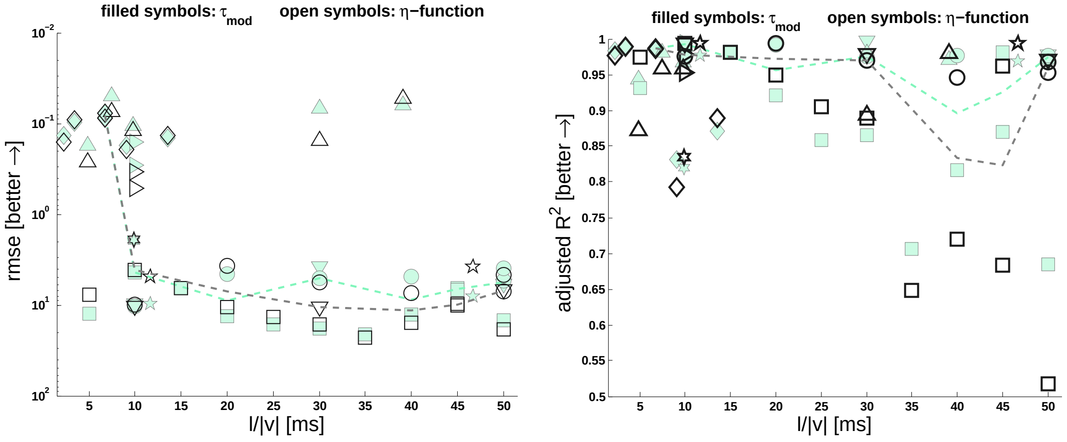

(a) rmse (b) adjusted R^2

Figure S19: **Fit results III: Goodness of fit measures. (a)** Root mean square error ("rmse") between the fitted function (τ_{mod}: filled symbols; η: open symbols) and the original neuronal data. **(b)** Degree-of-freedom adjusted coefficient of determination. If more than one values of rmse and R^2, respectively, were available at some l/v, the corresponding median value was computed. The median is indicated by broken lines (green=τ_{mod}, black=η-function).

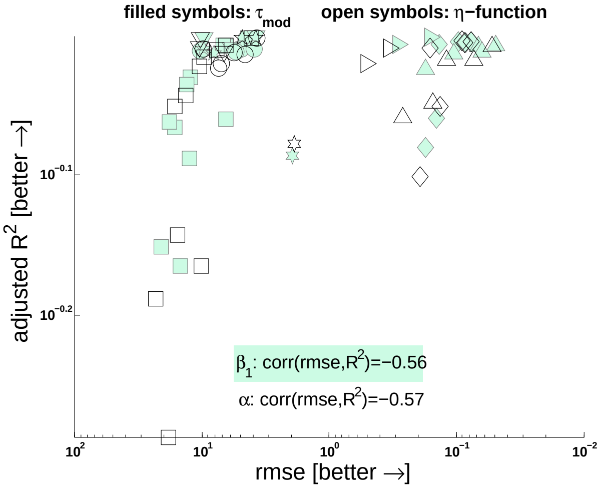

Figure S20: **Fit results IV: rmse versus adjusted R^2.** The figure plots the degree-of-freedom adjusted coefficient of determination against root mean square error (rmse). Filled symbols correspond to fitting of τ_{mod} (correlation $-0,56$ between both measures), and empty symbols to the η-function (with a correlation coefficient of -0.57). Symbols are explained by the legend of Figure S16*b*.

S4.1 Summary Results

In this section, we report summary results for fitting equations (S16) and (S17), respectively, to neuronal recording data from previously published studies. Figure S17 shows how α and β_1 of the fitted η-function and τ_{mod}, respectively, depend on $\kappa \equiv l/v$. One can hardly recognize any trend in the α for the previously published data. Especially if κ is small, $\alpha(\kappa)$ and $\beta_1(\kappa)$ are broadly scattered. This scattering can be explained by Figures S11 to S13, where higher noise levels are observed for smaller diameters (or high approach velocities, respectively). The zoomed-in representation suggests a certain trend across κ for $\beta_1(\kappa)$ (Figure S17*d*), but to a lesser extent for $\alpha(\kappa)$ (Figure S17*c*).
Figure S18 visualizes the correlation of α and β_1 with stimulus parameters. The time of the maximum t_{max}, and also t_c, are most correlated with α. Except for speed and object diameter, the correlation coefficients of β_1 and α have opposite signs, and absolute values are higher for α than for β_1 (except for speed). β_1 anti-correlates "best" with l/v, and with t_c in the second place. The overall correlation between α and β_1 is -0.313.
Figure S19 and S20 show goodness of fit measures. The figures suggest that both functions provide reasonable fits for the neuronal data, as root mean square errors (rmse) and adjusted coefficient of

determination, respectively, are similar for both functions.
The symbols shown in Figure 2 and S46 represent t_{max} of the neuronal data as a function of l/v. The end points of the small vertical lines which originate in each symbol represent t_{max} of the respective fitting function. The longer these lines, the higher the mismatch between experimental and fitted t_{max}. If these absolute differences are integrated, then the fits with the τ_{mod} function predict somewhat better the t_{max} of the neuronal data (0.645 versus 0.948 of the η-function). An approximate linear relationship between t_{max} and l/v is observed for the points connected by lines. Each line connects data points from a single study. Across studies, however, linearity is less obvious. This lack of overall linearity may be attributed to differences in measuring protocols, data evaluation, and sample variation. Notice, however, that t_{max} is not affected by changes in the body temperature of locusts, nor does it seem to be affected by changes in background light intensity levels or stimulus contrast [5].

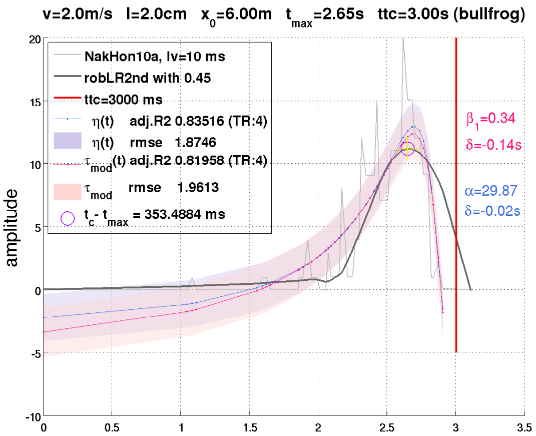

Figure S21: **NakHon10.** Bullfrog *rana catesbeiana*, $l/v = 10\,ms$, figure 8 and 9, respectively, from reference [2]. The stimulus was a $35\,mm \times 35\,mm$ black square ($l \approx 2\,cm$), $t_{\max}$ at around $2600\,ms$ ($400\,ms$ before *ttc*). (Abscissa in units of seconds).

S4.2 Detailed Results

Figures S21 to S35 show details on fitting results. Each figure legend has a label that describes from which paper the neuronal data were taken. In addition, the figure legends juxtapose goodness-of-fit measures for η and τ_{mod}, along with the fitted values of α, β_1, and δ.
The target data for fitting are always plotted with a thick and dark gray line. Apparently noisy neuronal data were smoothed before fitting took place. In that case, the original neuronal data are drawn with a thin and light gray line, and the smoothing algorithm (along with corresponding parameter value) is shown in the figure legend:

"*robLR1st*" robust local regression using weighted linear least squares and a *1st degree* polynomial model. The parameter value specifies the span (= number of data points for computing a smoothed value) in terms of percentage of the total number of data points.

"*robLR2nd*" robust local regression using weighted linear least squares and a *2nd degree* polynomial model. The parameter value specifies the span in terms of percentage of the total number of data points.

"*sgolay*" Savitzky-Golay method with polynomial degree 2. The parameter value p denotes the span in terms of number of data points is $2p + 1$.

If no clear response peak could be detected in the neuronal recording data, or if the data points were separated by too big time intervals, we proceeded with selecting a smoothing algorithm. In that case, the tuning criteria were *(i)* to leave the original data as less distorted as possible, and *(ii)* to assure, by visual inspection, a sound location of the response peak in the smoothed data. This is to say that $t_{\max}$ of the smooth data should coincide with where an experienced observer would place it.
If smoothing was applied to the data from reference [8], a response peak would be exposed. In all considered data sets, the peak was predicted to occur *before ttc*. This is an interesting observation, given the claim of Rind & Simmons that a response peak before t_c is an artifact "*due to failure to stimulate the eye with sufficiently small and frequent jumps in image edges*". The *original* response curves from reference [8] are overly rugged, and do not allow a precise statement as to the localization of $t_{\max}$.

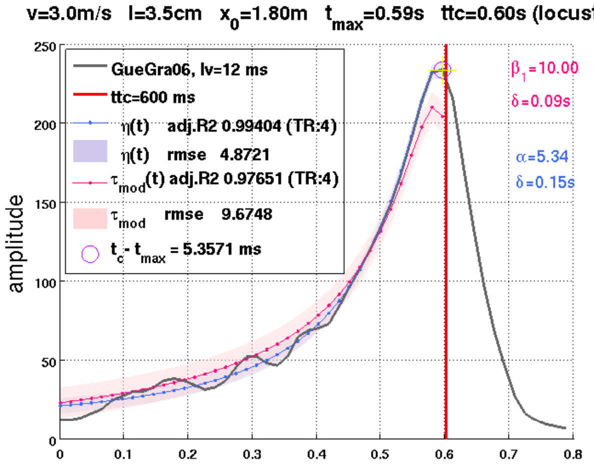

(a) $l/v = 11.7\,ms$

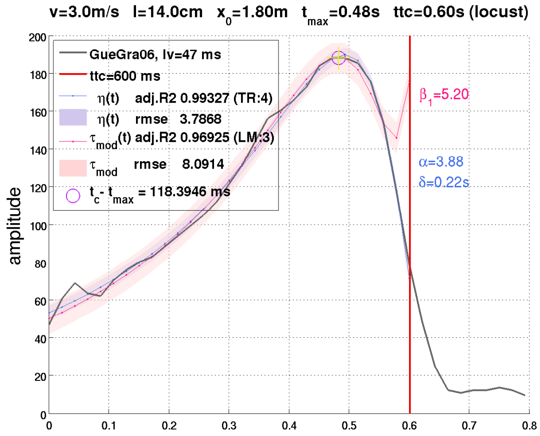

(b) $l/v = 46.7\,ms$

Figure S22: **GueGra06.** Locust. Figure from reference [3]. The stimulus was a black disk. (Abscissa in units of seconds).

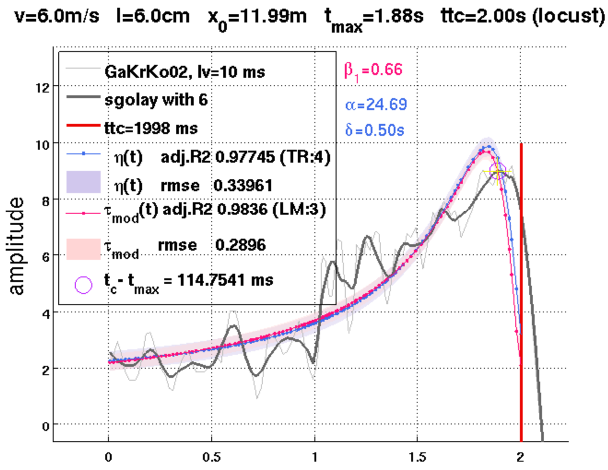

(a) control

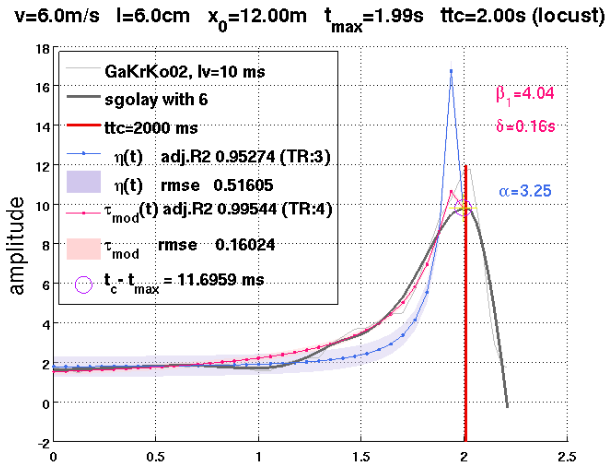

(b) TTX-injection

Figure S23: **GaKrKo02.** Locust. Figure 4b from reference [4], $l/v = 10\,ms$, black squares. (Abscissa in units of seconds).

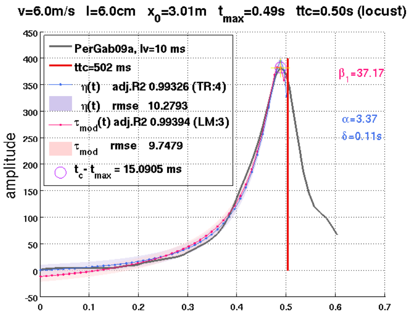

(a) $l/v = 10\,ms$

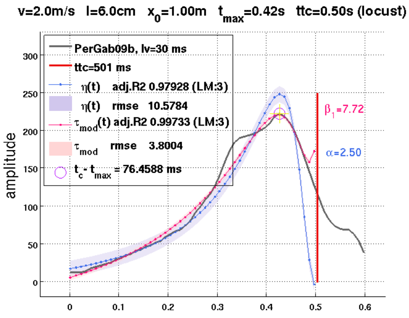

(b) $l/v = 30\,ms$

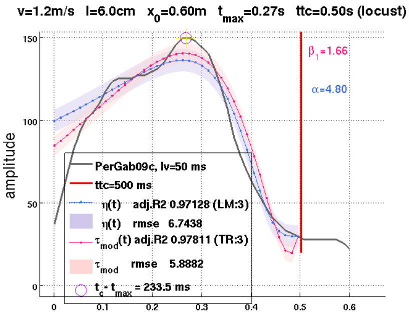

(c) $l/v = 50\,ms$

Figure S24: **PerGab09.** Locust. Figure 4 from reference [1], black disks. (Abscissa in units of seconds).

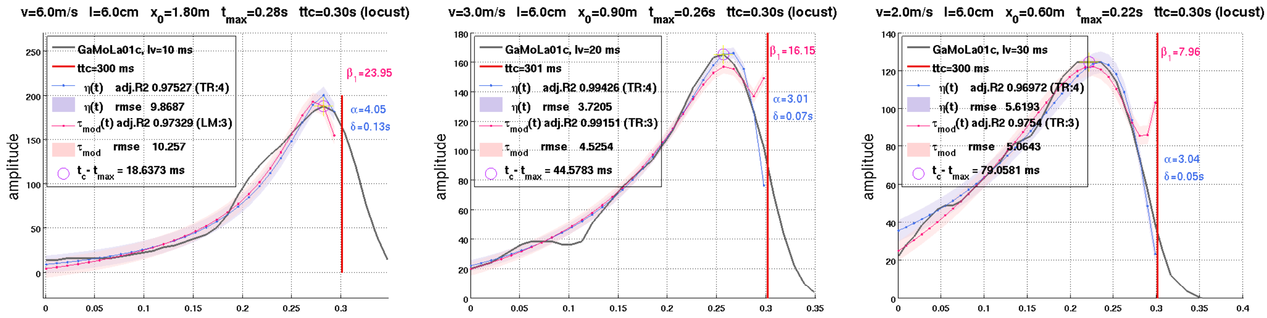

(a) $l/v = 10\ ms$ (b) $l/v = 20\ ms$ (c) $l/v = 30\ ms$

Figure S25: **GaMoLa01 I.** Locust. Figure 1c from reference [6], looming squares. (Abscissa in units of seconds).

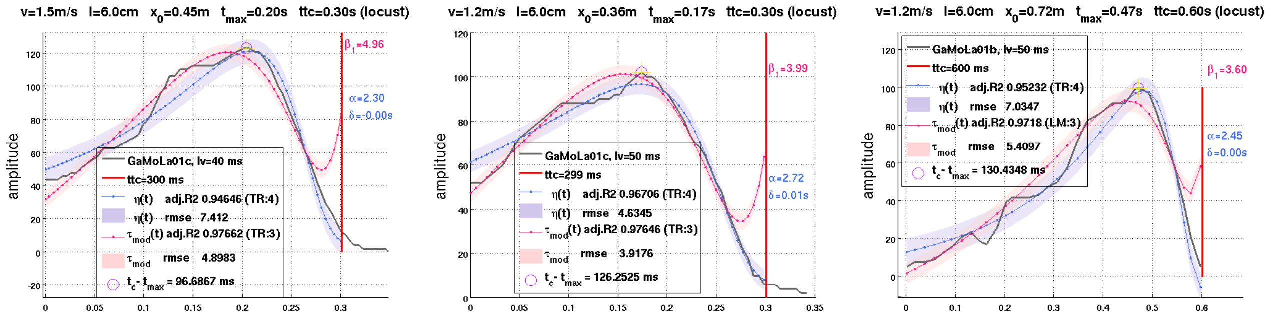

(a) $l/v = 40\ ms$ (b) $l/v = 50\ ms$ (c) $l/v = 50\ ms$

Figure S26: **GaMoLa01 II.** Locust. Figure 1c (a, b) and Figure 1b (c) from reference [6], looming squares. (Abscissa in units of seconds).

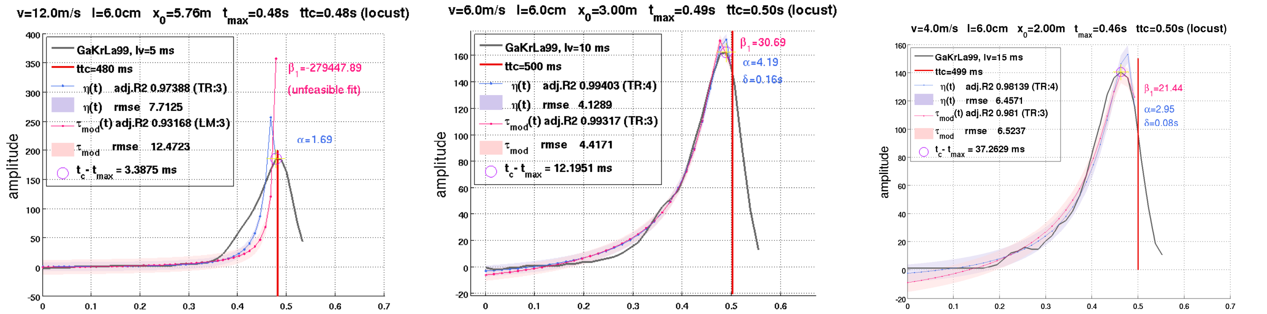

(a) $l/v = 05\ ms$ (b) $l/v = 10\ ms$ (c) $l/v = 15\ ms$

Figure S27: **GaKrLa99 I.** Locust. Figure 3 from reference [5], dark squares. (Abscissa in units of seconds).

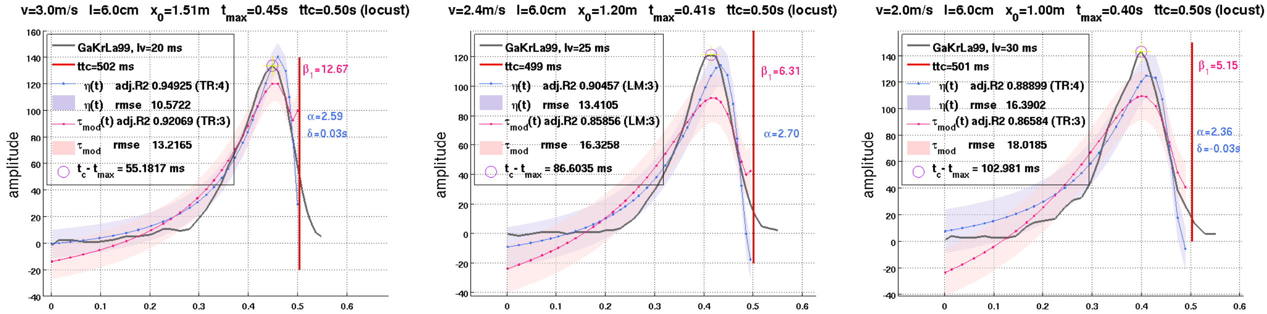

(a) $l/v = 20\ ms$ (b) $l/v = 25\ ms$ (c) $l/v = 30\ ms$

Figure S28: **GaKrLa99 II.** Locust. Figure 3 from reference [5], dark squares. (Abscissa in units of seconds).

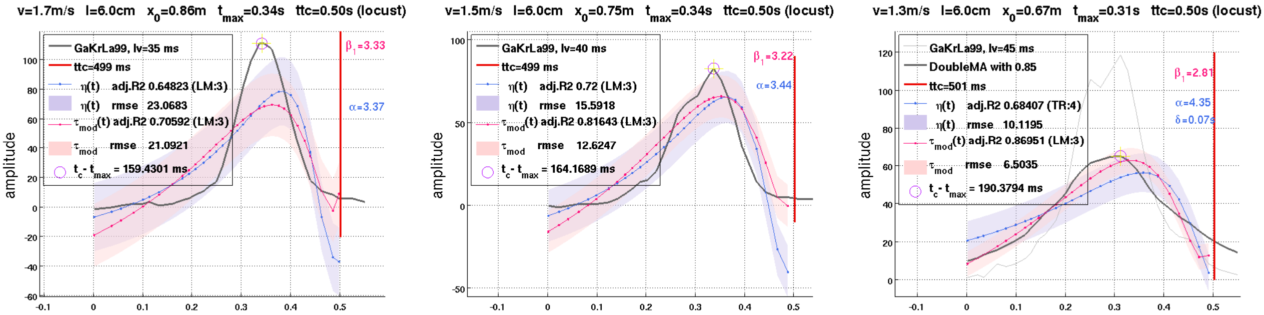

(a) $l/v = 35\ ms$ (b) $l/v = 40\ ms$ (c) $l/v = 45\ ms$

Figure S29: **GaKrLa99 III.** Locust. Figure 3 from reference [5], dark squares. (Abscissa in units of seconds).

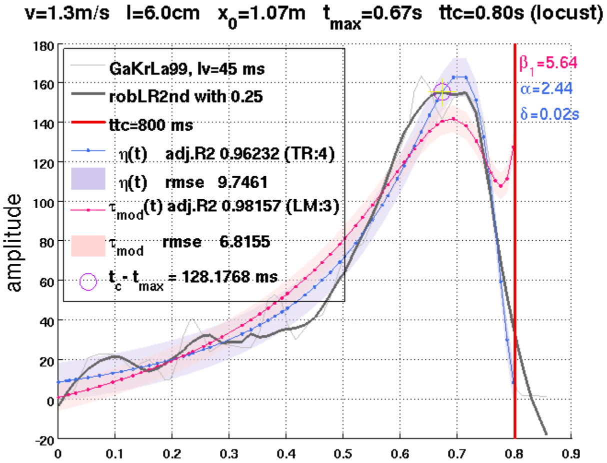

(a) $l/v = 45\ ms$

Figure S30: **GaKrLa99 IV.** Locust. Figure 2 from reference [5], dark squares. (Abscissa in units of seconds).

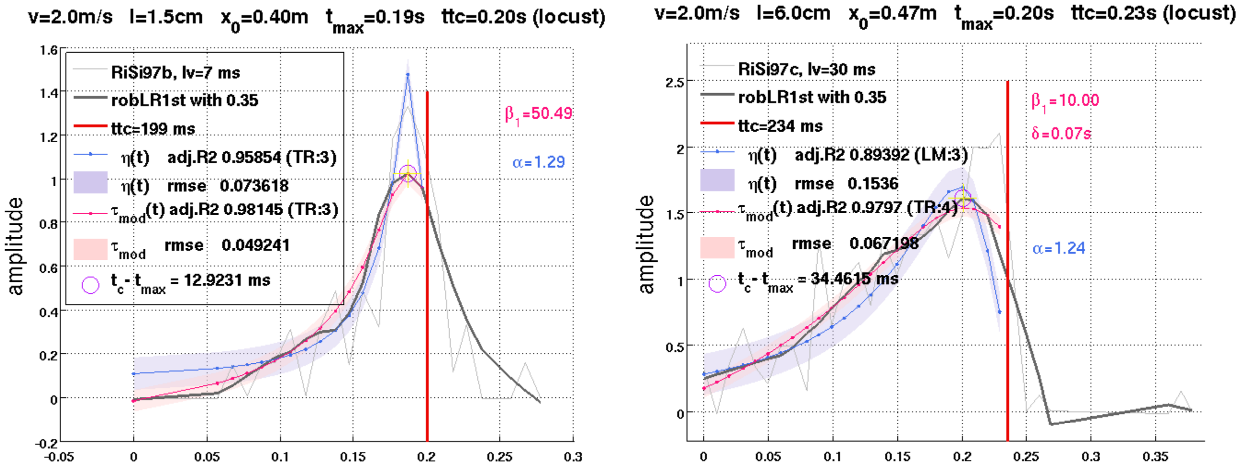

(a) $l/v = 7\ ms$ (b) $l/v = 30\ ms$

Figure S31: **RiSi97 I.** Locust, Figure 1B, C from reference [8], approach speed $v = 2\,m/s$, **(a)** disk 17° ($l \approx 0.015m$) **(b)** disk 62° ($l \approx 0.06m$). (Abscissa in units of seconds).

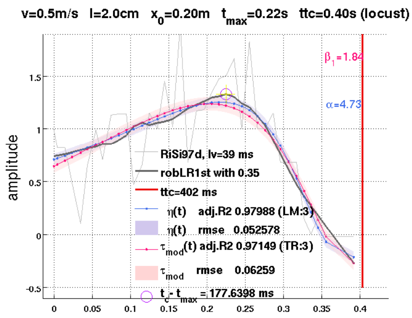

(a) $l/v = 39\,ms$

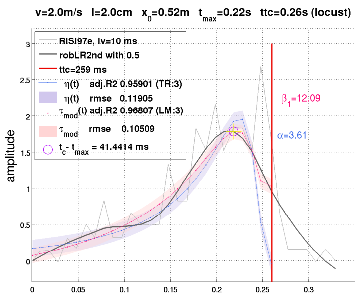

(b) $l/v = 10\,ms$

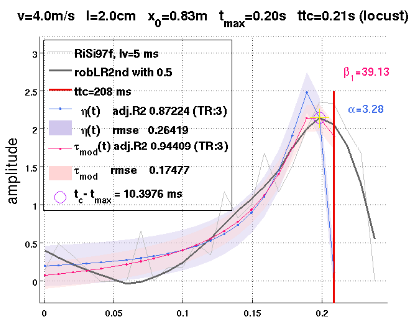

(c) $l/v = 5\,ms$

Figure S32: **RiSi97 II.** Locust, Figure 1D, E from reference [8], 30 mm × 40 mm black rectangle ($l \approx 0.02m$) approach speed **(a)** $v = 0.5\,m/s$, **(b)** $v = 2\,m/s$. **(c)** Figure 1F from reference [8], 30 mm × 40 mm black rectangle ($l \approx 0.02m$, approach speed $v = 4\,m/s$). (Abscissa in units of seconds).

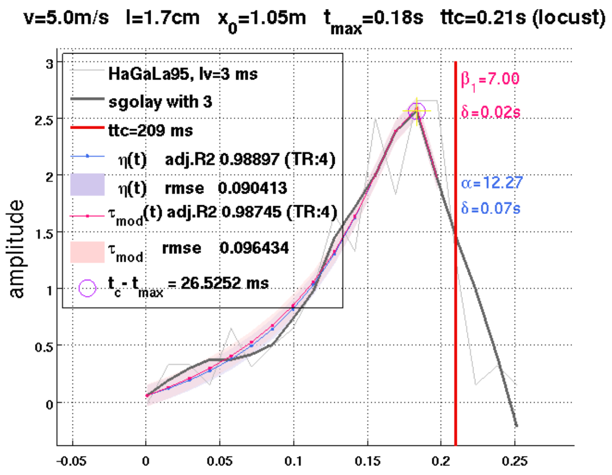

(a) $l/v = 3.39\,ms$

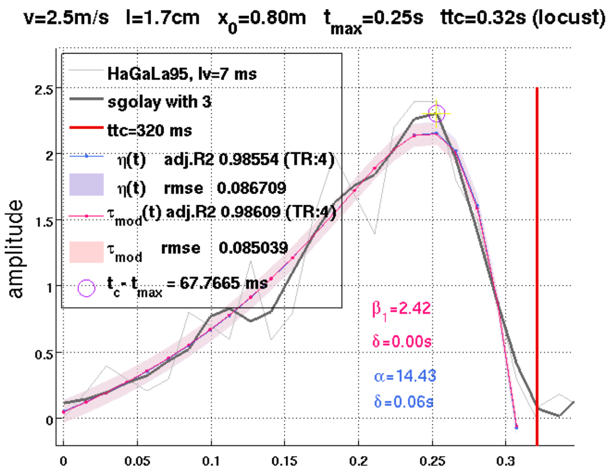

(b) $l/v = 6.77\,ms$

Figure S33: **HaGaLa95 I.** Locust, Figure 3Ai, Aii from reference [7], 3 cm black square ($l \approx 0.017m$) approach speed **(a)** $v = 5\,m/s$, **(b)** $v = 2.5\,m/s$. (Abscissa in units of seconds).

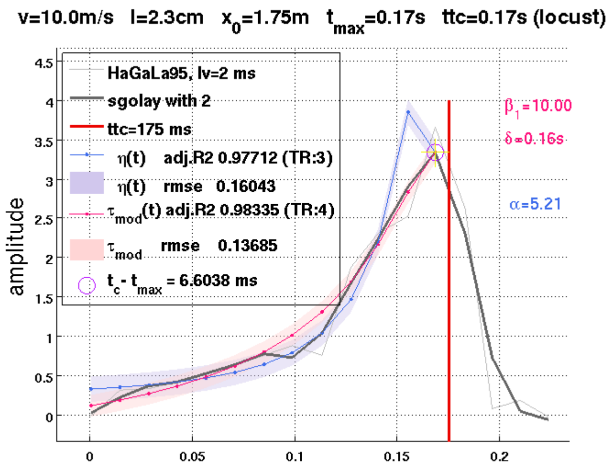

(a) $l/v = 2.26\,ms$

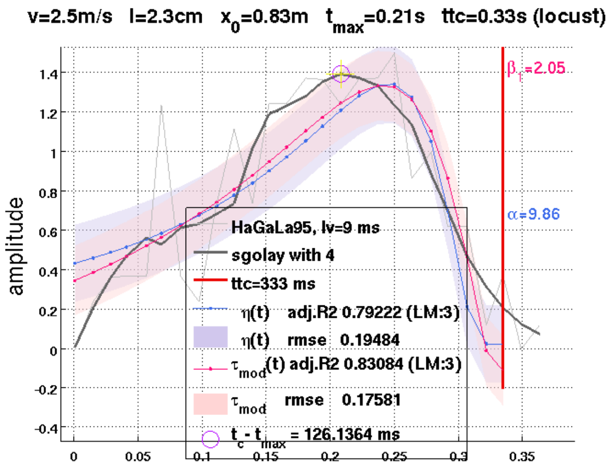

(b) $l/v = 9\,ms$

Figure S34: **HaGaLa95 II.** Locust, Figure 3Bi, Bii from reference [7], 4 cm black square ($l \approx 0.023m$) approach speed **(a)** $v = 10\,m/s$, **(b)** $v = 2.5\,m/s$. (Abscissa in units of seconds).

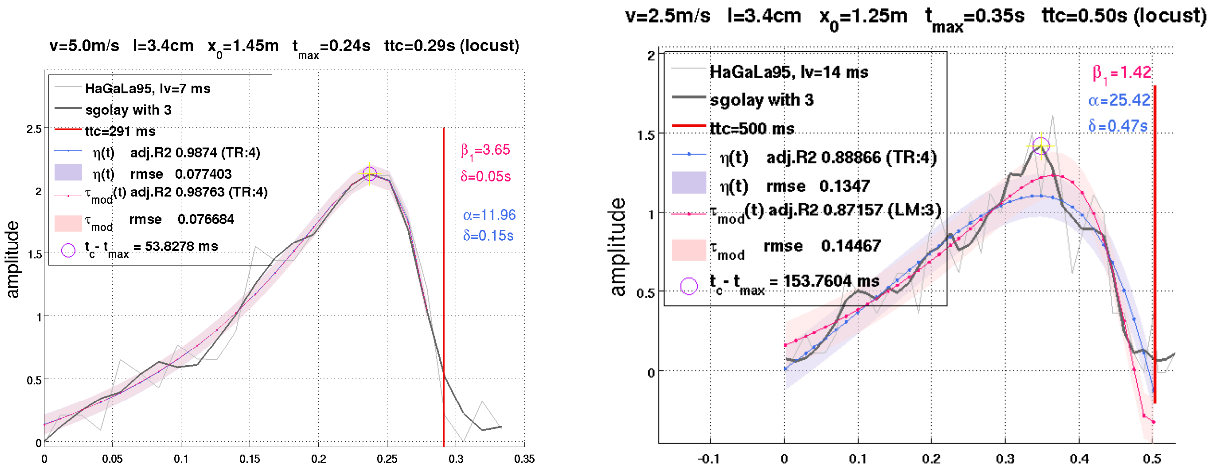

(a) $l/v = 6.77\ ms$

(b) $l/v = 13.54\ ms$

Figure S35: **HaGaLa95 III.** Locust, Figure 3Ci, Cii from reference [7], 6 cm black square ($l \approx 0.034m$) approach speed **(a)** $v = 5\ m/s$, **(b)** $v = 2.5\ m/s$. (Abscissa in units of seconds).

References

1. Peron S, Gabbiani F (2009) Spike frequency adaptation mediates looming stimulus selectivity. Nat Neurosci 12: 318-326.

2. Nakagawa H, Hongjian K (2010) Collision-sensitive neurons in the optic tectum of the bullfrog, rana catesbeiana. J Neurophysiol 104: 2487-2499.

3. Guest B, Gray J (2006) Responses of a looming-sensitive neuron to compound and paired object approaches. J Neurophysiol 95: 1428-1441.

4. Gabbiani F, Krapp H, Koch C, Laurent G (2002) Multiplicative computation in a visual neuron sensitive to looming. Nature 420: 320-324.

5. Gabbiani F, Krapp H, Laurent G (1999) Computation of object approach by a wide-field, motion-sensitive neuron. J Neurosci 19: 1122-1141.

6. Gabbiani F, Mo C, Laurent G (2001) Invariance of angular threshold computation in a wide-field looming-sensitive neuron. J Neurosci 21: 314-329.

7. Hatsopoulos N, Gabbiani F, Laurent G (1995) Elementary computation of object approach by a wide-field visual neuron. Science 270: 1000-1003.

8. Rind F, Simmons P (1997) Signaling of object approach by the DCMD neuron of the locust. J Neurophysiol 77: 1029-1033.

9. Moré J, Sorensen DC (1983) Computing a trust region step. SIAM J Sci Comput 4: 553-572.

10. Byrd R, Schnabel R, Shultz G (1987) A trust region algorithm for nonlinearly constrained optimization. SIAM J Numer Anal 24: 1152-1170.

11. Press H, Teukolsky S, Vetterling W, Flannery B (2007) Numerical Recipes: The Art of Scientific Computing, Third Edition. Cambridge University Press.
