# Supplementary material for: Unifying Time to Contact Estimation and Collision Avoidance across Species
Source: PLoS Comput Biol. 2012 Aug 16;8(8):e1002625. doi: 10.1371/journal.pcbi.1002625 (PMC3420976; doi:10.1371/journal.pcbi.1002625)
Supplement: Text S5 — Finding parameter values for corrected m-Tau . Text S5 describes the optimization procedure for the corrected m-Tau -model “”, with which we obtained the parameter values for the simulation of our psychophysical experiment (e.g. Figure 8). The -parameters were optimized in three different ways: For achieving a good prediction performance of the psychophysical data corresponding to (i) the small object diameter (Table S1 in Text S5), (ii) the big object diameter (Table S2 in Text S5), and (iii) both diameters at the same time (combined; Table S3 in Text S5). The best ten values are listed in their respective tables according to their psychophysical prediction performance (as quantified by score measures and , respectively): The best parameter set (smallest score measure) was assigned rank one, the second best rank two, etc. Several figures were compiled that show an additional analysis of the parameter ranking. (PDF) [file pcbi.1002625.s005.pdf]

S5 Finding Parameter Values for *corrected m-Tau*

S5.1 Error Measures

An "reasonable optimal" set of parameters $\mathcal{P} = \{p_1, p_2, \zeta_1, \zeta_2, \beta_1, \beta_2, \beta_3\, \beta_4\}$ was determined by systematically parsing the parameter space in several search blocks. In each successive block, the search volume was diminished, and the step size for parameters was decreased in turn. This procedure took several weeks on a standard dual core desktop computer, where τ_{cm}-predictions for each object size (*big* & *small*) were processed in parallel. The quality of a candidate $\mathcal{P}$ ("prediction performance") was measured with two functions ("score measures"): root mean square error (*rmse*, E_{rms}), and an outlier insensitive, robust error (*robe*, E_{rob}):

$$E_{rms} = \sqrt{\frac{1}{35}\sum_{i=1}^{5}\sum_{j=1}^{7}\left(\hat{\varrho}_{ij} - \varrho_{ij}\right)^2}$$

$$E_{rob} = \operatorname*{median}_{i\in\{1\ldots5\}}\left(\operatorname*{median}_{j\in\{1\ldots7\}}|\hat{\varrho}_{ij} - \varrho_{ij}|\right) \tag{S18}$$

$\hat{\varrho}_{ij} \equiv \hat{\varrho}(t_{\mathrm{pres}}[i], \hat{t}_c[j])$ is the psychophysical measured proportion of later response (equation 13), and ϱ_{ij} the corresponding prediction of τ_{cm}. For the presentation of our simulation results we plot ϱ as a function of t_c for a fixed value of t_{pres}. In order to quantify how the score measures vary between the t_{pres}, we also calculated the standard deviation (*std*, σ_{rms}), and its robust estimate (via the median absolute deviation; *mad*, σ_{rob}):

$$\sigma_{rms} = \sqrt{\frac{1}{4}\sum_{i=1}^{5}\left(\varrho_i - \operatorname*{mean}_{j\in\{1\ldots5\}}(\varrho_j)\right)^2}$$

$$\sigma_{rob} = 1.4826 \cdot \operatorname*{median}_{i\in\{1\ldots5\}}|\varrho_i - \operatorname*{median}_{j\in\{1\ldots5\}}(\varrho_j)| \tag{S19}$$

Parameter search was carried out independently for object size 5 *cm* (*small*: figure S38 & Table S1) and 10 *cm* (*big*: figure S39 & Table S2). After the search, the parameter sets $\mathcal{P}$ were ordered according to increasing score measure in score tables: The first table row corresponds to the $\mathcal{P}$ with the best prediction performance (smallest score) and was assigned rank one. The second best $\mathcal{P}$ has rank two, and so on. In addition to these two score tables, a third one was computed, with $\mathcal{P}$ that are optimal for both diameters at the same time (*combined* diameters). The computation was done by just averaging the scores of *small* and *big* diameters (for corresponding values of model parameters), and then sort the result according to ascending averaged score measures (figure S40 & Table S3).

S5.2 Simplified Models

We carried out further parameter optimizations as described in the last section with the τ_{lp} model (equation 8; $\mathcal{P} = \{p_1, p_2, \zeta_1, \zeta_2\}$), and the τ_{cm} model with equal values of $\beta_i \equiv \beta\, \forall i = 1, 2, 3$ (thus $\mathcal{P} = \{p_1, p_2, \zeta_1, \zeta_2, \beta\}$)

The smallest found E_{rms} scores for τ_{lp} were: 0.10256 (*small* diameter), 0.12772 (*big*), and 0.11994 (*combined*). These values would correspond to rank 42, 47, and 39, respectively, in the corresponding tables of the fully parametrized τ_{cm} model. The corresponding E_{rob} scores were 0.05, 0.04, and 0.06, corresponding to rank > 100, 30, and 56, respectively.

The smallest E_{rms} score for τ_{cm} with $\beta_1 = \beta_2 = \beta_3$ (and $\beta_4 = 0$) were: 0.096454 (*small*), 0.10737 (*big*), and 0.11461 (*combined*), corresponding to ranks 19, 98, and 80, respectively. The corresponding E_{rob} values were 0.038, 0.028, and 0.05, corresponding to ranks 5, 2, and 39, respectively.

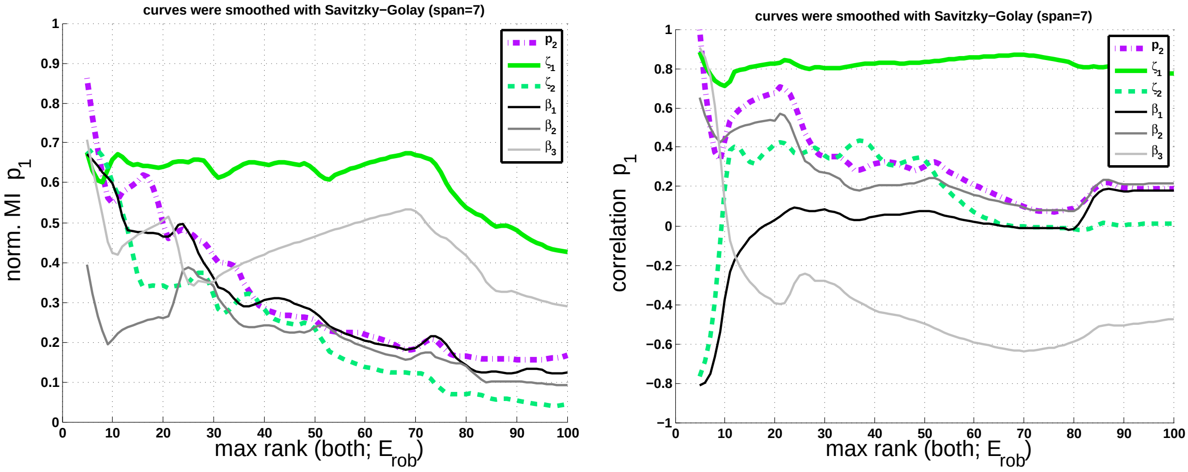

(a) MI p_1 (b) corr. p_1

Figure S36: **Mutual information and correlation of p_1 versus all other parameters.** (Ranking according to E_{rob}, combined sizes). All curves were smoothed with the Savitzky-Golay method (span 7). **(a)** Mutual information. **(b)** Correlation.

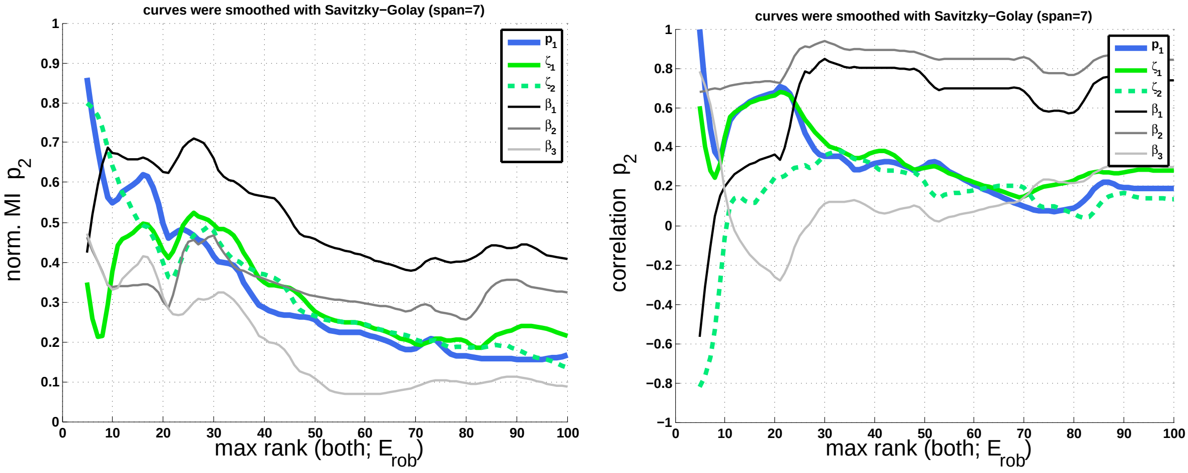

(a) MI p_2 (b) corr. p_2

Figure S37: **Mutual information and correlation of p_2 versus all other parameters.** (Ranking according to E_{rob}, combined sizes). All curves were smoothed with the Savitzky-Golay method (span 7). **(a)** Mutual information. **(b)** Correlation.

S5.3 Relation Between Parameters

One might ask whether some parameters correlate with each other, for example, the noise level p_i with the degree of lowpass filtering ζ_i $(i = 1, 2)$. We accordingly computed the correlation matrix and mutual information across $\mathcal{P}$. Corresponding results strongly depend on the number of included ranks (that is, the number of included parameter values). In almost all cases (i.e. combinations of size and score measures), we were not able to recognize any clear trend.

As an illustration, two exceptions are shown. Figures S36 reveal some mutual dependence of p_1 on ζ_1, both in terms of mutual information and correlation. Furthermore, figure S37 suggests, by means of correlation, some interdependence between p_2 and β_1 on the one hand, and p_2 and β_2 on the other. However, no such trend is revealed in the mutual information plot.

S5.4 Parameter Values for *corrected m-Tau*

Concrete parameter values are given in the Tables S1 (*small*), S2 (*big*), and S3 (*combined* size). Each of the tables shows the parameter values that correspond to the best ten scores achieved, according to E_{rms} and E_{rob}. Because we parsed the parameter space, we are able to visualize how these scores depend on

rank	E_{rms}, *small*	σ_{rms}	p_1	p_2	ζ_1	ζ_2	β_1	β_2	β_3	β_4
1	**0.082615**	0.037771	0.2500	0.1000	0.95	0.90	1.000	1.000	1.500	0.000
2	**0.082780**	0.039662	0.2500	0.1000	0.95	0.90	1.000	1.000	1.500	0.001
3	**0.085673**	0.032655	0.2500	0.1000	0.90	0.90	1.000	1.000	1.500	0.000
4	**0.086420**	0.033155	0.2500	0.1000	0.90	0.90	1.000	1.000	1.500	0.001
5	**0.088668**	0.048796	0.2500	0.1000	0.95	0.90	1.000	1.000	1.500	0.010
6	**0.089901**	0.042124	0.2500	0.1000	0.90	0.90	1.000	1.000	1.500	0.010
7	**0.090810**	0.029306	0.2500	0.1000	0.95	0.50	0.500	0.500	1.000	0.010
8	**0.090833**	0.044017	0.2500	0.1000	0.90	0.95	1.000	1.000	1.500	0.001
9	**0.091337**	0.049700	0.2500	0.1000	0.95	0.95	1.000	1.000	1.500	0.000
10	**0.091387**	0.043601	0.2500	0.1000	0.90	0.95	1.000	1.000	1.500	0.000

rank	E_{rob}, *small*	σ_{rob}	p_1	p_2	ζ_1	ζ_2	β_1	β_2	β_3	β_4
1	**0.036000**	0.005930	0.2500	0.1000	0.90	0.90	1.000	1.000	1.500	0.010
2	**0.036000**	0.023722	0.2500	0.0500	0.90	0.99	1.000	0.500	1.000	0.000
3	**0.036000**	0.023722	0.2500	0.0500	0.90	0.99	1.000	0.500	1.000	0.001
4	**0.036000**	0.026687	0.2500	0.0500	0.90	0.99	1.000	0.500	1.000	0.010
5	**0.038000**	0.005930	0.2500	0.0100	0.90	0.50	0.500	1.000	1.500	0.000
6	**0.038000**	0.005930	0.2500	0.0100	0.90	0.25	0.500	1.000	1.500	0.001
7	**0.038000**	0.002965	0.2500	0.0100	0.90	0.50	0.500	1.000	1.500	0.001
8	**0.038000**	0.005930	0.2500	0.0075	0.90	0.50	0.500	1.000	1.500	0.001
9	**0.038000**	0.029652	0.2500	0.1000	0.95	0.50	0.500	0.500	1.000	0.010
10	**0.038000**	0.005930	0.2500	0.0750	0.90	0.99	5.000	1.000	1.500	0.010

Table S1: The ten best performing parameter sets for the *small* diameter (5 *cm*). The upper table shows parameter sets that are ordered according to increasing E_{rms} score (Figure S38*a*), and the lower table according to E_{rob} scores (Figure S38*b*). Corresponding $\tau_{\rm cm}$-predictions for ϱ with the first five parameter sets are shown in Figures S48 & S49 (E_{rms} score), and Figures S52 & S53 (E_{rob} score).

rank numbers. Corresponding plots are shown in figure S38, S39, and S40.

S5.5 Trial & Error Parameter Adjustment

By means of achieving the subjective best overall match between the psychometric curves $\hat{\varrho}_i$ and corresponding model predictions ϱ_i at each presentation time i ("Chi by eye", [1]), we arrived at the the parameter set $\mathcal{P} = \{p_1, p_2, \zeta_1, \zeta_2, \beta_1, \beta_2, \beta_3\, \beta_4\} = \{0.035, 0.035, 0.9, 0.9, 2.5, 1, 0.8, 0\}$.

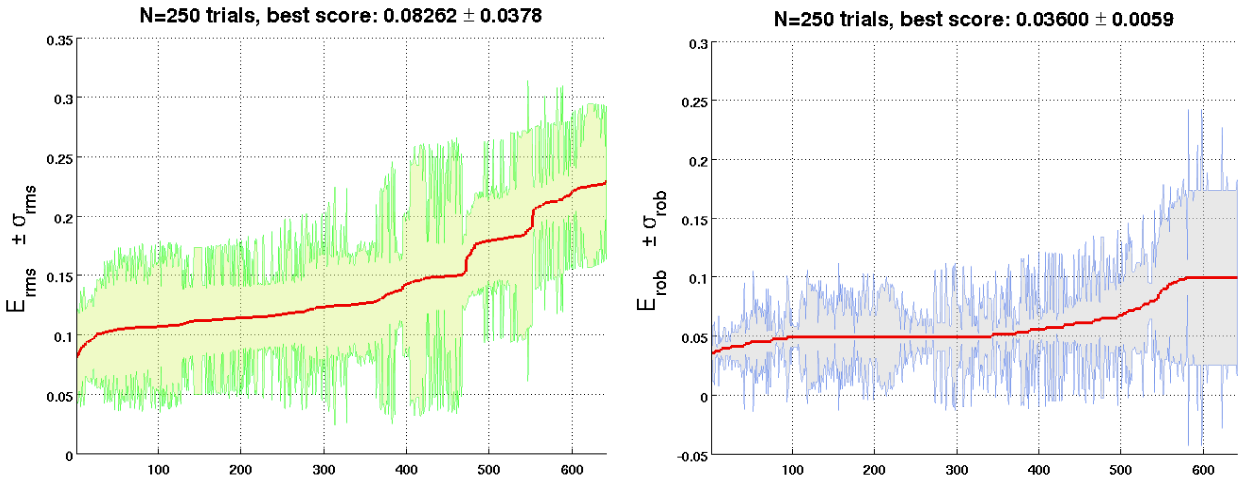

(a) $E_{rms} \pm \sigma_{rms}$ (size 5 *cm*) (b) $E_{rob} \pm \sigma_{rob}$ (size 5 *cm*)

Figure S38: τ_{cm}-scores for diameter 5 *cm* (*small*). Abscissa values show the rank of parameter sets in Table S1. The red lines indicate E_{rms} score and E_{rob} score. The colored areas denote variation σ_{rms} and σ_{rob}, respectively, and capture the variability across presentation times. A characteristic feature of these plots is the step-wise increment of scores (see also figures S39 & S40). They indicate abrupt changes in the curve shape of τ_{cm}-predictions for ϱ.

rank	E_{rms}, *big*	σ_{rms}	p_1	p_2	ζ_1	ζ_2	β_1	β_2	β_3	β_4
1	**0.098490**	0.026236	0.0750	0.0750	0.25	0.90	10.000	2.000	1.500	0.000
2	**0.098708**	0.033911	0.0750	0.1000	0.90	0.90	10.000	2.000	1.500	0.000
3	**0.098949**	0.025984	0.0750	0.0750	0.25	0.90	10.000	2.000	1.500	0.001
4	**0.099057**	0.033675	0.0750	0.1000	0.90	0.90	10.000	2.000	1.500	0.001
5	**0.100974**	0.100943	0.1000	0.0250	0.99	0.95	0.100	1.000	1.500	0.010
6	**0.101449**	0.025393	0.0750	0.0750	0.50	0.90	10.000	2.000	1.500	0.000
7	**0.101648**	0.025423	0.0750	0.0750	0.50	0.90	10.000	2.000	1.500	0.001
8	**0.101872**	0.097396	0.1000	0.0100	0.99	0.99	0.050	0.500	1.000	0.001
9	**0.101994**	0.096591	0.1000	0.0100	0.99	0.99	0.050	0.500	1.000	0.000
10	**0.102404**	0.097929	0.1000	0.0050	0.95	0.95	0.100	1.000	1.500	0.000

rank	E_{rob}, *big*	σ_{rob}	p_1	p_2	ζ_1	ζ_2	β_1	β_2	β_3	β_4
1	**0.024000**	0.000000	0.0500	0.0250	0.90	0.50	0.050	0.500	1.000	0.010
2	**0.028000**	0.026687	0.2500	0.1000	0.99	0.95	0.500	0.500	0.500	0.000
3	**0.028000**	0.011861	0.0750	0.0250	0.99	0.99	0.050	0.500	1.000	0.000
4	**0.028000**	0.011861	0.0750	0.0250	0.99	0.99	0.050	0.500	1.000	0.001
5	**0.028000**	0.005930	0.0500	0.0250	0.90	0.95	0.050	0.500	1.000	0.000
6	**0.028000**	0.011861	0.0500	0.0250	0.95	0.50	0.050	0.500	1.000	0.010
7	**0.032000**	0.017791	0.0100	0.0500	0.25	0.25	0.050	0.500	1.500	0.000
8	**0.032000**	0.023722	0.0100	0.0500	0.50	0.25	0.050	0.500	1.500	0.001
9	**0.032000**	0.032617	0.2500	0.1000	0.99	0.95	0.500	0.500	0.500	0.001
10	**0.032000**	0.005930	0.0500	0.0250	0.90	0.90	0.050	0.500	1.000	0.000

Table S2: The ten best performing parameter sets for the *big* diameter (10 *cm*). The upper table shows parameter sets that are ordered according to increasing E_{rms} score (Figure S39*a*), and the lower table according to E_{rob} scores (Figure S39*b*). Corresponding τ_{cm}-predictions for ϱ with the first five parameter sets are shown in Figures S50 & S51 (E_{rms} score), and Figures S54 & S55 (E_{rob} score).

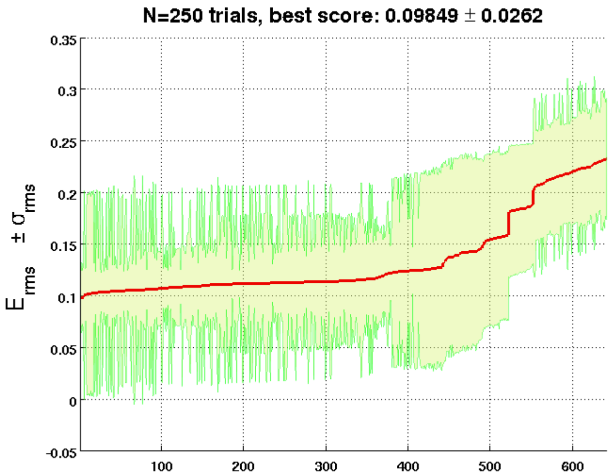

(a) $E_{rms} \pm \sigma_{rms}$ (size 10 cm)

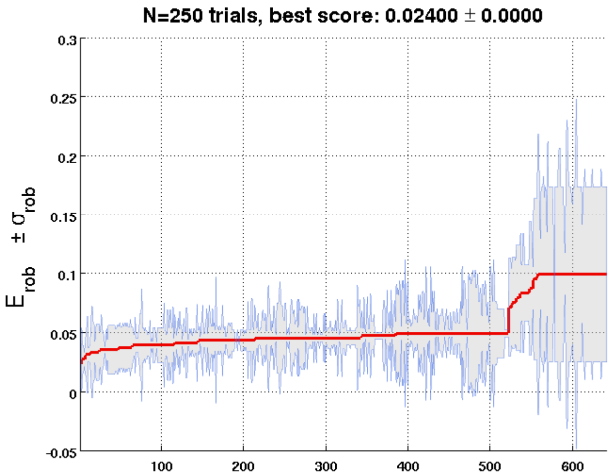

(b) $E_{rob} \pm \sigma_{rob}$ (size 10 cm)

Figure S39: τ_{cm}-scores for object diameter 10 cm (*big*). Abscissa values correspond to ranks. See legend of figure S38 for more details. The ten best performing parameter sets for *big* are listed in Table S2.

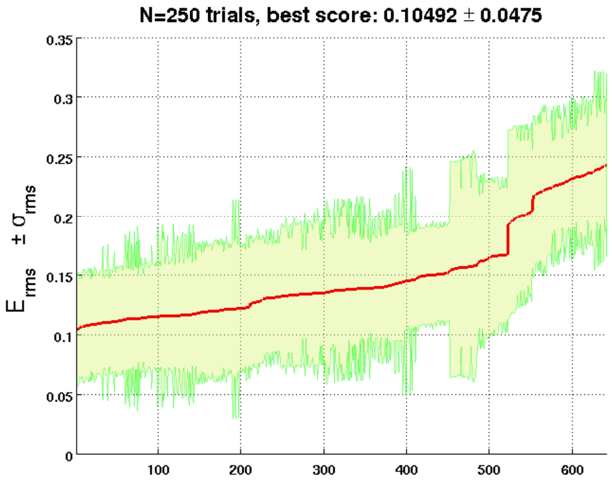

(a) $E_{rms} \pm \sigma_{rms}$ (combined size)

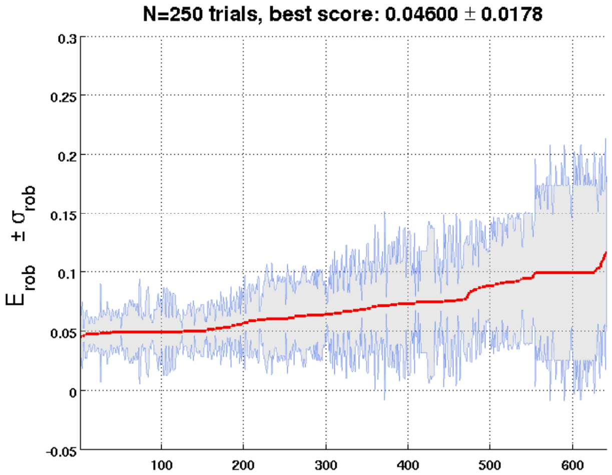

(b) $E_{rob} \pm \sigma_{rob}$ (combined size)

Figure S40: τ_{cm}-scores for *combined* diameters. Abscissa values correspond to ranks. See legend of figure S38 for more details. The ten best performing parameter sets for *combined* are listed in Table S3.

rank	E_{rms}, *both*	σ_{rms}	p_1	p_2	ζ_1	ζ_2	β_1	β_2	β_3	β_4
1	**0.104921**	0.047498	0.0750	0.0500	0.25	0.90	10.000	2.000	1.500	0.001
2	**0.105220**	0.047556	0.0750	0.0500	0.25	0.90	10.000	2.000	1.500	0.000
3	**0.105833**	0.047443	0.0750	0.0500	0.25	0.90	10.000	2.000	1.500	0.010
4	**0.106300**	0.041070	0.1000	0.0750	0.25	0.90	10.000	2.000	1.500	0.000
5	**0.106602**	0.042539	0.1000	0.0750	0.50	0.90	10.000	2.000	1.500	0.001
6	**0.106821**	0.042860	0.1000	0.0750	0.50	0.90	10.000	2.000	1.500	0.000
7	**0.106864**	0.041072	0.1000	0.0750	0.25	0.90	10.000	2.000	1.500	0.001
8	**0.107734**	0.047433	0.0750	0.0500	0.50	0.90	10.000	2.000	1.500	0.001
9	**0.107905**	0.046581	0.0750	0.0500	0.25	0.90	5.000	2.000	1.500	0.000
10	**0.107938**	0.047955	0.0750	0.0500	0.50	0.90	10.000	2.000	1.500	0.000

rank	E_{rob}, *both*	σ_{rob}	p_1	p_2	ζ_1	ζ_2	β_1	β_2	β_3	β_4
1	**0.046000**	0.017791	0.0750	0.0250	0.99	0.99	0.050	0.500	1.000	0.000
2	**0.046000**	0.025204	0.1000	0.0500	0.99	0.90	0.050	0.500	1.000	0.000
3	**0.047000**	0.028169	0.0500	0.0250	0.95	0.99	0.500	0.500	0.500	0.000
4	**0.047000**	0.005930	0.1000	0.0500	0.99	0.50	0.100	1.000	1.500	0.000
5	**0.047000**	0.005930	0.1000	0.0500	0.99	0.50	0.100	1.000	1.500	0.001
6	**0.047000**	0.019274	0.0750	0.0250	0.99	0.99	0.050	0.500	1.000	0.001
7	**0.048000**	0.019274	0.1000	0.0100	0.99	0.90	0.010	0.500	1.000	0.001
8	**0.048000**	0.020756	0.1000	0.0075	0.99	0.90	0.010	0.500	1.000	0.001
9	**0.048000**	0.014826	0.0500	0.0100	0.50	0.25	0.005	0.500	1.500	0.000
10	**0.048000**	0.014826	0.0500	0.0100	0.50	0.50	0.005	0.500	1.500	0.000

Table S3: The ten best performing parameter sets for the *combined* diameter (i.e. parameter sets for simultaneously achieving an optimal prediction performance for the object diameters *big* and *small*). The upper table shows parameter sets that are ordered according to increasing E_{rms} score (Figure S40*a*), and the lower table according to E_{rob} scores (Figure S40*b*). Corresponding τ_{cm}-predictions with the first three parameter sets are shown in Figure 8 (E_{rms} score), and Figures S56 and S57 (E_{rob} score). Notice that the first three E_{rms} scores are only distinguished by β_4.

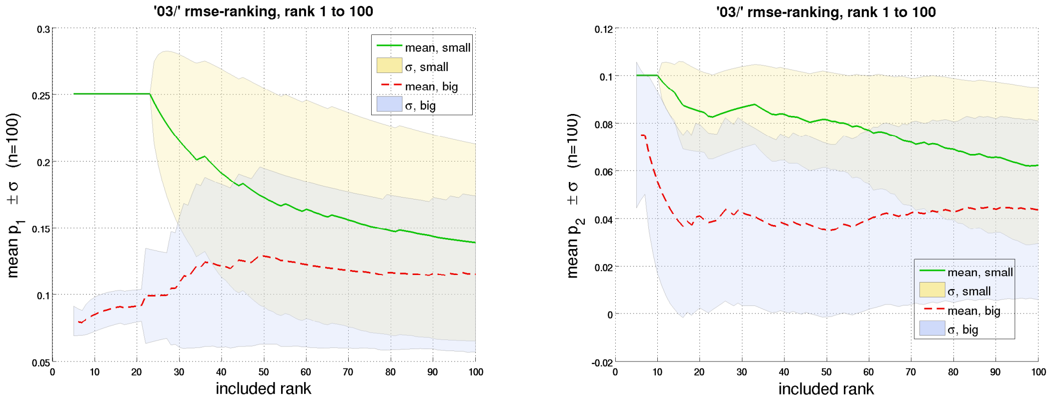

(a) mean p_1, E_{rms}

(b) mean p_2, E_{rms}

Figure S41: **Mean value of noise probabilities as a function of E_{rms} rank.** This figure shows the mean value of the noise probability equation (9) of: **(a)** angular size p_1, and **(b)** angular velocity p_2, as a function of their E_{rms} rank (see Figure 9 for more details). Abscissa values of 10, 50, etc. mean that the mean value of the first 10, first 50, etc. values of p_1 and p_2, respectively, was computed. Shaded areas indicate ±1 standard deviation. Continuous curves denote values optimized for *small* object diameter (Table S1 in *Text S5*), and broken curves denote corresponding values for the *big* diameter (Table S2 in *Text S5*).

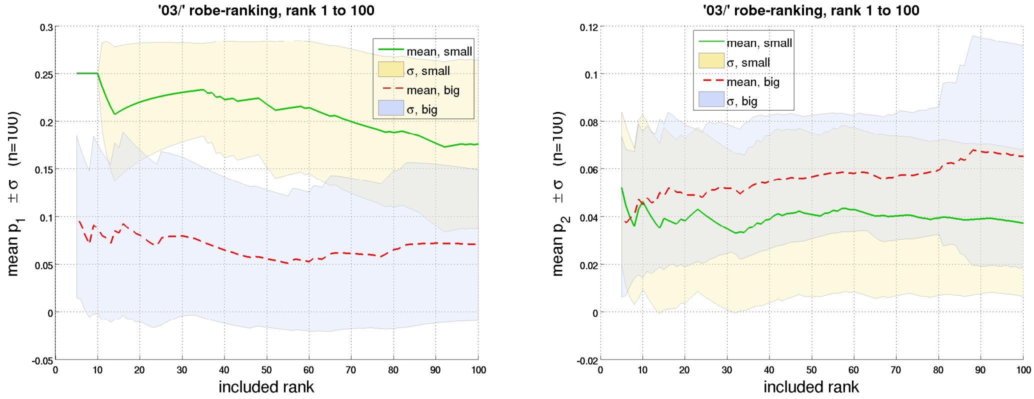

(a) mean p_1, E_{rob} (b) mean p_2, E_{rob}

Figure S42: **Mean value of noise probabilities as a function of E_{rob} rank.** Same as the previous figure, but here the parameters p_1 and p_2, respectively, were averaged according to their E_{rob} rank. Shaded areas indicate ± 1 standard deviation.

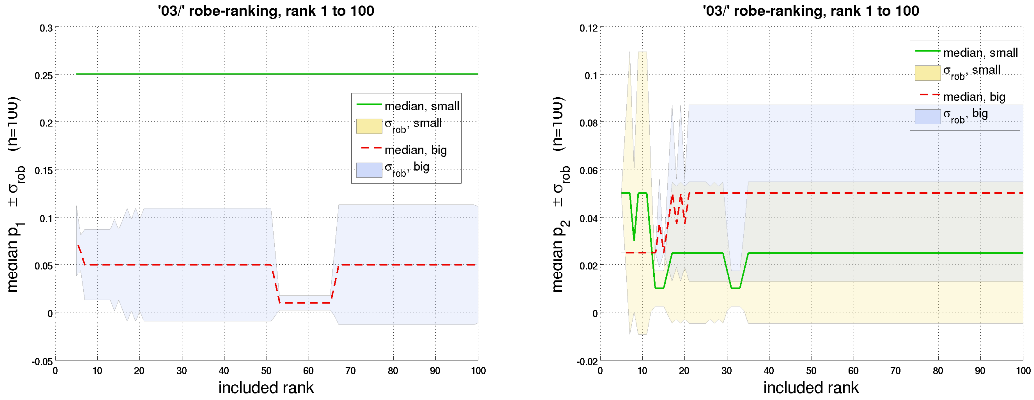

(a) median p_1, E_{rob} (b) median p_2, E_{rob}

Figure S43: **Median value of noise probabilities as a function of E_{rob} rank.** Same as Figure S41, but here the median value of the parameters p_1 and p_2, respectively, was computed according to their E_{rob} rank. The shaded areas denote $\pm 1\sigma_{rob}$ (robust estimation of standard deviation via the median absolute deviation).

References

1. Press H, Teukolsky S, Vetterling W, Flannery B (2007) Numerical Recipes: The Art of Scientific Computing, Third Edition. Cambridge University Press.
