# Supplementary material for: Unifying Time to Contact Estimation and Collision Avoidance across Species
Source: PLoS Comput Biol. 2012 Aug 16;8(8):e1002625. doi: 10.1371/journal.pcbi.1002625 (PMC3420976; doi:10.1371/journal.pcbi.1002625)
Supplement: Text S6 — Time to contact approximation of “Tau” and . Text S6 presents a comprehensive analysis of two alternative functions which have a maximum before ttc, namely “inverse ” () and “angular acceleration” (). The two functions were also fitted to the neuronal recording data (they turn out to be inadequate), and compared to the maximum of the -function. This section also provides insights into the biphasic nature of , because as approaches ttc, it gets more similar to , and thus reveals a minimum. (PDF) [file pcbi.1002625.s006.pdf]

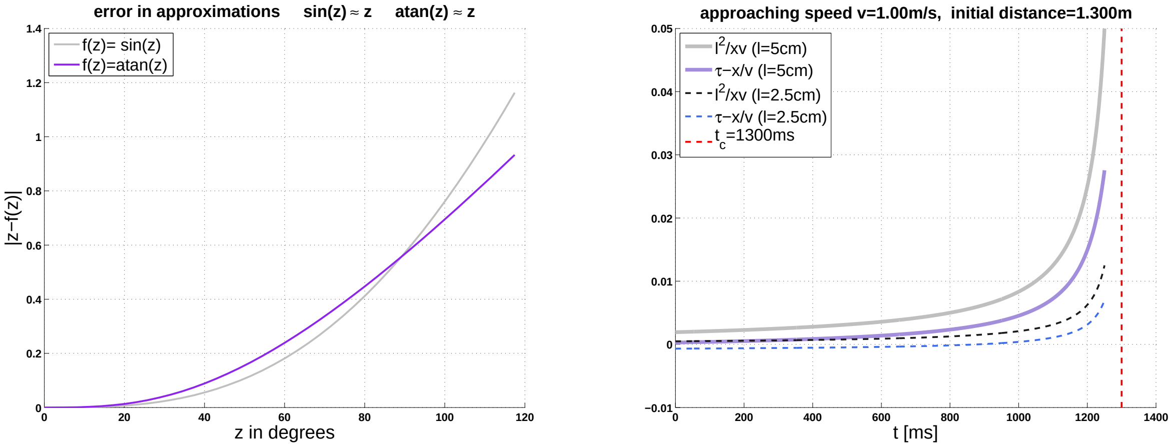

(a) absolute differences

(b)

Figure S44: **Approximating τ.** **(a)** Using the approximations $sinz \approx z$ and $\arctan z \approx z$, respectively, involves an error $|f(z) - z|$ (with $f(z) = \sin z$ or $\arctan z$, see legend) which grows with visual angle. **(b)** For two object diameters ($2l = 5cm$ and $2l = 10cm$), the functions $\tau - (t_c - t)$ and $l^2/v^2(t_c - t)$ are shown. The former one represents the deviation of τ from the quantity which it is supposed to estimate: $t_c - t$. The latter function represents the last term in equation (S21), which is neglected in the course of the approximation procedure. Bigger object diameters deteriorate the estimation $\tau \approx t_c - t$, especially in the late phase of the approach.

S6 Time to Contact Approximation of "Tau" and $\ddot{\Theta}$

The τ function estimates a running value of time to contact (t_c) until shortly before collision. Where $\tau \approx t_c - t$ holds, τ is a monotonically decreasing function, if visual angle Θ and angular velocity $\dot{\Theta}$ correspond to an object approach with constant velocity. Afterwards, when $\tau \approx t_c - t$ breaks down, τ adopts a minimum, which is accompanied by an increasing growth of the estimation error.
In the following section, we show how τ approximates ttc ("Part I"), and in the subsequent section we derive an equation for the time when τ adopts its minimum ("Part II"). We furthermore propose a "rule of thumb" for the validity period of ttc approximation, based on the maximum of angular acceleration $\ddot{\Theta}$.

S6.1 "Tau" Estimates Time to Contact (Part I)

Consider at first the series expansion for $\arctan z$ (e.g. 4.4.42, page 81 in [1]),

$$\arctan z = z \sum_{k=0}^{\infty} \frac{(-z^2)^k}{2k+1} = z - \frac{z^3}{3} + \frac{z^5}{5} - \frac{z^7}{7} + \ldots \tag{S20}$$

By the last equation, we can write $\arctan z \approx z$ for $|z| < 1$ (figure S44a). By equation (10), we identify z with $l/x(t)$, where $x(t) \equiv v \cdot (t_c - t)$. Thus, $|z| < 1$ implies small visual angles Θ, again by equation (10). Since $x(t)$ is monotonically decreasing until short before t_c, small visual angles are obtained "sufficiently far" from t_c. We will refer to the latter condition with "initial phase of the approach".
Now we plug equation (11) and the approximation $\Theta(t) \approx 2l/x(t)$ into τ:

$$\tau \equiv \frac{\Theta}{\dot{\Theta}} \approx t_c - t + \frac{l^2}{v^2(t_c - t)} \tag{S21}$$

Because $t_c - t \gg l^2/v^2(t_c - t)$ in the initial approach phase[2] (figure S44b), we finally obtain $\tau \approx t_c - t$.
□

An alternative derivation of this result was suggested by Sun & Frost [2]. Because we use a slightly different notation, we sketch their derivation here for the sake of completeness. The idea is to take the time derivative of the left hand side and the right hand side of equation (10),

$$\tan\frac{\Theta}{2} = \frac{l}{x} \quad \xrightarrow{\frac{d}{dt}} \quad \frac{\dot{\Theta}}{2\cos^2\frac{\Theta}{2}} = \frac{lv}{x^2} \tag{S22}$$

By dividing the left hand side by its temporal derivative we get (using $\sin z \approx z$ for $|z| \ll 1$, c.f. figure S44a)

$$\frac{2\sin\frac{\Theta}{2}\cos\frac{\Theta}{2}}{\dot{\Theta}} = \frac{sin\Theta}{\dot{\Theta}} \approx \frac{\Theta}{\dot{\Theta}} \equiv \tau \tag{S23}$$

By dividing the right hand side of equation (S22) by its temporal derivative, we obtain $x/v = t_c - t$. Finally, by putting things together we arrive again at $\tau \approx t_c - t$.
□

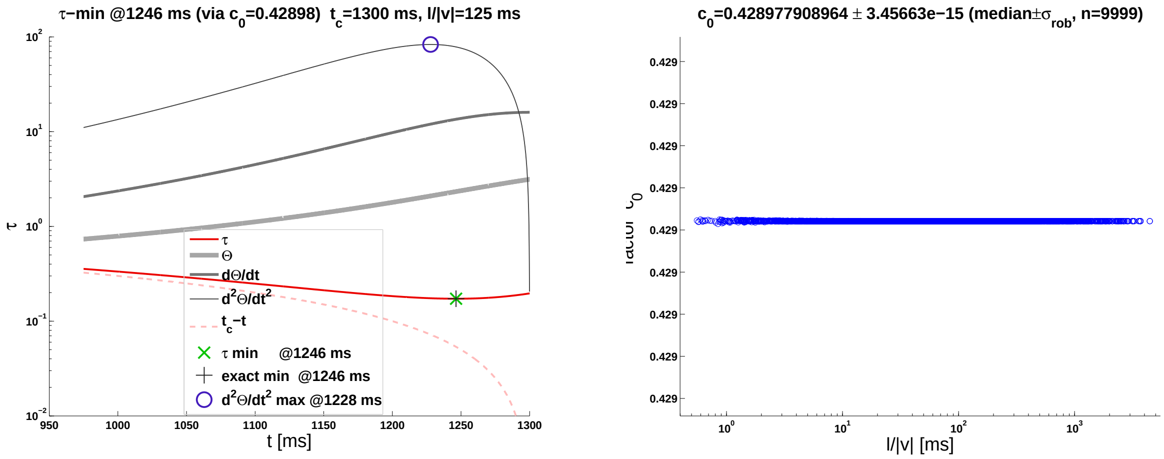

(a) absolute differences

(b)

Figure S45: τ **has a minimum.** **(a)** The nonlinear behavior of $\tau(t)$ gets prominent in the late phase of the object approach (red line). As a consequence, the deviation from $t_c - t$ increases nonlinearly (broken line). The gray lines show visual angle $\Theta(t)$, angular velocity $\dot{\Theta}(t)$, and angular acceleration $\ddot{\Theta}(t)$. The latter function has a maximum. Notice furthermore that $\dot{\Theta}(t)$ approaches a plateau shortly before t_c. The green "x" indicates the numerically determined τ-minimum. The minimum according to equation (S24) is marked by a black crosshair. Finally, the maximum of $\ddot{\Theta}(t)$ is marked by a blue circle symbol, according to equation (S26). **(b)** The time $t_{\min}$ of the minimum of τ was computed numerically via $t_{\min} : \dot{\tau}(t) \equiv 0$. To this end, 9999 values of v, t_c, and l were randomly chosen, and c_0 (equation S24) was determined. The line-distribution suggests that c_0 is constant and independent from l/v. The robust estimation of standard deviation was in the order of 10^{-15}, which most likely corresponds to numerical noise.

S6.2 Beyond ttc approximation: Minimum of "Tau" (Part II)

Given the approximation $\tau \approx t_c - t$, we may ask two questions: *(i)* Until which time do we have a "good" approximation (i.e., with acceptable small approximation error)? *(ii)* Why does the approximation get unacceptable when we get close to *ttc*?

The answer to *(ii)* is that τ reveals a minimum $t_{\min}$ shortly before *ttc* (figure S45a). By means of numerical studies we found that this minimum is located at (figure S45b)

$$t_{\min} = t_c - c_0 \cdot \frac{l}{v} \tag{S24}$$

$$c_0 = 0.428977908964 \pm 3.37433 \cdot 10^{-15}$$

$$(\text{median} \pm \sigma_{rob}, n = 9999)$$

[2] The relation $t_c - t > l^2/v^2(t_c - t)$ holds until $t = t_c - l/v$, which is nearly at impact: Notice that l/v is just the time to pass a distance that is equal to the object's halfsize or radius.

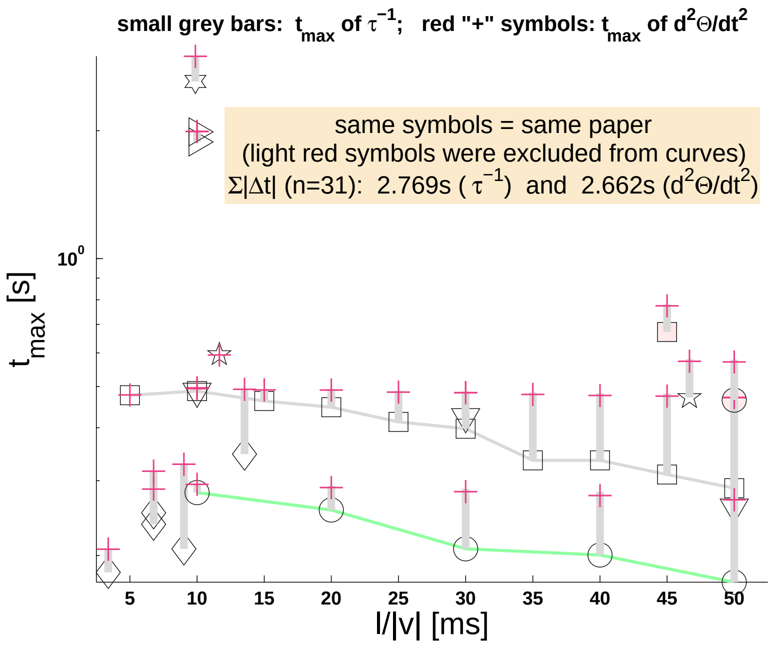

Figure S46: **Experimental $\hat{t}_{\max}$ versus predicted $t_{\max}$.** This figure is analogous to Figure 2: The symbols denote the $\hat{t}_{\max}$ of the neuronal data (*Supplementary Figure S16b*). Here, the ends of the thick, light gray bars indicate the $t_{\max}$ prediction from angular acceleration $\ddot{\Theta}$ (equation S26). The longer the bars, the higher the prediction error. The red "+" symbols indicate the $t_{\max}$ predictions from the inverse tau function τ^{-1}. Inverse tau has its maxima exactly where τ has a minimum, so corresponding predictions were computed by equation (S24). For both functions, the respective sum of absolute differences $\Delta t(\kappa_i) \equiv |\hat{t}_{\max}(\kappa_i) - t_{\max}(\kappa_i)|$ (with $\kappa \equiv l/v$) is indicated in the inset. The mean (± 1 s.d., $n = 31$) of absolute differences is $89.3\,ms \pm 80.80\,ms$ (median $\pm\sigma_{rob}$: $79.7\,ms \pm 72.48\,ms$) for τ^{-1}, and $85.9\,ms \pm 79.63\,ms$ (median $\pm\sigma_{rob}$: $75.3\,ms \pm 69.18\,ms$) for $\ddot{\Theta}$. These values correspond to the average time shift α that would be necessary to move the maxima of τ^{-1} and $\ddot{\Theta}$ to the respective $\hat{t}_{\max}$ of the experimental response curves. The two continuous lines connect the data for a series of l/v values from the same paper (gray: reference [3]; green: reference [4]). Symbols identify papers via *Supplementary Figure S16b*.

Notice that the minimum of τ is equivalent to a maximum of $1/\tau$, and that it is linearly related to the half-size to velocity ratio l/v. The maximum of the η-function is located at $t_c - \alpha \cdot l/2v$. Therefore, the minimum of $\tau(t)$ coincides with the maximum of $\eta(t)$ for $\alpha = 2c_0 \approx 0.8580$, at a constant visual angle of $2\arctan(1/2c_0) \approx 98.74°$.

Yet another function exists which has its maximum linearly related to l/v, namely angular acceleration

$$\ddot{\Theta} \equiv \frac{d^2\Theta}{dt^2} = \frac{4lv^2x(t)}{[x^2(t)+l^2]^2} \tag{S25}$$

which adopts its maximum at

$$t_{\max} = t_c - \frac{l}{\sqrt{3}v} \tag{S26}$$

because $c_0 < \sqrt{1/3} \approx 0.5774$, angular acceleration will adopt its maximum *before* τ reaches its minimum (figure S45a). The visual angle associated with the maximum of angular acceleration is approximately $81.79°$, corresponding to $\alpha = 2/\sqrt{3} \approx 1.1547$.

These values for α underestimate typical experimental values ($3 \lessapprox \alpha \lessapprox 8$, e.g. [3]). However, neither τ nor angular acceleration have an additional free parameter to "shift" the location of their minimum and maximum, respectively. The only possible way for shifting their extrema to earlier times would be by introducing a time shift δ, for example $\tau(t+\delta)$ and $\ddot{\Theta}(t+\delta)$, with some $\delta > 0$. But Figure S46 suggests that δ would be an increasing function of $\kappa = l/v$ rather than being a constant (δ is indicated by the gray bars and red "+" symbols, respectively). Moreover, the average values for δ would overestimate typical experimental values ($\lessapprox 35\,ms$, e.g. [3]).

This takes us straight to answering question *(i)*. A binary criterion for an acceptable error in the estimation $\tau \approx t_c - t$ could be based on the maximum of angular acceleration, or any other constant $c_1 > \sqrt{1/3}$. In this way one can deem the estimation as "good" as long as $t \le t_c - c_1 \cdot l/v$. As c_1 is related

to angular size, an error threshold could be based on one of the approximations S20 ($\arctan x \approx x$) or S23 ($\sin x \approx x$), respectively.
A time t_e could be defined by fixing an error $e \equiv |\tau(t_e) - t_c + t_e|$ (figure S44). Then, $\xi_e \equiv v(t_c - t_e)/l$. This ξ_e would be useful in determining t_e for arbitrary half-size to velocity ratios, that is $t_e = t_c - \xi_e l/v$.

References

1. Ambramowitz M, Stegun I (1972) Handbook of Mathematical Functions with Formulas, Graphs and Mathematical Tables. New York: Dover Publications.

2. Sun H, Frost B (1998) Computation of different optical variables of looming objects in pigeon nucleus rotundus neurons. Nat Neurosci 1: 296-303.

3. Gabbiani F, Krapp H, Laurent G (1999) Computation of object approach by a wide-field, motion-sensitive neuron. J Neurosci 19: 1122-1141.

4. Gabbiani F, Mo C, Laurent G (2001) Invariance of angular threshold computation in a wide-field looming-sensitive neuron. J Neurosci 21: 314-329.
