# Supplementary material for: Unifying Time to Contact Estimation and Collision Avoidance across Species
Source: PLoS Comput Biol. 2012 Aug 16;8(8):e1002625. doi: 10.1371/journal.pcbi.1002625 (PMC3420976; doi:10.1371/journal.pcbi.1002625)
Supplement: Text S7 — Predictions of corrected m-Tau for the psychophysical experiment. Text S7 shows the full set of figures with simulation results of our psychophysical experiment. Whereas Figure 8 shows -predictions that were obtained with the parameter set optimized for the “combined” object diameter according to -score, Text S7 shows analogous figures for the remaining parameter optimizations (big and small object diameter, and and -scores, respectively). (PDF) [file pcbi.1002625.s007.pdf]

S7 Predictions of *corrected m-Tau* for the Psychophysical Experiment

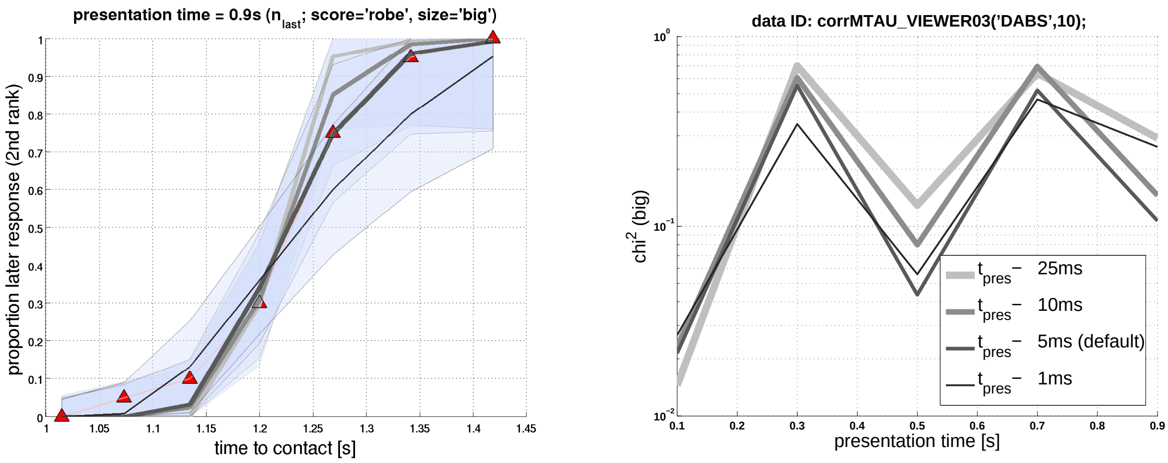

(a) $t_{\text{pres}} = 0.9$ (b) "Chi-square"

Figure S47: ***Corrected m-Tau* predictions for different n_{last} (second E_{rob} rank; *big* diameter).** For the simulation of our psychophysical study, we had to compute whether the *ttc*-prediction (equation 12) of the *corrected m-Tau*-model at t_{pres} was before or after t_{ref}. This *ttc*-prediction is computed according to equation (14), by averaging $t_c(t) \approx \tau_{\text{cm}}(t) + t$ from $t = t_{\text{pres}} - n_{last} \times 1\,ms$ to $t = t_{\text{pres}}$. **(a)** Illustration of the effect of using different averaging intervals ($n_{last} \times 1\,ms \in \{1, 5, 10, 25\,ms\}$) on model predictions ($\tau_{\text{cm}}$ parameters according to second best E_{rob} rank in Table S2 in *Text S5*). The right panel **(b)** shows "Chi-square" as a measure of "goodness-of-prediction" (section 15.1.1 in [1] – smaller values mean better predictions of psychophysical data). "Chi-square" is a standard-deviation-weighted root mean square error. The standard-deviations correspond to the blue-shaded areas in the left figure panel (see Methods Section). Standard deviations decrease with increasing t_{pres}, such that higher weighting is given to longer presentation times.

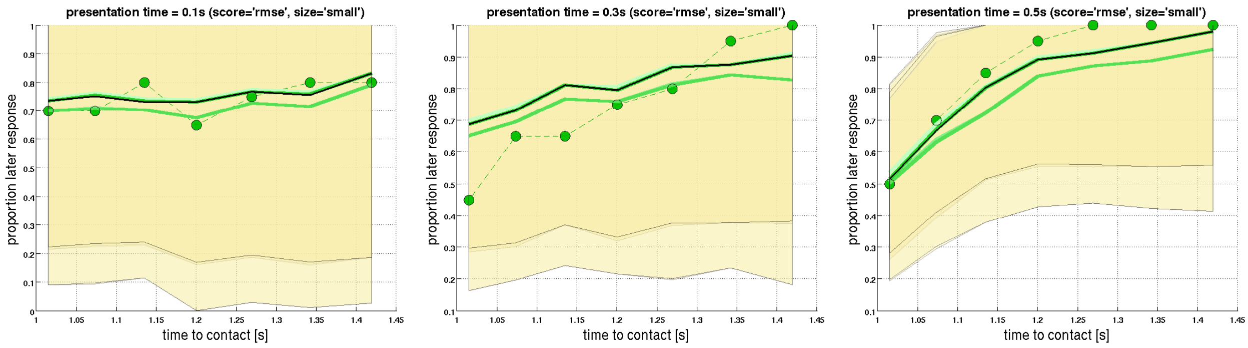

(a) $t_{\text{pres}} = 0.1$ (b) $t_{\text{pres}} = 0.3$ (c) $t_{\text{pres}} = 0.5$

Figure S48: ***Corrected m-Tau* predictions for psychophysical proportion of later response I (E_{rms} score; *small* diameter).** Analogous to Figure 8 - but here parameters were optimized for the *small* diameter, according to E_{rms} scores (Table S1 in *Text S5*).

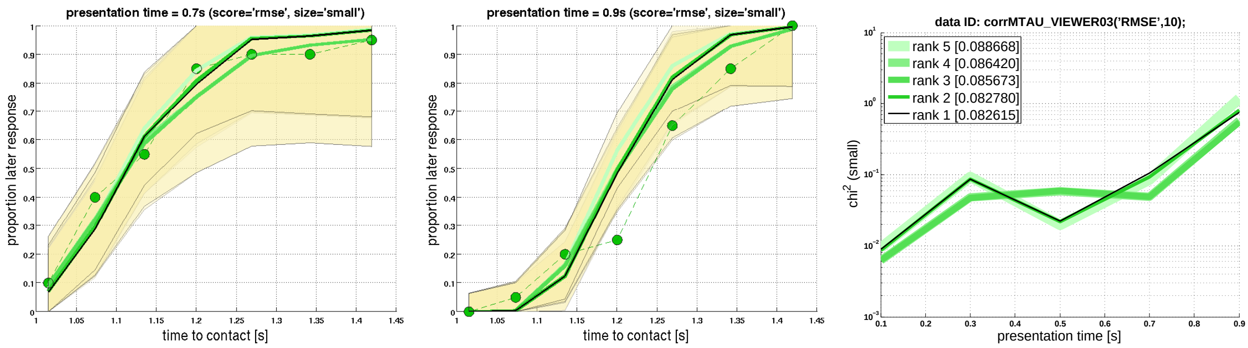

(a) $t_{\text{pres}} = 0.7$ (b) $t_{\text{pres}} = 0.9$ (c) "Chi-square"

Figure S49: ***Corrected m-Tau* predictions II** (**E_{rms} score; *small* diameter).** Same as the previous figure, but for the remaining two presentation times. **(c)** See Figure S47*b* for an explanation.

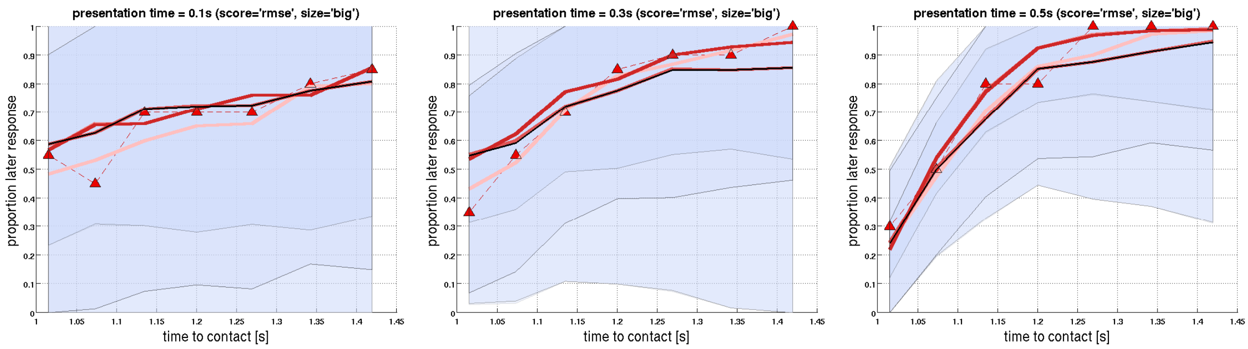

(a) $t_{\text{pres}} = 0.1$ (b) $t_{\text{pres}} = 0.3$ (c) $t_{\text{pres}} = 0.5$

Figure S50: ***Corrected m-Tau* predictions I** (**E_{rms} score; *BIG* diameter).** Analogous to Figure 8 – but here with parameters optimized for the *big* diameter, according to E_{rms} scores (Table S2 in *Text S5*).

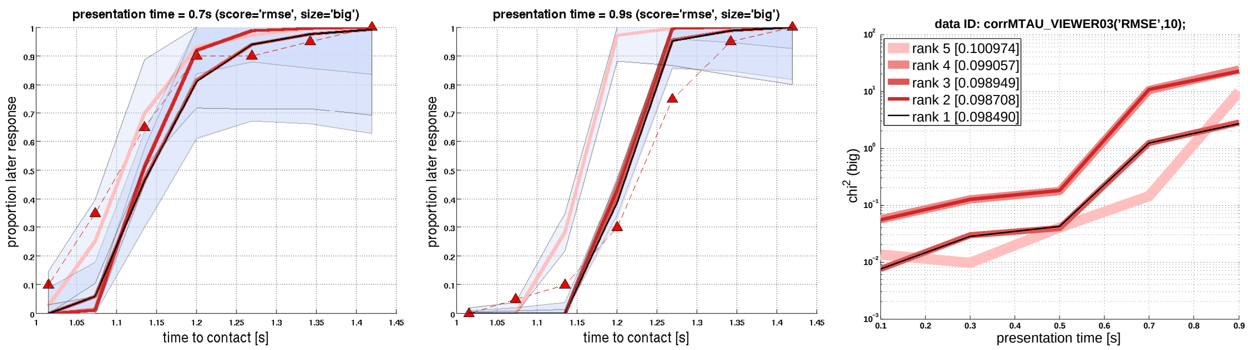

(a) $t_{\text{pres}} = 0.7$ (b) $t_{\text{pres}} = 0.9$ (c) "Chi-square"

Figure S51: ***Corrected m-Tau* predictions II** (**E_{rms} score; *BIG* diameter).** Same as the previous figure, but for the remaining two presentation times. **(c)** See Figure S47*b* for an explanation.

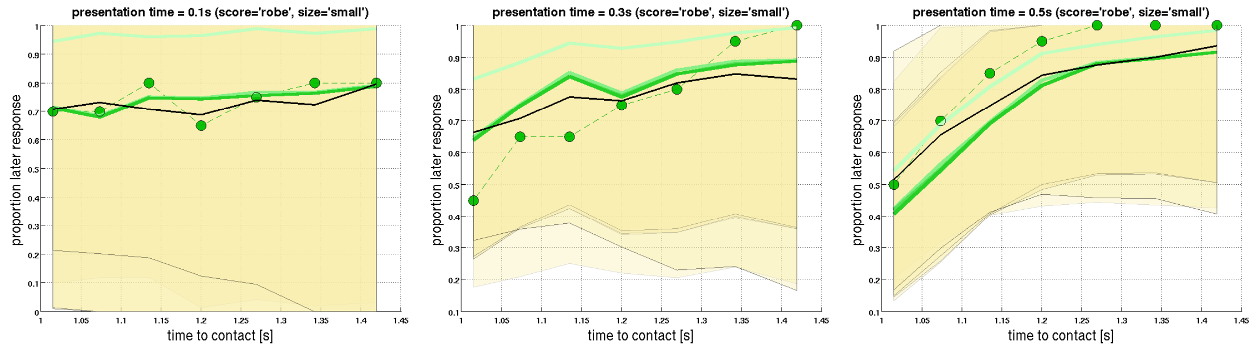

(a) $t_{\mathrm{pres}} = 0.1$ (b) $t_{\mathrm{pres}} = 0.3$ (c) $t_{\mathrm{pres}} = 0.5$

Figure S52: ***Corrected m-Tau* predictions I (E_{rob} score; *small* diameter).** Analogous to Figure 8 – but here τ_{cm}-parameters were optimized for the *small* diameter, according to E_{rob} scores (Table S1 in *Text S5*).

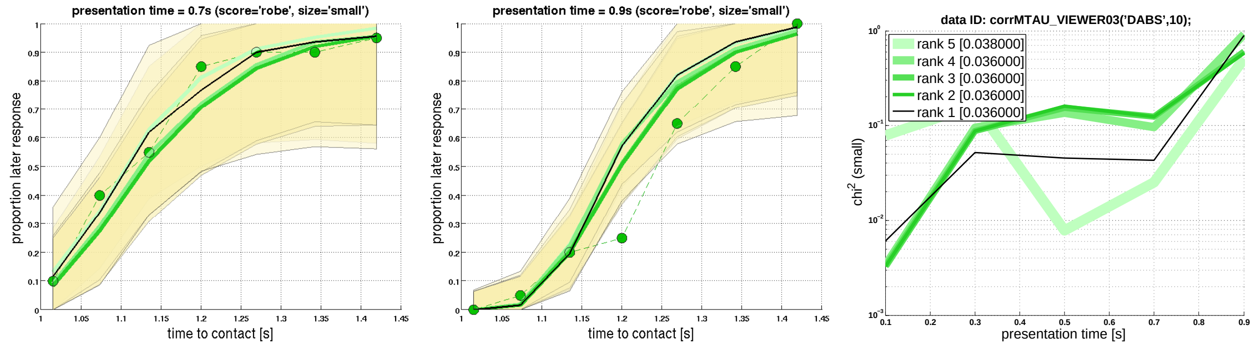

(a) $t_{\mathrm{pres}} = 0.7$ (b) $t_{\mathrm{pres}} = 0.9$ (c) "Chi-square"

Figure S53: ***Corrected m-Tau* predictions II (E_{rob} score; *small* diameter).** Same as the previous figure, but for the remaining two presentation times. **(c)** See Figure S47*b* for an explanation.

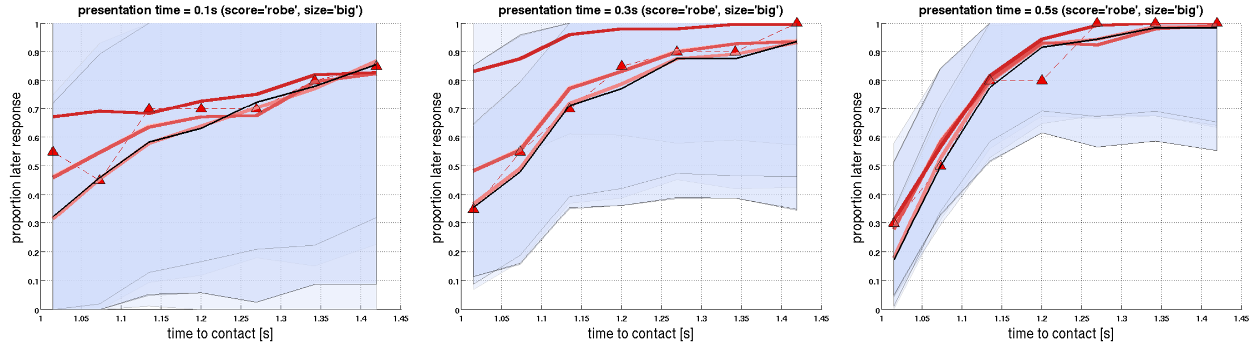

(a) $t_{\mathrm{pres}} = 0.1$ (b) $t_{\mathrm{pres}} = 0.3$ (c) $t_{\mathrm{pres}} = 0.5$

Figure S54: ***Corrected m-Tau* predictions I (E_{rob} score; *BIG* diameter).** Analogous to Figure 8 - but here parameters were optimized for the *big* diameter, according to E_{rob} scores (Table S2 in *Text S5*).

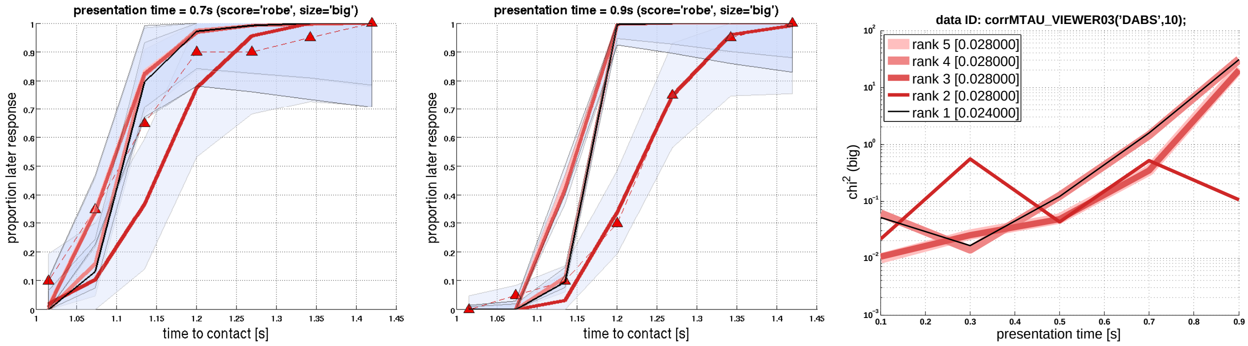

(a) $t_{\text{pres}} = 0.7$ (b) $t_{\text{pres}} = 0.9$ (c) "Chi-square"

Figure S55: ***Corrected m-Tau* predictions II (E_{rob} score; *BIG* diameter).** Same as the previous figure, but for the remaining two presentation times. **(c)** See Figure S47*b* for an explanation.

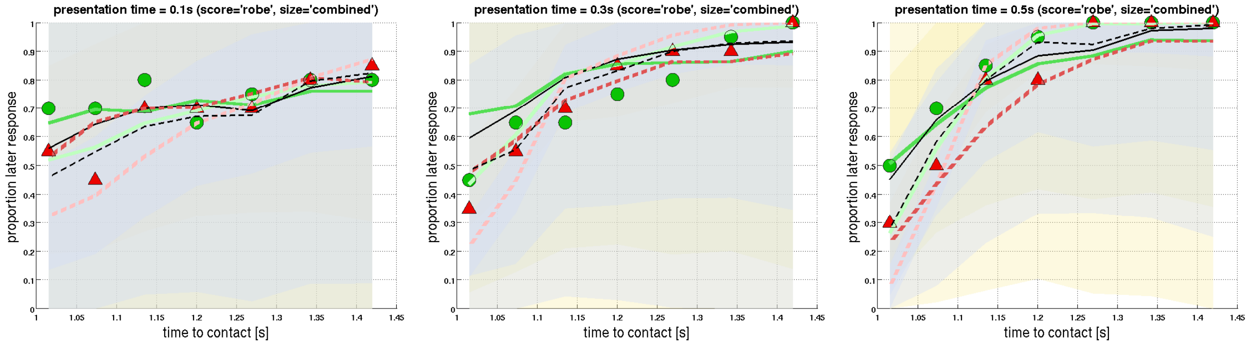

(a) $t_{\text{pres}} = 0.1$ (b) $t_{\text{pres}} = 0.3$ (c) $t_{\text{pres}} = 0.5$

Figure S56: ***Corrected m-Tau* predictions I (E_{rob} score; *combined* diameter).** Analogous to Figure 8 - but here with the three best performing parameter sets according to E_{rob} scores (Table S3 in *Text S5*). Notice that the τ_{cm}-predictions for both object diameters were computed with the same parameter set.

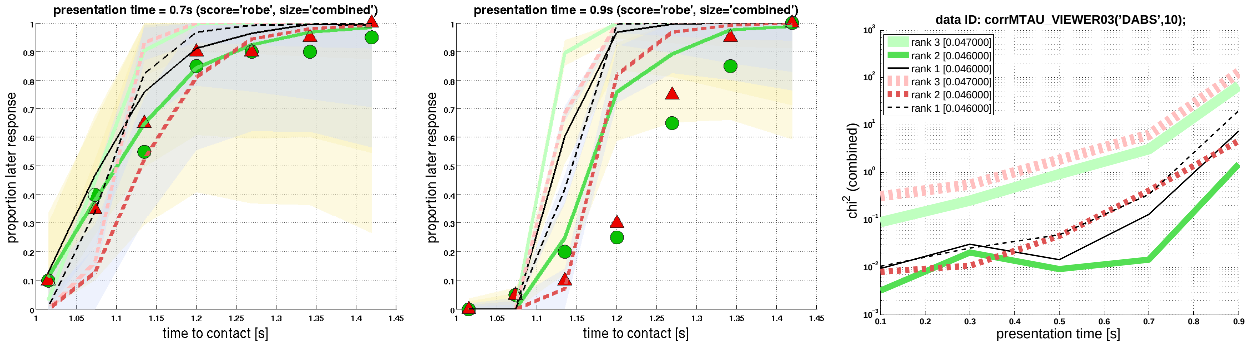

(a) $t_{\text{pres}} = 0.7$ (b) $t_{\text{pres}} = 0.9$ (c) "Chi-square"

Figure S57: ***Corrected m-Tau* predictions II (E_{rob} score; *combined* diameter).** Same as the previous figure, but for the remaining two presentation times. **(c)** See Figure S47*b* for an explanation.

References

1. Press H, Teukolsky S, Vetterling W, Flannery B (2007) Numerical Recipes: The Art of Scientific Computing, Third Edition. Cambridge University Press.
