# Supplementary material for: Unifying Time to Contact Estimation and Collision Avoidance across Species
Source: PLoS Comput Biol. 2012 Aug 16;8(8):e1002625. doi: 10.1371/journal.pcbi.1002625 (PMC3420976; doi:10.1371/journal.pcbi.1002625)
Supplement: Text S8 — First order temporal low-pass filter ( Equation 4 ). Text S8 gives a short introduction to the temporal low-pass filter that forms a part of the -model (equation 4), and is also used for the extension of the -model described in Text S1). (PDF) [file pcbi.1002625.s008.pdf]

S8 First Order Temporal Low-Pass Filter (Equation 4)

This section derives equation (4) from the differential equation of a leaky integrator. Let x be the state variable of the leaky integrator which, for example, may represent the firing rate of a neuron:

$$\tau_m \frac{dx(t)}{dt} = -x(t) + z(t) \tag{S27}$$

The last equation integrates the input $z(t)$ with time constant τ_m (which may represent the membrane time constant of a neuron). If τ_m is sufficiently small, then past inputs are quickly discarded, and the filter response $x(t)$ ("output variable") eventually follows the input $z(t)$. In other words, few low-pass filtering of $z(t)$ occurs, and the filter is said to have a short memory.
If τ_m is very big, then the opposite will occur: The filter gets very sluggish, and eventually sums up all inputs $z(t)$. This means that the filter output $x(t)$ is a strongly low-pass filtered version of the input $z(t)$, and the filter is said to have a long memory.
The just described behavior is readily seen when we consider a discretized version of the last equation. For discretization, we assume that time t increases in steps of Δt ("sampling interval" or "integration time step"). We have two possibilities for implementing discretizaton: *Forward differencing* and *backward differencing*. Both differencing schemes will be considered in turn.

S8.1 Forward Differencing ("Forward Euler")

Here, the right hand side depends on t (i.e., only on past terms):

$$\tau_m \frac{x(t+\Delta t) - x(t)}{\Delta t} = -x(t) + z(t) \tag{S28}$$

By Rearranging terms we obtain:

$$x(t+\Delta t) = \left[1 - \frac{\Delta t}{\tau_m}\right] x(t) + \frac{\Delta t}{\tau_m} z(t) \tag{S29}$$

Now let $\xi \equiv \Delta t/\tau_m$, and $0 \leq \xi \leq 1$. Then, we readily obtain equation (4) by defining the memory constants as $\zeta_i \equiv 1 - \xi$ with $i = 1, 2$. A big time constant $\tau_m \gg \Delta t$ implies $\zeta_i \to 1$. This would endow the filter with an infinite memory – it will never change its initial value, because the input $z(t)$ will be multiplied by zero.
The other limit case is defined by $\tau_m = \Delta t$ ("small τ_m"), and thus $\zeta_i = 0$. Then, $x(t + \Delta t) = z(t)$, meaning that the filter has no memory on past inputs. In other words, no lowpass filtering takes place – the filter output x follows the input signal z.

S8.2 Backward Differencing ("Backward Euler")

Here, the right hand side depends on $t + \Delta t$ (i.e., on future terms):

$$\tau_m \frac{x(t+\Delta t) - x(t)}{\Delta t} = -x(t+\Delta t) + z(t+\Delta t) \tag{S30}$$

A more compact notation can be obtained by susbtituting $\tilde{t} \equiv t + \Delta t$ in the last equation (and omit the tilde in what follows):

$$\tau_m \frac{x(t) - x(t-\Delta t)}{\Delta t} = -x(t) + z(t) \tag{S31}$$

By Rearranging terms we obtain:

$$x(t) = \frac{\tau_m}{\tau_m + \Delta t} x(t-\Delta t) + \frac{\Delta t}{\tau_m + \Delta t} z(t) \tag{S32}$$

For backward differencing, the filter memory constants ζ_i ($i = 1, 2$) from equation (4) are defined by $\zeta_i \equiv \tau_m/(\tau_m + \Delta t)$. Notice that $1 - \zeta_i = \Delta t/(\tau_m + \Delta t)$, which is the factor associated with the input z. For big time constants $\tau_m \gg \Delta t$ we get $\zeta_i \to 1$, meaning that our filter would approach an infinite memory (strong lowpass filtering).
For small values $\tau_m = 0$, we obtain $\zeta_i = 0$ and thus $x(t) = z(t)$ – the filter has no memory on past inputs, and consequently no lowpass filtering will take place.
